# mTOR signaling networks: mechanistic insights and translational frontiers in disease therapeutics

**DOI:** 10.1038/s41392-025-02493-4

**Published:** 2025-12-30

**Authors:** Hanxiao Zhang, Xia Xiao, Zhenrui Pan, Svetlana Dokudovskaya

**Affiliations:** 1 CNRS UMR9018, Université Paris-Saclay, Gustave Roussy, Villejuif, France; 2 Department of Pathophysiology, School of Basic Medical Sciences, College of Medicine, Zhengzhou University, Zhengzhou, Henan China

**Keywords:** Cell biology, Cancer metabolism

## Abstract

The mammalian target of rapamycin (mTOR) pathway is a central regulator of cellular growth, metabolism, and homeostasis, integrating a wide array of intracellular and extracellular cues, including nutrient availability, growth factors, and cellular stress, to coordinate anabolic and catabolic processes such as protein, lipid, and nucleotide synthesis; autophagy; and proteasomal degradation. The dysregulation of this signaling hub has broad implications for health and disease. To commemorate the 50th anniversary of the discovery of rapamycin, we provide a comprehensive synthesis of five decades of mTOR research. This review traces the historical trajectory from the early characterization of the biological effects of rapamycin to the elucidation of its molecular target and downstream pathways. We integrate fundamental and emerging insights into the roles of mTOR across nearly all domains of cell biology and development, with a particular focus on the expanding landscape of therapeutic interventions targeting this pathway. Special emphasis is placed on the crosstalk between mTOR signaling and mitochondrial regulation, highlighting the mechanisms by which these two metabolic hubs co-regulate cellular adaptation, survival, and disease progression. The dynamic interplay between mTOR and mitochondrial networks governs key aspects of bioenergetics, redox balance, and cell fate decisions and is increasingly implicated in pathophysiological contexts ranging from cancer and aging to neurodegenerative and immune disorders.

## Introduction

**Fig. 1:**
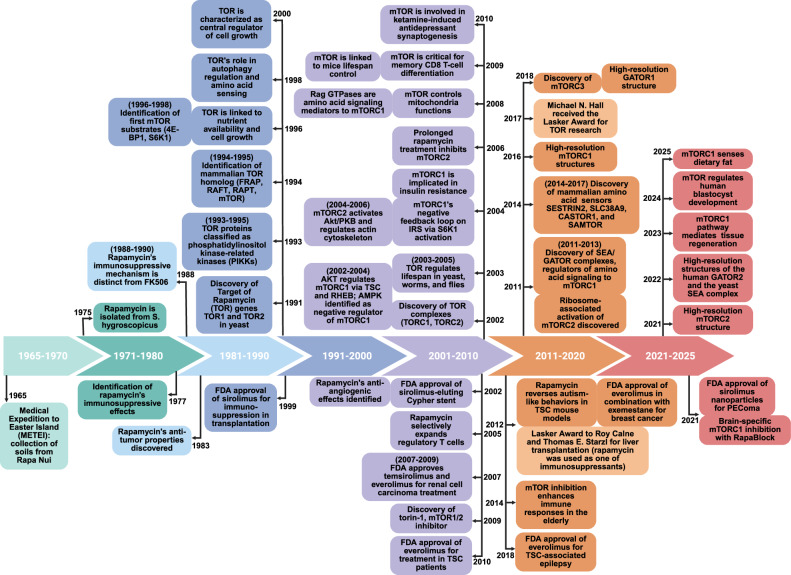
Historical overview of mTOR research and its clinical application. The timeline illustrates key discoveries in the mTOR signaling pathway (upper panel) and parallel progress in the identification and clinical application of mTOR-targeting agents (lower panel). See the text for details

The year 2025 marks two major anniversaries that forever changed the fields of metabolism and cell signaling: the 50th anniversary of the discovery of rapamycin in 1975^1,2^ and the 60th anniversary of the Medical Expedition to Easter Island (METEI) in 1964–1965,^3^ during which soil samples, containing *Streptomyces hygroscopicus*, the bacterium that produces rapamycin, were collected. Although fewer than a dozen papers were published on rapamycin in the first ten years following its discovery, the subsequent decades witnessed a significant expansion of the field (Fig. 1). This growth was driven by the identification of the immunosuppressive^4^ and anticancer properties of rapamycin,^5^ as well as the discovery of its molecular target, a protein named “target of rapamycin” (TOR), first in yeast^6,7^ and three years later, its homolog in mammals, subsequently referred to as mammalian TOR (mTOR).^8–11^

Today, research on TOR/mTOR spans virtually all key aspects of cellular function and development. mTOR represents a major focus in clinical and pharmaceutical research because of the deregulation of mTOR pathway in numerous human diseases, including neurodegenerative disorders and cancer.

This review summarizes five decades of TOR/mTOR research (hereafter referred to as mTOR unless describing the work in yeast or Drosophila), covering molecular mechanisms and highlighting recent successes in targeting components of the mTOR pathway in clinical trials. Special emphasis will be placed on the interplay between mTOR signaling and mitochondrial regulation, illustrating how these two metabolic hubs collaborate to sustain cellular proliferation and survival.

## History of mTOR research

The discovery of the “wonder drug” rapamycin is perhaps the most famous outcome of the METEI, organized by the Canadian scientist Stanley Skorina, initially to evaluate the global health of Easter Island’s population and environmental challenges on the island, with the indigenous name Rapa Nui. One of METEI’s members, microbiologist Georges Nógrády, divided the island into 64 squares and collected soil samples from each. Upon returning to Canada, he generously distributed these samples to many colleagues, both nationally and internationally.^3,12^ Some of the soil samples reached the bacteriologist Claude Vézina and the biochemist Surendra (Suren) Sehgal at Ayerst Research Laboratories in Canada. Between 1975 and 1979, they published a series of articles describing the isolation of a *Streptomyces hygroscopicus* strain from these soil samples, from which they extracted a compound with antifungal properties^1,2,13,14^ and immunosuppressive potential.^4^ The compound was named rapamycin to acknowledge its origin from Rapa Nui Island. In 1984, rapamycin was found to exhibit strong anticancer activity against many solid tumors.^5^ Importantly, rapamycin was cytostatic, in contrast to most anticancer therapies at the time, which were cytotoxic. The structure of rapamycin was solved by X-ray crystallography in 1978,^15^ allowing it to be classified as a macrolide. Another macrolide, FK506, was already known for its immunosuppressive properties, being successful as a transplant drug (tacrolimus), but its mechanism of action differed from that of rapamycin.^16^ Efforts to understand these differences ultimately led to the discovery of the molecular target of rapamycin.

In 1991, a group led by Michael Hall identified two genes, *TOR1* and *TOR2*, that mediate rapamycin sensitivity in the yeast *Saccharomyces cerevisiae*.^6^ Unlike the two other immunosuppressants, FK506 and cyclosporin A, whose immunophilin complexes inhibit calcineurin, the rapamycin–FKBP12 complex does not interfere with calcineurin but instead directly binds to and inhibits a protein that was named “target of rapamycin” (TOR). About the same time, the groups of George Livi^17^ and John J. Siekierka^18^ independently isolated FKBP12 (designated RBP1 or FKB1, respectively), demonstrating its role as a mediator of rapamycin and FK506 sensitivity in yeast^19^ and identifying the yeast TOR gene.^20^

The discovery of the mammalian homolog of TOR followed approximately three years later, by four laboratories led by Stuart L. Schreiber,^8^ Sol H. Snyder,^9^ Vivian Berlin,^10^ and Robert T. Abraham.^11^ Although each group assigned a different name to the protein (FRAP,^8^ RAFT1,^9^ or RAPT1^10^), the term mTOR^11^ for “mammalian target of rapamycin” ultimately became the accepted standard in mammals and other eukaryotes.

Since the mid-1990s, research on TOR/mTOR has accelerated tremendously (Fig. 1), with over 5000 papers published annually over the past decade on TOR, rapamycin, its derivatives and clinical applications. This growing body of work has advanced along two main fronts: first, elucidating the molecular mechanisms of mTOR signaling pathways^21^; second, developing therapeutic strategies to modulate mTOR activity in a wide range of human diseases^22^ (Fig. 1). In the sections below, we review the major achievements in both areas.

## Structures of mTOR and its complexes

In yeast, there are two *TOR* genes, *TOR1* and *TOR2*,^6,23^ whereas mammalian cells contain only one *mTOR* gene. The Tor1 and Tor2 proteins share 67% amino acid identity and 85% similarity. The overall amino acid identity between *S. cerevisiae* Tor1/Tor2 and human mTOR is approximately 40–45%.

Tor/mTOR is a serine/threonine kinase with a molecular weight of approximately 289 kDa and is classified within the phosphoinositide 3-kinase-related kinase (PIKK) family owing to structural similarities in its catalytic domain.^24,25^ The N-terminal region of mTOR contains approximately 20 HEAT (huntingtin, elongation factor 3, protein phosphatase 2 A, TOR1) repeats, which are involved in mediating protein‒protein interactions. Its C-terminus is composed of conserved domains, including the FAT domain (shared by the FRAP, ATM, and TRRAP proteins) and the FKBP12-rapamycin binding (FRB) domain, which facilitates binding of the FKBP12–rapamycin complex, the kinase catalytic domain, and the FATC domain located at the extreme C-terminus. The FAT and FATC domains modulate the activity of the kinase domain.

In yeast, Tor1 is a part of TOR complex 1 (TORC1) or TOR complex 2 (TORC2), whereas the Tor2 protein is a component of TOR complex 2.^26^ In mammals, the mTOR protein is a part of three protein complexes: mTORC1, mTORC2, and the newly identified and currently poorly characterized mTORC3,^27–29^ of which only mTORC1 is sensitive to acute rapamycin treatment.

**Fig. 2:**
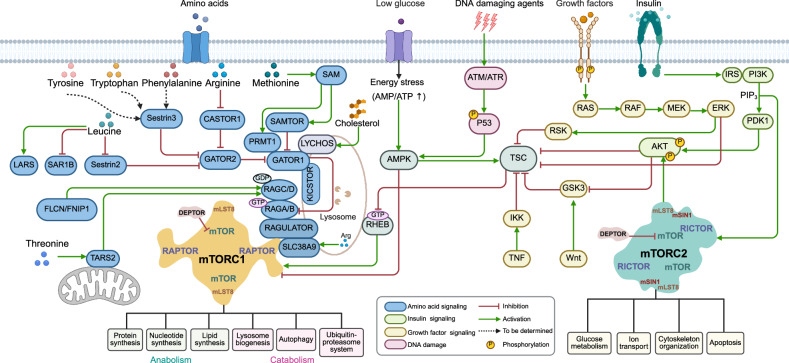
Overview of the mTORC1 and mTORC2 signaling pathways. The mTORC1 and mTORC2 complexes are depicted as profiles of their respective dimeric structures. Various stimuli, upstream regulators, and downstream effectors are illustrated. Amino acids, glucose, growth factors and DNA damaging agents modulate mTORC1 activity to control anabolic and catabolic processes. Insulin activates PI3K to generate PIP_3_, which activates mTORC2. The precise targets of mTORC1 and mTORC2 and their phosphorylation sites are listed in Table 1

The mTORC1 core consists of mTOR, mLST8 and regulatory associated protein of mTOR (RAPTOR),^30^ whereas the mTORC2 core contains mTOR, mLST8, mSIN1, and rapamycin-insensitive companion of mTOR (RICTOR)^31^ (Figs. 2, 10). mLST8 interacts with the kinase domain of mTOR; RAPTOR interacts with HEAT repeats.^28^ mTOR complexes associate with other proteins that are generally not considered part of the core, such as DEPTOR, an endogenous inhibitor of both mTOR complexes, and PRAS40, an endogenous inhibitor of mTORC1. In mammals, mTORC2 also interacts with the PROTOR protein, which has two isoforms: PROTOR1 (PRR5) and PROTOR2 (PRR5L). The functions of these isoforms are not well defined.

mTORC1 adopts a rhomboid configuration, measuring approximately 280 × 210 × 130 Å,^32^ which results from the dimerization of a heterodimer consisting of mTOR, mLST8, and RAPTOR. Within this complex, RAPTOR interacts with the N-terminal region of mTOR, playing a critical role in complex assembly, subcellular localization, stability, and substrates recruitment. PRAS40 is located near the central core and acts as a negative regulator of mTORC1 signaling. mLST8 associates with the kinase domain of mTOR and contributes to maintaining its enzymatic stability. DEPTOR is another inhibitory partner within the complex. Furthermore, the rapamycin-FKBP12 complex binds to the FRB domain of mTOR, effectively blocking substrate access to the catalytic site.

The mTORC2 complex is also organized as a dimer composed of two heterotetrameric units, showing a hollow rhombohedral fold with dimensions of ~220 × 200 × 130 (Å).^32,33^ Like mTORC1, the mTOR-mLST8 heterodimer forms the structural core of mTORC2. The two mTOR molecules interact directly, establishing a central scaffold that facilitates the attachment of additional subunits. mLST8, mSIN1, and RICTOR each bind symmetrically to the mTOR dimer in two copies. RICTOR plays a key role in mTORC2 assembly, substrate specificity, and structural stability by forming extensive interactions with mTOR. Importantly, the position of RICTOR within mTORC2 explains the insensitivity of the complex during acute treatment with rapamycin because the FRB domain of mTOR in mTORC2 is sterically hindered by RICTOR, preventing the FKBP12–rapamycin complex from binding.

mSIN1 connects RICTOR to mLST8 through its N-terminal region and serves as a scaffold that mediates interactions with serum and glucocorticoid-regulated kinase 1 (SGK1) while also modulating mTORC2 kinase activity in a negative manner. DEPTOR functions as an inhibitor by binding to mTOR. PROTOR-1, which is associated with RICTOR, contributes to the regulation of SGK1 phosphorylation driven by mTORC2.

mTOR can also interact via its FRB domain and LST8 binding element with the E26 transformation-specific transcription factor ETV7 within the cytoplasm. This interaction results in the formation of mTORC3, a complex that exhibits dual mTORC1 and mTORC2-like signaling activities yet functions independently of the typical components associated with mTORC1 or mTORC2.^29^ To date, the mTORC3 complex has been described by only one research group laboratory and will not be discussed further in this review.

Both mTORC1 and mTORC2 localize to multiple subcellular compartments, but mTORC1 is present mainly at the lysosomal membrane^34^ and partially at the Golgi,^35^ while mTORC2 can be found at the plasma membrane, outer mitochondrial membrane, and endosomal vesicles.^36,37^

Currently, approximately 60 direct substrates of mTORC1 and approximately 30 substrates of mTORC2 have been identified, most of which regulate transcription, translation, and autophagy^38^ (Table 1). Notably, the mTORC1 substrates include ribosomal protein S6 kinase 1 (S6K1) and eIF4E binding protein (4E-BP1), which play key roles in translation^22,39–41^; the transcription factors EB and E3 (TFEB/TFE3), which regulate lysosomal gene expression^42–45^; and Unc-51-like kinase 1 (ULK1),^46^ a crucial initiator of autophagy. mTORC2 signaling controls various cellular functions through its regulation of AGC family kinases, such as AKT, protein kinase C (PKC), and serum and glucocorticoid-induced kinases (SGKs).^28^

**Table 1:**
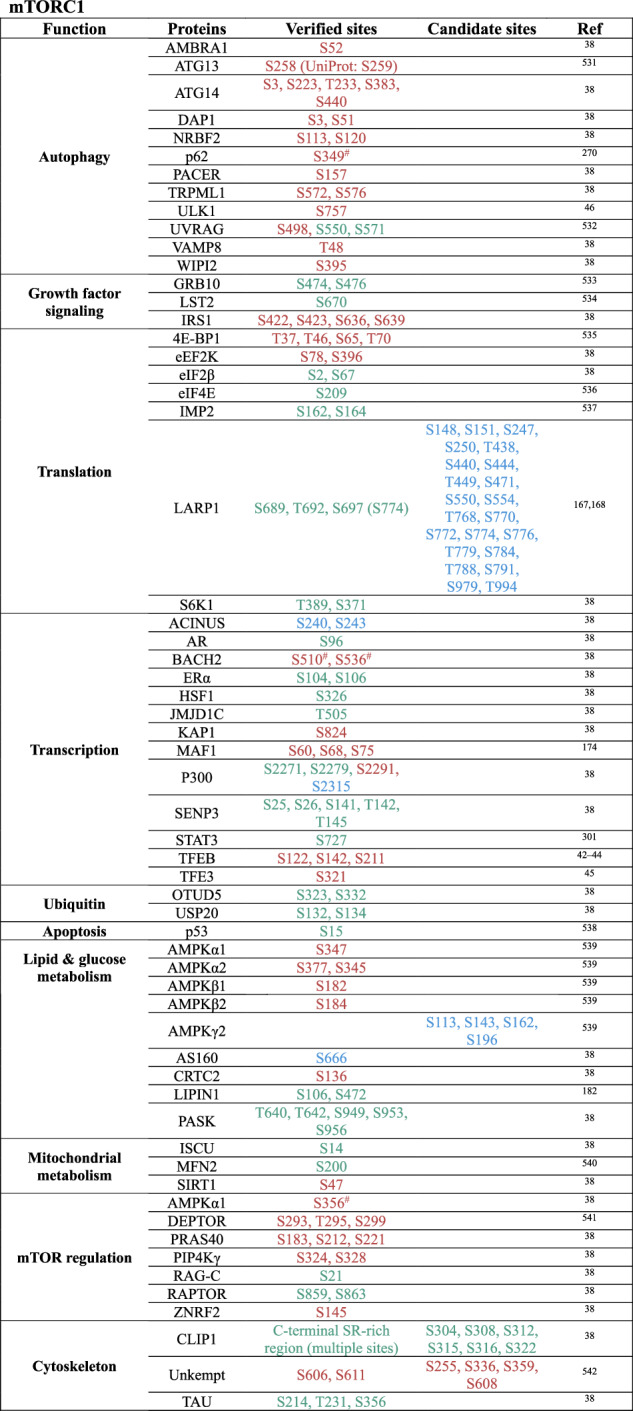

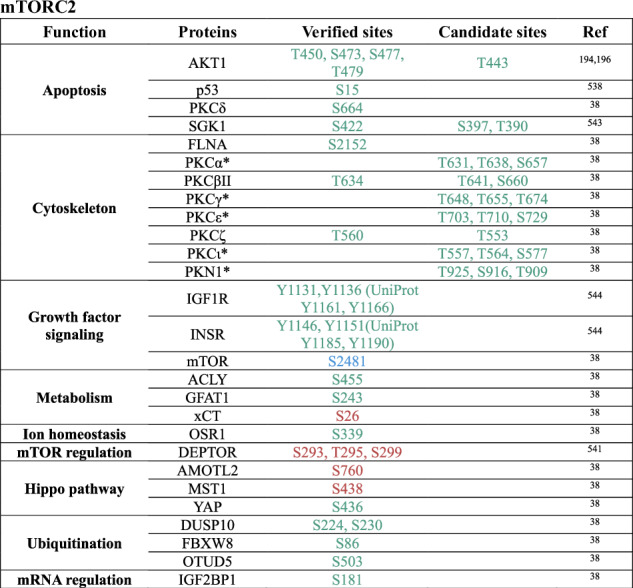
Direct substrates and phosphorylation sites of mTOR complexes

Notes: Phosphorylation sites leading to the inhibition of a corresponding protein are indicated in , those leading to activation are indicated in , and sites with unknown effects are indicated in . Only human phosphorylation sites are listed. * refers to sites not verified experimentally.^38^^#^ Sites reported in Battaglioni et al.,^38^ where the residue numbering does not match the original primary publication because Battaglioni et al.^38^ used UniProt-based annotation instead of the numbering applied in the original study. S - serine, T - threonine, Y - tyrosine.^38,42–46,167,168,174,182,194,196, 270,301,531–544^

## Regulation of nutrient and stress signaling upstream of mTORC1

To ensure a precise and timely response to both extra and intracellular cues, mTORC1 integrates upstream signals through two major axes regulated by small GTPases — RHEB (Ras homolog enriched in the brain) and RAGs (Ras-related small GTP-binding proteins). RAGs function as heterodimers: RAGA or RAGB bind to RAGC or RAGD. Amino acid availability is primarily conveyed to mTORC1 via the GATOR1/GATOR2-RAG signaling axis,^28,47^ whereas growth factors and energy activate mTORC1 through the TSC-RHEB axis (Fig. 2).^28,48,49^

In amino acid sufficiency, RAGA/B is bound to GTP, whereas RAGC/D is bound to GDP.^50,51^ RAGs promote the recruitment of mTORC1 to the lysosomal membrane, where mTORC1 is fully activated by RHEB and bound to GTP. Conversely, when amino acids are low, RAGs are inactive, and RAGA/B is loaded with GDP, while RAGC/D is bound to GTP.

RAGs are not functionally redundant.^52,53^ Thus, RAGD preferentially promotes mTORC1 phosphorylation of TFEB, which is linked to a stronger association of RAGD with the lysosome than RAGC, whereas TFE3 is preferentially recruited by RAGC.^53^ In contrast, both RAGC and RAGD equally regulate the nonlysosomal mTORC1 substrates S6K1 and 4E-BP1.^52^ Thus, S6K and 4E-BP1 were named “canonical” (nonlysosomal, RHEB-TSC-dependent), whereas TFEB/TFE3 are “noncanonical” (lysosomal, RHEB-TSC-independent) mTORC1 substrates.^54^ The RAGA and RAGB also differ from each other. Moreover, RAGB has two isoforms: a “short” isoform, which is prevalent in most tissues, and a “long” isoform, which is expressed primarily in neurons. Both isoforms, unlike RAGA, alter the dynamics of mTORC1 activity, allowing it to persist even under amino acid–depleted conditions.^55^

GTPases are dependent on their regulators, GTPase-activating proteins (GAPs), which stimulate GTP hydrolysis, and guanine nucleotide exchange factors (GEFs). The GATOR1 complex acts as a GAP for RAGA, thereby transforming RAGA to its inactive, GDP-bound form, which leads to mTORC1 suppression.^47^ A complex between folliculin (FLCN) and folliculin-interacting protein (FNIP) is also a GAP, but for RAGC/D. FLCN/FNIP transforms RAGC/D to the active GDP conformation, leading to mTORC1 activation.^56^ Finally, growth factors activate RHEB GTPase by blocking the GAP activity of the trimeric TSC complex.^57^ A lipid modification of RHEB at the C-terminus known as farnesylation is essential for its lysosomal targeting and subsequent activation of mTORC1.^58^

GAPs act alongside GEFs, such as RAGULATOR for RAGs^59^ and ATP6AP1 and lysosomal EGFR for RHEB.^60,61^ Pentameric RAGULATOR is not only the GEF for RAGA, RAGB,^59^ and RAGC^62^ but also anchors RAGs to the lysosome.

### Amino acid sensing

Amino acid signaling to mTORC1 is arguably the most extensively studied regulatory network upstream of mTORC1 (Fig. 2). Although not fully understood, this signaling axis provides a compelling illustration of the fundamental principles by which the mTORC1 pathway senses and responds to environmental cues.

Multiple amino acid sensors signal amino acid availability to mTORC1. Thus, leucine sensors SESTRIN2^63^ and SAR1B,^64^ the arginine sensor CASTOR1/2,^65^ and the mitochondrion-localized threonine sensor TARS2^66^ detect free amino acids in the cytoplasm, whereas the lysosomal complex RAGULATOR^59^ senses the efflux of multiple amino acids from the lysosomal lumen (Fig. 2). SLC38A9, which is also located on the lysosome, can transport multiple amino acids from the lysosome lumen to the cytosol but has been demonstrated to function as a direct sensor only for arginine.^67–69^

Amino acid sensors function either through the GATOR2 complex (SESTRIN2,^63^ SAR1B,^64^ CASTOR1/2^65^) or the GATOR1 complex, with SAMTOR and PRMT1 acting as sensors for the metabolite of methionine, S-adenosylmethionine (SAM).^70–72^

The GATOR1 complex consists of three proteins: DEPDC5, NPRL2 and NPRL3. NPRL2 and NPRL3 form a heterodimer through interactions of their N-terminal longin domains (NLDs) and C-terminal regions, contributing to the stability of the complex.^73^ Arg78 of NPRL2 serves as the arginine finger responsible for the GAP activity of GATOR1.^74^ DEPDC5 contains domains with similarities to motifs found in membrane-associated proteins.^73,75^ The GATOR2 complex, which is composed of five proteins (WDR24, WDR59, MIOS, SEH1L and SEC13), is a coatomer-like structure.^76^ It shares structural features with other coating assemblies, such as the nuclear pore complex and coated vesicles, including shared components (SEH1 and SEC13).^75^ WDR24, WDR59, and MIOS also contain ring domains, which are critical for maintaining GATOR2 integrity. WDR24 exhibits intrinsic E3-ligase activity and mediates the ubiquitination of NPRL2 upon amino acid stimulation.^77^

In amino acid sufficiency, GATOR2 interacts with and inhibits GATOR1, thereby activating mTORC1.^47,76,78^ Amino acid deprivation leads to GATOR2 inhibition and GATOR1 activation, collectively resulting in mTORC1 inhibition. Thus, GATOR2 functions as an mTORC1 activator, whereas GATOR1 acts as an mTORC1 inhibitor.

In yeast, the GATOR2 and GATOR1 homologs, SEACAT and SEACIT complexes, respectively, can form a stable SEA complex.^79,80^ In mammalian cells, the GATOR1 and GATOR2 complexes exhibit weak direct interactions and rely on the tetrameric KICSTOR complex and the KICSTOR-interacting VWCE protein to anchor GATOR1 to lysosomes under conditions of amino acid or glucose deprivation.^81,82^ However, KICSTOR has only a limited role in positioning GATOR2 at the lysosomal surface. To coordinate the lysosomal localization of both GATOR complexes, cells depend on interleukin enhancer-binding factor 3 (ILF3).^83^

Leucine can be sensed via at least three sensors: SESTRIN2, SAR1B and leucyl-tRNA synthetase 1 (LARS1). SESTRIN2 and SAR1B interact with and inhibit GATOR2 in the absence of leucine, and this inhibition is lifted when leucine is present^64,84,85^ (Fig. 2). SESTRIN2 binds the WDR24/SEH1L subunits of GATOR2, whereas SAR1B directly interacts with the MIOS subunit. Interestingly, LARS1 is not only a canonical leucine sensor that catalyzes the ligation of leucine to its cognate tRNA. LARS1 also functions as a GAP for RAGD, thereby promoting mTORC1 activation.^86^

When arginine is low, CASTOR1/2 binds to and inhibits GATOR2, suppressing mTORC1.^65^ The three aromatic amino acids tyrosine, tryptophan, and phenylalanine (YWF) signal to mTORC1 via interactions between SESTRIN3 and GATOR2; however, whether SESTRIN3 is a direct sensor of YWF remains to be determined.^87^

Unlike other amino acid sensors that inhibit GATOR2 in the absence of their respective ligands, SAMTOR interacts with and activates GATOR1 in the absence of methionine or SAM^70,71^ (Fig. 2). Binding of SAMTOR to GATOR1 blocks the interaction of PRMT1 with GATOR1. When SAMTOR is bound to the SAM, it dissociates from GATOR1-KICSTOR. This allows SAM-bound PRMT1 to methylate NPRL2 at Arg78, thereby inhibiting the GAP activity of GATOR1 and promoting mTORC1 activation.^72^

GATOR2, GATOR1, and KICSTOR, together with RAGs and RAGULATOR, constitute key lysosome-localized signaling hub for nutrient sensing through mTORC1. Targeting RAGULATOR to the mitochondrial membrane is sufficient to relocate the RAGs and GATOR complexes to mitochondria, but this relocalization alone does not enable nutrient-dependent recruitment of mTORC1 to the organelle.^88^

Nevertheless, RAG-independent activation of mTORC1 by amino acids has been described in both yeast and human cells.^89–92^ For example, in RAGA/B-deficient cells, mTORC1 can still be stimulated by asparagine and glutamine via ARF1 GTPase.^89^ Amino acids released from lysosomal protein breakdown can activate mTORC1 through a mechanism that involves the homotypic fusion and protein sorting (HOPS) complex, independent of RAG GTPases.^93^ Additionally, mTORC1 activity is suppressed during amino acid deprivation because RAP1 GTPases promote lysosome repositioning to the perinuclear region and reduce lysosomal abundance.^94^ Finally, mTORC1 can also be activated at the Golgi membrane, where it is recruited by the RAB1A GTPase^91^ and subsequently activated by Golgi-localized RHEB.^95^ Non-farnesylated RHEB also regulates mTORC1 in the nucleus.^96^ Non-lysosomal mTORC1 appears to be under the control of exogenous amino acids and phosphorylates non-lysosomal, “canonical” substrates S6K1 and 4E-BP1.^97^ At the same time, a fraction of mTORC1 localizes to the lysosome, which locally supplies endogenous amino acids through basal lysosomal proteolysis. This lysosomal, RAG-dependent mTORC1 acts on the “noncanonical substrates” TFEB and TFE3.^97^

While core components of the mTORC1 nutrient-sensing pathway (RAGs, RAGULATOR, and GATOR1/GATOR2 complexes) are conserved across metazoans,^75^ many direct amino acid sensors are not.^28^ Even conserved proteins may function differently across species.^98^ For example, *D. melanogaster* lacks arginine sensors but retains SESTRIN and SAMTOR. Nevertheless, *Drosophila* Sestrin binds leucine with significantly lower affinity than its human homolog does. Furthermore, SAM is not sensed by dSamtor but by the Umet protein, which binds to GATOR2,^99^ demonstrating that flies and vertebrates independently evolved unrelated, mechanistically distinct sensors that converge upon the same metabolite. In addition, GATOR2 in *Drosophila* has a TORC1-independent role in the regulation of the lysosomal autophagic endomembrane system.^100^ *C. elegans* has SESTRIN and SLC38A9 homologs but no clear SAMTOR counterpart. This patchy distribution may reflect species-specific metabolic requirements. In support of this, *S. cerevisiae* seems to regulate TORC1 on the basis of overall nitrogen and carbon availability rather than individual amino acid levels. Indeed, *S. cerevisiae*, which can synthesize all amino acids de novo, lacks specialized amino acid sensors but maintains the general amino acid sensors Gcn2, FLCN/FNIP (Lst4/Lst7) and GATOR1/2 (SEACIT/SEACAT), RAGs (Gtr1/Gtr2) and RAGULATOR (Ego).^101^ In addition, *S. cerevisiae* lacks the genes encoding components of the TSC complex, and its RHEB homolog, Rhb1, does not participate in TORC1 regulation. Even more intriguing, plants do not seem to have specific amino acid sensors, and GATOR/RAG/RAGULATOR or their equivalents have not yet been identified in plants so far. These observations suggest that nutrient sensors may have evolved in response to organism-specific nutritional environments, highlighting both the evolutionary conservation and divergence of the mechanisms that control TORC1 signaling in different organisms.

Together, these findings underscore the versatility and complexity of mTORC1 regulation across multiple cellular compartments. While the lysosome remains the central platform for amino acid and nutrient sensing via the RAG–RAGULATOR–GATOR–KICSTOR axis, alternative mechanisms allow mTORC1 to integrate diverse metabolic cues from other organelles, such as mitochondria, the Golgi, and even the nucleus. These parallel and sometimes redundant pathways highlight the robustness of mTORC1 as a metabolic rheostat capable of maintaining cellular homeostasis under varying nutrient conditions.

### Growth factor sensing

Growth factors are key extracellular signals that regulate mTORC1 (Fig. 2). Receptor tyrosine kinases (RTKs), insulin and insulin-like growth factor 1 (IGF-1), WNT and tumor necrosis factor (TNF), inflammatory cytokines, and RAS activate multiple signaling pathways that converge on a major upstream inhibitor of mTORC1, the TSC complex, which is composed of TSC1, TSC2, and TBC1D7.^102^ TSC is a GAP that converts RHEB from its GTP-bound active form to a GDP-bound inactive state.^103,104^

Phosphorylation of TSC components by various kinases leads to TSC complex inhibition and subsequent activation of mTORC1 (Fig. 2). For example, insulin and IGF1 stimulation promote AKT-mediated phosphorylation of TSC2 at multiple sites.^105^ As a result, the GAP function of the TSC complex toward RHEB is diminished, leading to an accumulation of active GTP-bound RHEB, which directly stimulates mTORC1 at the lysosomal surface.^103,106^

Upon insulin stimulation, mTORC1 activates S6K1, which in turn phosphorylates insulin receptor substrate 1 (IRS1) on multiple serine residues, which marks IRS1 for degradation or functional inhibition, thereby dampening further PI3K–AKT signaling upstream of mTORC1.^107,108^ This feedback mechanism acts as a regulatory brake, preventing prolonged or excessive activation of mTORC1 in response to sustained insulin signaling.

In RTK-mediated RAS signaling, the mTORC1 pathway is activated via MAPK/ERK and its effector p90RSK through the RTK → RAS → MAPK/ERK → p90RSK cascade, further resulting in the phosphorylation of TSC2.^109^ WNT and TNFα activate mTORC1 by repressing TSC1^110,111^ (Fig. 2).

Beyond TSC and RHEB regulation, growth factors can stimulate the activation of mTORC1 through alternative routes (Fig. 2). For example, insulin promotes the phosphorylation of PRAS40 by AKT, which is associated with the sequestration of PRAS40 by the cytosolic anchor protein 14-3-3. This sequestration effectively removes the inhibitory effects of PRAS40 on mTORC1, thereby restoring the kinase activity of mTORC1.^112,113^ Finally, the GATOR1 complex appears to be necessary for rapid and robust mTORC1 inhibition following growth factor withdrawal.^114^

### Glucose sensing

Glucose is a major energy source for cells, supplying ATP through glycolysis and oxidative phosphorylation. Under low-glucose conditions, ATP levels drop and the cellular energy status shifts, leading to an increased AMP/ATP ratio. This triggers the activation of AMP-activated protein kinase (AMPK), a key energy sensor that phosphorylates and stimulates the TSC complex, leading to the inhibition of RHEB and subsequent mTORC1 suppression (Fig. 2). AMPK can also phosphorylate RAPTOR, impairing its interaction with other mTORC1 components, thus dissociating mTORC1 from its activators.^115,116^ In addition, under glucose deprivation, AMPK directly phosphorylates WDR24, subsequently disrupting the integrity of the GATOR2 complex and leading to mTORC1 inactivation.^117^ Glucose starvation also induces the phosphorylation of the leucine sensor LARS1 by ULK1 at residues important for Leu binding, thereby reducing the affinity of LARS1 for leucine.^118^ This leads to mTORC1 suppression, the inhibition of protein synthesis and the activation of autophagy, highlighting a point of convergence between glucose and amino acid sensing in the regulation of mTORC1.

When glucose is abundant, AMPK activity is reduced, relieving the inhibition of mTORC1 and enabling its activation to support cell growth and proliferation.^119^ Metabolic products of glucose, such as DHAP or FBP, can activate mTORC1 either via the GATOR2 complex^120^ or via RAGULATOR^,^^121^ respectively. In addition, galectin-3, a lipopolysaccharide sensor, promotes the interaction between RAGA, RAGC and RAGULATOR, activating mTORC1.^122^

### Lipid sensing

While amino acids are the primary nutrients sensed at the lysosome, recent studies have demonstrated that lipids can also be sensed by this organelle (Fig. 2). Thus, the GPCR-like protein LYCHOS detects cholesterol sufficiency and activates mTORC1 in a RAG-dependent manner by sequestering GATOR1.^123^ Additionally, the arginine sensor SLC38A9 signals cholesterol independently of its arginine-sensing function through a conserved cholesterol-interacting motif, promoting the SLC38A9-RAGA interaction and mTORC1 activation.^124^ In contrast, the Niemann‒Pick C1 (NPC1) protein, which regulates cholesterol export from the lysosome, binds to SLC38A9 and inhibits mTORC1 signaling through its sterol transport function.^124^

Another example concerns omega-6 linoleic acid (ω-6LA), a polyunsaturated essential fatty acid that must be obtained through dietary sources. ω-6 LA is a direct activator of mTORC1.^125^ ω-6 LA action on mTORC1 depends on the lipid chaperone fatty acid–binding protein 5 (FABP5). In the presence of ω-6LA, FABP5 binds to RAPTOR, enhancing mTORC1 complex assembly and increasing the phosphorylation of S6K1 and 4E-BP1. ω-6 LA activates mTORC1 even in the absence of amino acids and growth factors, which implies that lipids can provide divergent regulatory signals for mTORC1 activation. Phosphatidic acid (PA), oleic acid, and palmitate also activate mTORC1^126^ but do not require FABP5. Instead, mTORC1 and mTORC2 are activated in response to exogenously supplied fatty acids via the de novo synthesis of PA, a central metabolite for membrane phospholipid biosynthesis.

### DNA damage response

DNA damage, which can be induced by ultraviolet (UV) light, ionizing radiation, and reactive oxygen and nitrogen species, suppresses mTORC1^127^ (Fig. 2). To counteract the threats posed by DNA damage, cells have evolved the DNA damage response (DDR), which detects DNA lesions, signals their presence, and facilitates their repair. The crosstalk between the DDR and the mTORC1 pathway constitutes a critical mechanism by which cells handle genotoxic and metabolic stresses.^128^

Several molecules signal DNA damage in mammalian cells, including ataxia telangiectasia mutated (ATM), ataxia telangiectasia and Rad3 related (ATR) and DNA protein kinase (DNA-PK), which, like mTOR, are members of the PIKK kinase family^128^ (Fig. 2). DNA damage activates ATM, which promotes the phosphorylation of CHK2 and p53, which are required for G1/S arrest initiation and maintenance.^129^ In response to DNA damage, p53 is activated by ATM/ATR kinases through phosphorylation and translocates to the nucleus, where it regulates multiple targets, including the AMPK regulatory subunit β and TSC2.^130,131^ Under oxidative stress or hypoxic conditions, ATM activation suppresses mTORC1 signaling by stimulating the AMPK pathway, which in turn enhances TSC activity.^128,132^ ROS-induced DNA damage also stimulates poly(ADP‒ribose) polymerase (PARP), thereby activating AMPK and inhibiting mTORC1 through TSC.^133,134^ Inhibition of mTORC1 by rapamycin disrupts a negative feedback loop mediated by S6K1, which normally suppresses PI3K/AKT signaling through the inhibitory phosphorylation of IRS1. Loss of this feedback enhances IRS1 activity, leading to increased PI3K signaling and subsequent AKT phosphorylation. Under conditions of DNA damage or stress, DNA-PK can further contribute to AKT activation.^135^ AKT then further activates mTORC1 by phosphorylating TSC2 and PRAS40.^136,137^ This compensatory activation of AKT limits the efficacy of mTORC1-targeted therapies and underscores the rationale for using dual PI3K/mTOR or mTORC1/mTORC2 inhibitors to prevent feedback-driven survival signaling.

In summary, the PI3K‒AKT pathway, along with p53 signaling, connects the DDR and mTORC1. These intricate regulatory mechanisms highlight the multifaceted nature of mTORC1 as a critical sensor and regulator of cellular stress responses.

## Upstream regulation of mTORC2

mTORC2 is mainly controlled by insulin and growth factors (Fig. 2). Upon insulin or growth factor stimulation, increased levels of PtdIns(3,4,5)P₃ compete with the mTOR kinase domain for binding to the pleckstrin homology (PH) domain of SIN1. This interaction relieves the SIN1–PH–mediated autoinhibition of mTORC2 activity. Additionally, the binding of PtdIns(3,4,5)P₃ to SIN1-PH facilitates the recruitment of the mTORC2 complex to the plasma membrane, where it can phosphorylate membrane-associated AKT.^138,139^ In addition, insulin induces mTORC2 activation by assembling a supercomplex with the GTPases KRAS4B and RHOA, termed KARATE, which controls the translocation of the insulin-responsive glucose transporter GLUT4 to the plasma membrane during insulin stimulation in adipocytes.^140^ Importantly, increased growth factor-mediated PI3K signaling also enhances mTORC2 association with ribosomes, which promotes cotranslational phosphorylation of the main mTORC2 substrate, AKT.^141,142^

mTORC2 activation can also be triggered by G protein-coupled receptors (GPCRs), which increase intracellular cAMP levels and facilitate glucose uptake through mTORC2-mediated pathways.^143,144^ Additionally, mTORC2 appears to integrate signals from both the glucagon receptor (GCGR) of a GPCR type and the insulin receptor, contributing to improved insulin sensitivity. While prolonged GCGR activation is typically associated with elevated blood glucose levels and impaired glucose tolerance, short-term stimulation enhances glucose utilization and insulin responsiveness through hepatic RICTOR/mTORC2 and AKT signaling.^145^ These findings suggest that mTORC2 plays a supportive role in glucose metabolism upon GPCR activation and that this role may be functionally distinct from classical insulin/PI3K/AKT signaling.

Although mTORC1 is the primary complex that responds to amino acid availability, reintroducing amino acids to cells deprived of both serum and amino acids increases the phosphorylation of AKT by mTORC2^146^ (Fig. 2). Amino acid mixtures with naturally alkaline pH, but not those adjusted to physiological pH, also stimulate AKT phosphorylation,^147^ suggesting that mTORC2 may respond to intracellular pH changes rather than directly to amino acid concentrations. For example, elevated intracellular alkalinity after treatment with ammonium chloride or ammonium hydroxide also stimulates mTORC2 signaling.^148^ In this context, calcium mobilization and subsequent activation of CaMKKβ activate AMPK, which might modulate mTORC2 signaling, possibly through direct phosphorylation of mTOR.^149^ In addition, cystine activates mTORC2 by promoting SIN1 phosphorylation.^150^

Finally, mTORC2 activity can also be modulated by glucose. Glucose starvation directly activates mTORC2 via AMPK phosphorylation of mTOR and RICTOR,^149^ leading to increased AKT phosphorylation.^151^ On the other hand, glucose can activate mTORC2 through acetylation of RICTOR.^152^

## Downstream of mTORC1

### Posttranscriptional regulation

mTORC1 impacts mRNA maturation by influencing alternative splicing and m^6^A RNA modification.^153–157^ S6K1 phosphorylates SRPK2, enabling the nuclear translocation of this kinase, which in turn phosphorylates serine/arginine-rich (SR) splicing factors. Nuclear SRPK2 enhances interactions between the splicing factors SRSF1 and U1-70K and supports the formation of a complex involving SREBP1, FAM120A, and RNA Pol II, thereby coordinating nutrient-dependent transcription and splicing of lipogenic genes.^155^ In addition, S6K1 phosphorylates SKAR at exon junction complexes, increasing the efficiency of translation elongation of spliced transcripts.^158^ When mTORC1 is inhibited, nuclear SRPK2 levels decrease, causing intron retention and nonsense-mediated decay of target mRNAs.^155^

In *C. elegans*, mTORC1 regulates mRNA splicing and the production of protein-coding mRNA isoforms by increasing the expression and activity of the SR protein RSP-6 (SRSF3/7 in mammals) and other splicing factors, independent of S6K-1. Mutations in other *C. elegans* splicing factors, RNP-6 (PUF60 in mammals) and RBM39, which are involved in 3′ splice site recognition, are associated with changes in lifespan through mTOR signaling.^159^

mTORC1 also regulates m^6^A mRNA methylation, which is critical for cell growth and cancer progression.^160^ Via the S6K1–eIF4A/B axis, mTORC1 enhances the translation of WTAP, a component of the METTL3–METTL14–WTAP m^6^A writer complex, particularly owing to WTAP’s structured 5′ UTR.^154^ m^6^A modifications promote mRNA destabilization of MXD2 transcript, thereby increasing c-Myc activity.^154,156^ mTORC1 also increases c-Myc protein translation by stimulating eIF4A helicase activity.^161^ Additionally, mTORC1 promotes the degradation of c-Myc antagonists, including MAD1 (via phosphorylation by S6K1) and MXD2 (via m^6^A modification), further enhancing c-Myc transcriptional activity.^154,162^

### Protein synthesis

mTORC1 controls protein synthesis through the phosphorylation of eIF4E binding protein and S6K1 kinase.^41^ mTORC1 phosphorylates S6K1 at Thr389, activating it, while phosphorylation of 4E-BP1 at multiple sites inhibits its repressor function (Fig. 2, Table 1). Ribosomal protein S6 is the best-known substrate of S6K1, which also phosphorylates many other proteins, including the translation factors eIF4B, eEF2K, and cap-binding protein 80 kDa (CBP80).^163^ S6K1 substrates promote mRNA translation initiation through interaction with eIF4B, which is crucial for the 5′ cap binding of the eIF4F complex. 4E-BP1 blocks translation by binding to eIF4E, thereby preventing the formation of the eIF4F complex. mTORC1 phosphorylates 4E-BP1 at several sites, causing it to release eIF4E, which then permits 5’ cap-dependent mRNA translation.^164^

mTORC1 regulates the translation of 5′-terminal oligopyrimidine (TOP) motif-containing mRNAs, which encode components of the protein synthesis machinery. LARP1, a key repressor of TOP mRNA translation, binds both the 5′ cap and adjacent TOP motif to block eIF4F complex assembly.^165,166^ Upon mTORC1 activation, LARP1 is phosphorylated, relieving this repression^167,168^ and facilitating the translation of TOP mRNAs via interaction with RAPTOR.^166^ mTORC1 also modulates translation through 3′ UTR shortening, which enhances polysome formation and protein output, especially in genes related to protein folding and degradation.^169,170^

mTORC1 also controls translation by regulating ribosome biogenesis, promoting rRNA transcription of RNA Pol I via TIF1A^171^ and translation of ribosomal proteins, whose mRNAs contain TOP motifs.^165,172^ mTORC1 also phosphorylates and inhibits MAF1, a negative regulator of Pol III, enhancing tRNA and 5S rRNA synthesis, which is critical for ribosome function.^173–175^ Finally, mTORC1 controls ribosome concentration, affecting phase separation in the cytosol,^176^ providing new insights into how cells balance efficient biosynthesis with the risk of molecular overcrowding.

### Nucleotide synthesis

To support DNA replication and RNA production in dividing cells, mTORC1 regulates the availability of one-carbon units necessary for nucleotide biosynthesis. mTORC1 activates the transcription factor ATF4, which in turn upregulates MTHFD2, a mitochondrial enzyme involved in the tetrahydrofolate cycle that contributes to de novo purine synthesis.^177^ Additionally, through S6K1, mTORC1 facilitates the phosphorylation and activation of the CAD complex, which is the rate-limiting step in pyrimidine biosynthesis.^178,179^ In cells with hyperactive mTORC1 signaling, disrupting guanylate nucleotide synthesis can lead to DNA damage, as limited nucleotide resources are preferentially allocated to rRNA synthesis to support elevated ribosome production and protein synthesis.^180^

### Lipid synthesis

mTORC1 plays a significant role in regulating de novo lipid synthesis through the activation of the sterol regulatory element-binding protein (SREBP) transcription factor, which modulates the expression of metabolic genes involved in fatty acid and cholesterol biosynthesis.^181^ SREBP is active when sterol levels are low. mTORC1 can activate SREBP through S6K1, which phosphorylates and activates SREBP cleavage-activating protein (SCAP). This leads to the translocation of the active form of SREBP to the nucleus and the upregulation of genes involved in lipid synthesis. mTORC1 also phosphorylates and inactivates LIPIN-1, which is a negative regulator of SREBP activity.^182^ When mTORC1 is suppressed, LIPIN-1 represses SREBP. mTORC1 activation leads to LIPIN-1 phosphorylation and inactivation, followed by derepression of SREBP and upregulation of lipid biosynthesis.

### Autophagy

mTORC1 is a negative regulator of autophagy, a catabolic process by which the cytosol and organelles are sequestered within double membrane-bound vesicles that deliver their contents to the lysosome for degradation and recycling^183^ (Figs. 2, 7). mTORC1 is essential not only for general autophagy regulation but also for specialized forms such as mitophagy, ribophagy, and lipophagy, where it modulates the selective degradation of mitochondria, ribosomes, and lipid droplets, respectively.

mTORC1 is active and autophagy is suppressed under optimal growth conditions, e.g., a sufficient number of amino acids and glucose. To inhibit autophagy initiation, mTORC1 associates with a ULK protein complex composed of ULK1, ULK2, ATG13, ATG101 and the scaffold protein FIP200. RAPTOR interaction with ULK1/2 brings the ULK complex into proximity with mTORC1, allowing mTORC1 to phosphorylate ULK1/2 and ATG13. This phosphorylation prevents kinase activity of the ULK complex, thereby suppressing autophagosome formation.^184^

Under nutrient deprivation conditions or during DNA damage, mTORC1 is suppressed and detaches from the ULK1 complex. This dissociation results in the dephosphorylation of ULK1 and ATG13, which activates the kinase function of ULK1. ULK1 then phosphorylates both ATG13 and FIP200, triggering the recruitment of the BECLIN-1–VPS34 complex to the pre-autophagosomal membrane and facilitating autophagosome formation.

Another mechanism that involves mTORC1 during autophagy is modulated by the autophagy receptor Sequestosome 1 (SQSTM1)/p62, which interacts with RAPTOR and RAGC, thereby recruiting and activating mTORC1 on the lysosomal surface.^185^ Recently, the WD-repeat domain protein MORG1 was shown to play a role in maintaining basal autophagy by suppressing mTORC1 signaling via interference with RAG GTPases.^186^ Specifically, MORG1 associates with the active RAG GTPase complex, thereby preventing the RAG-dependent recruitment of mTORC1 to lysosomes. Loss of MORG1 leads to increased mTORC1 activity and reduced autophagic flux. Although p62/SQSTM1 can bind MORG1, the latter is not degraded through autophagy. Upon amino acid refeeding after deprivation, p62/SQSTM1 binds to MORG1, which disrupts the ability of MORG1 to inhibit RAGs, allowing mTORC1 activation.

Autophagy can also be induced when cellular AMP levels are high, activating AMPK and resulting in mTORC1 suppression (Fig. 7). In addition, during glucose starvation, AMPK phosphorylates ULK1 at different sets of serines than does mTORC1, which activates ULK1 and initiates autophagosome formation.^119^ Moreover, the phosphorylation of ULK1 at Ser555 by AMPK is required for proper mitochondrial homeostasis. Loss of this phosphorylation leads to the accumulation of mitochondria with aberrant morphology, which cannot be eliminated by mitophagy.^187^

mTORC1 also regulates autophagy at the transcriptional level by modulating the localization of TFEB, which regulates the expression of autophagy and lysosomal genes. mTORC1 phosphorylates TFEB, thereby inhibiting TFEB activity when nutrients are present.^43,188^ Upon mTORC1 inhibition, TFEB becomes dephosphorylated and translocates to the nucleus, where it activates the transcription of genes involved in autophagy and lysosomal biogenesis.

### Ubiquitin‒proteasome system

The ubiquitin‒proteasome system (UPS) is responsible for targeted protein degradation. The activation of mTORC1, either through the loss of TSC1 or TSC2 or in response to growth factors, promotes the expression of proteasome subunits by increasing the activity of the transcription factor nuclear respiratory factor 1 (NRF1). NRF1 drives proteasome gene transcription in response to proteotoxic stress,^189^ demonstrating that mTORC1 signaling supports proteasome biogenesis during periods of high anabolic demand. Additionally, mTORC1 can influence substrate degradation by regulating the phosphorylation status of UPS-targeted proteins. For example, phosphorylation of LIPIN1 by mTORC1 allows its recognition by the E3 ligase β-TRCP1, leading to its ubiquitination and proteasomal degradation.^190^ Paradoxically, other studies have demonstrated that mTOR inhibition, rather than activation, can induce overall protein degradation via both the UPS and autophagy.^191^ If mTORC1 activation promotes proteasome biogenesis, why would mTORC1 inhibition also increase degradation?

These discrepancies might be explained and reconciled by recent studies, which demonstrated that after amino acid deprivation, the proteasome moves from its predominant location in the nucleus to the cytoplasm, promoting protein degradation in the cytoplasm and thereby supplying the amino acids necessary for cell survival.^87,192^ This relocation is mediated via the mTORC1 pathway and the lack of three aromatic amino acids, tyrosine, tryptophan, and phenylalanine (YWF), which transmit their signals upstream of mTORC1 by disrupting SESTRIN3 binding to the GATOR2 complex (Fig. 2). Active mTORC1 then phosphorylates p62 and TFEB, influencing proteasomal function and autophagy. When mTORC1 is activated by YWF and the proteasome remains confined to the nucleus, this proteolytic response is suppressed, resulting in cell death. Interestingly, the modulation of the signaling cascade governed by YWF is also applicable to non-starved cells by using a relatively high concentration of YWF to achieve a surplus relative to all other amino acids.^192^ Therefore, proteasome localization, which is regulated by mTORC1 and YWF signaling, could explain why both the activation and inhibition of mTORC1 can lead to increased protein degradation under different conditions and via different mechanisms.

## Downstream of mTORC2

AKT, a key substrate of mTORC2, regulates glucose metabolism by inhibiting glycogen synthase kinase 3β (GSK3β) and the Forkhead Box O (FOXO) family of transcription factors.^193^ mTORC2 phosphorylates AKT at Ser473 in response to the presence of growth factors, which enhances PI3K activation.^194,195^ mTORC2 also mediates Thr450 phosphorylation of AKT, which is independent of PI3K, occurs during AKT translation and contributes to AKT stability.^141,196^ Phosphorylation at Thr450 is dependent on glucose due to the sensitivity of mTORC2 to ATP levels.^197^ AKT also mediates the connection between mTORC2 and mTORC1 by phosphorylating and inhibiting TSC2. Furthermore, mTORC2 contributes to cytoskeleton rearrangements and cell mobility through PKC, a key regulator of the cytoskeletal network.^198^ mTORC2 also activates SGK1,^199^ which augments the expression and activity of Na^+^/K^+^ ATPase (sodium-potassium pump) and glucose and amino acid carriers in the plasma membrane and promotes the upregulation of Na^+^, Ca^2+^, K^+^, and Cl^−^ channels.

Thus, through its key effectors, mTORC2 coordinates glucose metabolism, cytoskeletal dynamics, membrane transport, and ion channel regulation.

## Regulation of mitochondrial homeostasis by mTORC1 and mTORC2

Both mTORC1 and mTORC2 are essential for mitochondrial biogenesis, dynamics, the phosphorylation of mitochondrial proteins, mitochondrial genome repair, the cellular response to mitochondrial ROS, and the regulation of mitophagy.^200,201^

mTORC1 (more so than mTORC2) plays a critical role in maintaining mitochondrial mass by balancing two opposing processes: promoting mitochondrial biogenesis and facilitating degradation. Mitochondrial biogenesis is enhanced by mTOR-mediated activity in the transcription and translation of nuclear-encoded mitochondrial RNAs and proteins as well as fusion and fission of preexisting organelles. On the other hand, mTOR also participates in mitochondrial quality control and coordinates the degradation of defective organelles via autophagy and mitophagy.

Mitochondria cannot be synthesized de novo. New organelles are formed from preexisting ones through fusion and fission.^202,203^ Mitochondrial biogenesis also requires the replication, transcription and translation of mitochondrial DNA (mtDNA)-encoding genes, as well as the import of lipids and nuclear-encoded proteins.^204^ Because human mtDNA contains only 37 genes, the majority of proteins essential for mitochondrial function are nuclear encoded. After transcription and translation, these proteins are targeted to the mitochondria and imported into various subcompartments.^205^ Mitonuclear feedback is achieved through bidirectional communication: anterograde signaling, where information flows from the nucleus to the mitochondria, and retrograde signaling (RTG), where the mitochondria transmit signals to the nucleus in response to mitochondrial dysfunction.^206,207^ mTORC1 plays a central role in mitonuclear communication by modulating various extra- and intracellular stimuli, that regulate mitochondrial biogenesis and function.

### mTORC1 in mitochondrial biogenesis

**Fig. 3:**
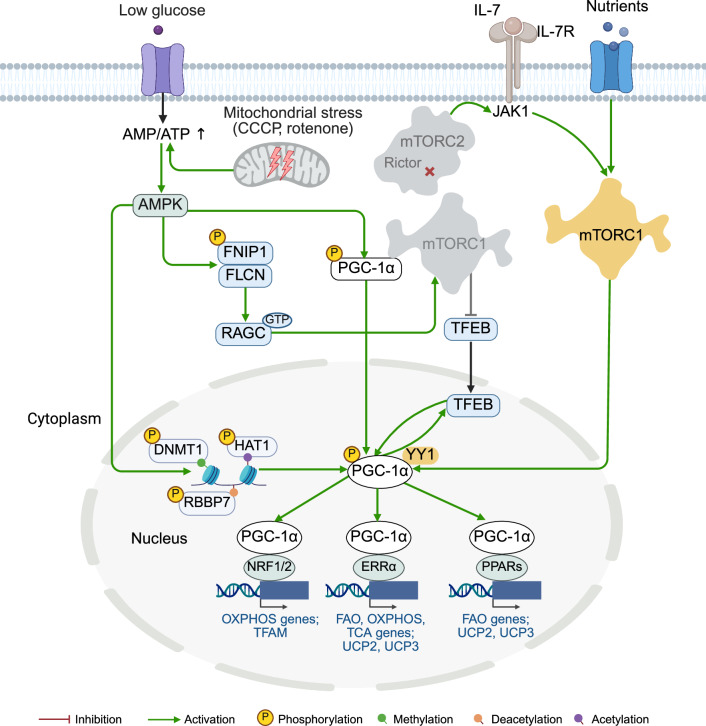
Regulation of mitochondrial biogenesis by mTOR at the transcriptional level. mTORC1 regulates mitochondrial gene transcription by activating PGC-1α through multiple mechanisms: (i) via interaction with the transcription factor YY1; (ii) via TFEB dephosphorylation and nuclear translocation; and (iii) through AMPK-mediated phosphorylation of PGC-1α. Mitochondrial damage (for example, caused by CCCP or rotenone treatment) activates AMPK, which in turn phosphorylates FNIP1 and suppresses FLCN–FNIP1 GAP activity. This maintains RAGC in its GTP-bound form and promotes TFEB nuclear translocation. Nuclear TFEB induces PGC-1α, which in turn enhances TFEB expression through a positive feedback loop. AMPK also phosphorylates three epigenetic factors: DNA methyltransferase 1 (DNMT1), retinoblastoma binding protein 7 (RBBP7) and histone acetyltransferase 1 (HAT1). This leads to nucleosome remodeling and activation of the promoters of PGC-1α, TFAM, NRF1 and NRF2, as well as uncoupling of proteins 2 and 3 (UCP2 and UCP3). PGC-1α couples with distinct transcription factors to regulate mitochondrial metabolism: with NRF1/2 to induce OXPHOS genes and TFAM; with estrogen-related receptor alpha (ERRα) to activate fatty acid oxidation (FAO), the TCA cycle, OXPHOS genes, and the UCP2 and UCP3 genes; and with peroxisome proliferator–activated receptors (PPARs) to drive FAO, UCP2 and UCP3 genes. mTORC2 does not directly control mitochondrial transcription; it modulates upstream IL-7R/JAK1 signaling to influence mTORC1 activity

mTORC1 regulates the transcription of mitochondrial genes by acting on the master regulator of mitochondrial biogenesis, peroxisome proliferator-activated receptor gamma coactivator 1-alpha (PGC-1α)^208^ (Fig. 3). PGC-1α is a transcriptional coactivator that interacts with several transcription factors to control the transcription of more than 150 genes encoding mitochondrial proteins involved in fatty acid oxidation, the tricarboxylic acid (TCA) cycle, oxidative phosphorylation (OXPHOS), the mitochondrial ribosomal machinery and membrane transport.

The regulation of PGC-1α by mTORC1 occurs through multiple mechanisms: by interacting with the transcription factor Yin-Yang 1 (YY1), through the upstream energy sensor AMPK, or via the mTORC1 downstream effector TFEB.

To interact with PGC-1α via YY1, both the mTOR and the RAPTOR components of mTORC1 bind directly to different sites of YY1.^208^ This represents one of the rare examples of mTORC1’s nuclear activity. Treatment with rapamycin disrupts the interaction between YY1 and PGC-1α, resulting in decreased expression of mitochondrial genes.

In contrast to mTORC1, AMPK directly interacts with and phosphorylates PGC-1α, leading to the autoactivation of the PGC-1α promoter and the induction of several mitochondrial genes^209^ and epigenetic factors^210^ (Fig. 3). AMPK-mediated phosphorylation of epigenetic factors not only enhances mitochondrial biogenesis but also increases the membrane potential.

AMPK is also important for the activation of TFEB, which plays an essential role in mitochondrial biogenesis by upregulating PGC-1α expression (Fig. 3). Mitochondrial stress, induced by the application of electron transport chain inhibitors, triggers the activation of AMPK, which in turn phosphorylates FNIP1. This phosphorylation inhibits FLCN-FNIP1 GAP activity, leading to the accumulation of the inactive RAGC-GTP-bound form, pushing TFEB off the lysosome, and facilitating its nuclear translocation. Once in the nucleus, TFEB enhances the transcription of PGC-1α.^211^ PGC-1α, in turn, interacts with the promoter region of the TFEB-encoding gene, creating a feedback loop that increases TFEB expression and activity. This loop supports mitochondrial biogenesis, enabling adaptation to genotoxic and metabolic stressors.^212^ Additionally, TFEB can drive mitochondrial biogenesis independently of PGC-1α by upregulating NRF1/2 and transcription factor A (TFAM), leading to increased mtDNA synthesis.^213^

mTORC1 stimulates the translation of nuclear-encoded mitochondrial proteins and promotes the transcription of mRNAs related to mitochondrial functions through the phosphorylation of 4E-BP1 and S6K1.^214,215^ On the other hand, mitochondrial activity and its tethering with the endoplasmic reticulum (ER) are linked to the compartmentalization of translation initiation factor eIF4E to polysomes attached to the rough ER (rER).^216^ In mammalian cells with functional mitochondria and active mTORC1, 4E-BP1 is localized on polysomes attached to the rER, leading to the dissociation of eIF4E and its recruitment to the active translation initiation complex eIF4F, enabling protein synthesis. Impaired mitochondrial fusion disrupts the tethering of mitochondria to the rER, reducing eIF4E targeting to the rER. mTORC1 inhibition, which results in decreased 4E-BP1 phosphorylation, reduces the intracellular pool of free eIF4E, promoting its defective compartmentalization to polysomes attached to the rER membrane, which causes retarded translation.

mTORC2 does not directly regulate the transcription of mitochondrial genes but can influence this process indirectly through mTORC1 (Fig. 3). For example, the deletion of *Rictor* leads to an increase in the mitochondrial DNA content and overall mitochondrial mass in immature myeloid dendritic cells.^217^ This effect is attributed to compensatory mTORC1 activity, characterized by increased expression of several upstream regulators of mTORC1 (e.g., IL-7R and JAK1) and activation of its downstream targets S6K1 and 4E-BP1.

Overall, transcriptional and translational regulation act as a feedforward mechanism, where the translation of nuclear-encoded mitochondrial mRNAs is controlled by the mTORC1 pathway to induce ATP production and ensure adequate energy availability for protein synthesis.

### mTORC1 and mTORC2 in the regulation of mitochondrial dynamics

Mitochondria are highly dynamic and interconnected in a complex network.^218^ Fusion and fission allow mitochondria to share membranes, metabolites, solutes, and proteins and maintain an electrochemical gradient.^204^ Nutrient availability drives mitochondrial fragmentation, whereas acute starvation results in the formation of an elongated, tubular-like mitochondrial network.^219,220^

The fission‒fusion cycle helps repair, regenerate, and reintroduce healthy daughter mitochondria into the mitochondrial pool. This process is essential for maintaining a healthy population of mitochondria. Moreover, mitochondrial fission isolates damaged or dysfunctional mitochondria from the healthy network, followed by their selective elimination through mitophagy.

**Fig. 4:**
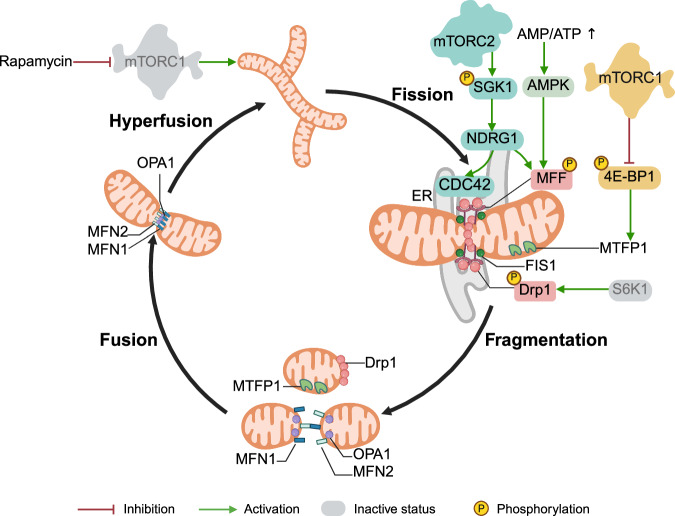
Role of mTORC1 and mTORC2 in controlling mitochondrial dynamics. mTORC1 phosphorylates 4E-BP1, inactivating its repressor function and relieving cap-dependent blockade of MTFP1 mRNA translation, facilitating mitochondrial fission. Deletion of S6K1 in mouse embryonic fibroblasts enhances mitochondrial fission by increasing total DRP1 and its Ser616-phosphorylated form (a modification mediated by other kinases, not by S6K1). During extended nutrient deprivation, mTORC2 phosphorylates and activates SGK1, which in turn phosphorylates NDRG1 to promote mitochondrial fission. Fusion begins with the docking of two MFN1 molecules in trans, followed by conformational changes and GTP hydrolysis of MFN1, leading to OMM fusion. OPA1, in coordination with cardiolipins, is responsible for IMM fusion. mTORC1 inhibition in vitro (by rapamycin) or during acute starvation promotes mitochondrial fusion

Fission occurs near the smooth ER, which wraps around mitochondria. Fission is regulated by the cytosolic GTPase dynamin-related protein 1 (DRP1). Upon nutrient sufficiency, DRP1 is recruited to the outer mitochondrial membrane (OMM), where it assembles into a multimeric ring complex that compresses the OMM to trigger division (Fig. 4). DRP1 interacts with OMM-residing proteins, fission 1 protein (FIS1) and mitochondrial fission factor (MFF), which function as DRP1 receptors.^221,222^ Fission is also regulated by posttranslational modifications of DRP1, especially its phosphorylation by cyclic AMP-dependent protein kinase (PKA) at Ser637 and cyclin-dependent kinase (CDK1) at Ser616.^219^

Mitochondrial fusion is controlled by three GTPases from the dynamin superfamily: homologous mitofusin 1 and 2 (MFN1 and MFN2), which reside at the OMM, and inner membrane (IMM)-specific optic atrophy 1 protein (OPA1) (Fig. 4).^219^

Together with its upstream regulators and downstream effectors, mTORC1 plays a significant role in regulating mitochondrial fission and fusion (Fig. 4). In vitro studies have shown that active mTORC1 promotes mitochondrial fission, whereas mTORC1 inhibition during amino acid starvation shifts the balance toward mitochondrial fusion.^219^ Depletion of serum or glucose has the opposite effect, inducing mitochondrial fragmentation. Conversely, rapamycin treatment reduces the expression of FIS1 and DRP1,^223^ as well as DRP1 phosphorylation.^224^ This treatment can also increase ER‒mitochondria coupling, facilitating mitochondrial fusion.^225^

In contrast to in vitro findings, studies in animals (mice^226,227^ and *C. elegans*^228^) have shown that fasting leads to mitochondrial fragmentation. This discrepancy may be explained not only by variations between cultured cells and living organisms but also by the time during which starvation is conducted in cell culture (1–2 h) and in animals (24–48 h). During acute starvation, mitochondria might elongate to cope with stress and promote cell survival after nutrient depletion. Long-term fasting induces fission to facilitate the clearance of damaged mitochondria via mitophagy and increases the intercellular supply of nutrients necessary to sustain cellular functions. Importantly, mTORC1 activity also varies depending on the duration of starvation: it is repressed during acute starvation, whereas prolonged nutrient deprivation leads to robust reactivation of mTORC1.^229,230^

Deletion or overexpression of upstream mTORC1 regulators influences mitochondrial dynamics by promoting fission or fusion, depending on whether mTORC1 is activated or repressed. For example, RHEB knockout inhibits mTORC1, resulting in mitochondrial elongation and reduced DRP1 levels.^231^ Upstream mTORC1 regulators can also work directly to affect proteins involved in mitochondrial dynamics. For example, AMPK phosphorylates MFF to increase DRP1 levels, thereby promoting mitochondrial fission.^119^

Among the mTORC1 downstream effectors involved in mitochondrial dynamics are 4E-BP1, S6K1 and TFEB. Phosphorylation of 4E-BP1 by mTORC1 regulates mitochondrial fission by modulating the translation of mitochondrial fission process protein 1 (MTFP1)^232^ (Fig. 4). Loss of MTFP1 is associated with increased mitochondrial fusion, whereas its overexpression leads to mitochondrial fragmentation.^232,233^ Treatment of cells with downregulated MTFP1 with rapamycin induces mitochondrial hyperfusion and branching, resulting in cell death.^232^ The deletion of S6K1 in mouse embryonic fibroblasts enhances mitochondrial fission by increasing both total DRP1 and its S616-phosphorylated form (not a target of S6K1 but a substrate of PINK1, CDK1 and other kinases), which in turn stimulates glycolysis.^234^ The overexpression of TFEB in the hearts of mice subjected to oxidative stress reduces the expression of the Drp1 and Fis1 mRNAs, diminishing mitochondrial fission.^235^

On the other hand, changes in mitochondrial dynamics can also influence mTORC1 activity. For example, the mitochondrial phosphatase phosphoglycerate mutase 5 (PGAM5) promotes mitochondrial fission by dephosphorylating DRP1.^236^ The depletion of PGAM5 accelerates senescence via mitochondrial hyperfusion, reduces mitochondrial turnover, and increases ATP levels, ROS production, and mTOR activity via the downregulation of AMPK and TSC2.^237^

mTORC2 also regulates mitochondrial dynamics (Fig. 4). Like mTORC1, prolonged fasting activates mTORC2, promoting mitochondrial fission.^226^ During extended nutrient deprivation, mTORC2 phosphorylates and activates SGK1, which subsequently phosphorylates N-myc downstream regulated gene 1 (NDRG1) to promote mitochondrial fission, which requires MFF and not DRP1.^226^ NDRG1 recruits the small GTPase CDC42 to mitochondria, where it interacts with the ER at mitochondria-associated membranes. Multiple effectors and regulators of CDC42 also participate in fission.

In *Drosophila*, the knockdown of *Rictor* blocks high-fat diet (HFD)-induced mitochondrial fragmentation and Drp1 recruitment, leading to the development of cardiomyopathy. Interestingly, knockdown of *Akt* did not affect HFD-induced mitochondrial fission. Like mTORC2 inhibition, *Drp1* knockdown prevents HFD-induced mitochondrial fragmentation in *Rictor*-knockdown flies, whereas the overexpression of *Drp1* restores mitochondrial fission.^238^

Impaired mitochondrial dynamics can, in turn, influence mTORC2 function. Depletion of *Drp1* in skeletal muscles activates mTORC1 via AKT/mTORC2. Moreover, AKT activation, resulting from *Drp1* deletion, is caused by the mitochondrial localization of mTORC2, which translocates RICTOR to deformed mitochondria-associated membranes, producing mito-bulbs structures. RICTOR is then activated by acetylation at these structures, presumably because of its proximity to mitochondrial acetyl-coenzyme A.^239^

Taken together, both mTORC1 and mTORC2 play distinct yet interconnected roles in regulating mitochondrial dynamics, which are critical for maintaining cellular homeostasis and responding to metabolic demands.

### mTORC1 and mTORC2 in modulating communication between mitochondria and other organelles in cells

To obtain the necessary building blocks for biogenesis and maintain optimal function, mitochondria must communicate with other organelles. Unlike the ER and Golgi apparatus, which use vesicular transport to interact with different organelles, mitochondria primarily connect with other cellular compartments through contact sites, which facilitate the exchange of metabolites, lipid trafficking, and calcium signaling. The mTORC1 pathway plays a significant role in regulating mitochondrial communication with other organelles via these contact sites.

**Fig. 5:**
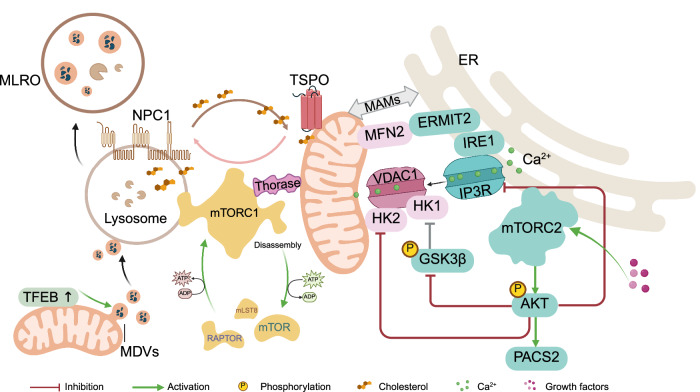
mTOR mediates mitochondria‒lysosome and mitochondria‒endoplasmic reticulum crosstalk. mTORC1 promotes mitochondria–lysosome contact site formation and facilitates cholesterol transfer by regulating the interaction between the lysosomal cholesterol exporter NPC1 and the mitochondrial translocator protein TSPO. Damaged mitochondrial proteins are transferred to lysosomes via mitochondria-lysosome-related organelles (MLROs), which are formed through the fusion of mitochondria-derived vesicles (MDVs) with lysosomes. MLROs act as an alternative mitochondrial quality control pathway, selectively degrading mitochondrial proteins via lysosomal fusion, independent of autophagy. The overexpression of TFEB enhances the clearance of MLROs. Mitochondrial–ER crosstalk is mediated by mTORC2, which promotes calcium transfer. The mitochondrial mitofusin MFN2 interacts with its splice variant ERMIT2 at the ER to tether mitochondria to the ER. The ER stress sensor inositol-requiring enzyme 1 (IRE1) is highly enriched at mitochondria-associated membranes (MAMs) and regulates calcium exchange between the ER and mitochondria. AKT-mTOR signaling suppresses IRE1 activity by promoting ER‒mitochondria contacts. Hexokinase 1 (HK1) binding to the mitochondrial VDAC promotes the entry of metabolites into the mitochondria, increasing mitochondrial respiration. In addition, mTORC2 activation of AKT promotes the phosphorylation and inhibition of MAM-related proteins, such as the 1,4,5-trisphosphate receptor (IP3R) and hexokinase 2 (HK2), to modulate calcium release. Growth factor-stimulated mTORC2 further stabilizes MERCS through AKT-mediated phosphorylation of the MERCS-associated proteins IP3R3 and phosphofurin acidic cluster sorting protein 2 (PACS2)

Mitochondria‒lysosome (M-L) contact sites facilitate the direct transfer of lipids, calcium, and other small molecules between mitochondria and lysosomes and modulate mitochondrial and lysosomal network dynamics^240^ (Fig. 5). In the yeast *S. cerevisiae*, the SEACIT/SEACAT complexes (GATOR1/GATOR2 homologs), which localize to the vacuole membrane, are involved in the maintenance of vacuole‒mitochondria contact sites (vCLAMPs).^241^ Deletion of SEACIT components decreases the number of vCLAMPs.^241^ The role of the GATOR1 complex in mammals in the formation of mitochondria–lysosomal contacts has not yet been addressed. Nevertheless, mTORC1 promotes the formation of M-L contact sites by tethering and mediating cholesterol transport from lysosomes to mitochondria. This occurs by regulating the interaction between the lysosomal protein NPC1 and the mitochondrial translocation protein TSPO.^242^ Another example involves the mitochondrial outer membrane AAA+ ATPase Thorase, which directly interacts with mTOR at the lysosomal surface without binding to the lysosome, leading to mTORC1 disassembly.^243^ The number of mitochondria‒lysosome contacts is reduced by half in the absence of Thorase. Additionally, mTOR dissociates from lysosomes upon contact with mitochondria-bound Thorase, and Thorase ATPase activity is important for this dissociation (Fig. 5).

A novel hybrid cellular structure termed mitochondria‒lysosome-related organelles (MLROs) is formed through the fusion of mitochondria‒derived vesicles (MDVs) with lysosomes, independent of DRP1. The overexpression of TFEB enhances the clearance of MLROs^244^ (Fig. 5).

Mitochondria and ER contact sites regulate calcium signaling, lipid transfer, and glucose metabolism.^245^ Protein folding in the ER is a highly energy-demanding process that requires large amounts of ATP.^246^ In turn, mitochondrial ATP synthesis depends on calcium transfer from the ER.^247^

The ER communicates with mitochondria via mitochondria-associated membranes (MAMs), which are also called mitochondria–ER contact sites (MERCSs or ERMCSs) (Fig. 5). MERCS describes the structural and architectural organization of the contact sites between mitochondria and the endoplasmic reticulum. MAMs refer to the biochemical composition, including proteins and lipids, that characterize the MERCSs (Fig. 5).

MAMs enable the bidirectional trafficking of lipids and calcium^248^ and play a role in glucose homeostasis.^245^ They also integrate signals from insulin, nutrients, and reactive oxygen species, allowing cells to adapt to autophagy and ER stress.^249^ In HeLa cells, treatment with rapamycin increases ER‒mitochondria contacts and enhances mitochondrial Ca^2+^ uptake, whereas amino acid depletion does not alter ER‒mitochondria colocalization (Fig. 5). This finding suggests that other signaling pathways, in addition to mTORC1, are probably required for the increase in organelle communication in this case.^225^

mTORC2 likely plays a more significant role than mTORC1 in the function of MAMs. mTORC2 localizes to MAMs and is essential for their integrity in a growth factor-dependent manner^250^ (Fig. 5). Growth factors activate mTORC2, which in turn phosphorylates and activates AKT, which phosphorylates and inhibits GSK3β at ER‒mitochondria contact sites,^193^ facilitating the recruitment of hexokinase 1 (HK1), which catalyzes the first step of glycolysis. The entry of metabolites into the mitochondria is facilitated via HK1 binding to the mitochondrial VDAC, which promotes mitochondrial respiration.^251^

Deletion of *Rictor* results in fewer swollen and ruptured mitochondria, impairing ER‒mitochondrial communication.^250,252^ On the other hand, MAM abrogation leads to reduced AKT-mTORC2 activity and insulin signaling.^253^

Finally, recent data show that mTORC1 is involved in mitochondrial transfer, the process by which mitochondria are transported from one cell to another, often via tunneling nanotubes (TNTs). TNTs transfer healthy mitochondria from donor cells to damaged recipient cells, thereby rescuing impaired mitochondrial function in recipient cells.^254^ ROS promote the formation of TNTs by activating PI3K/AKT/mTOR signaling, thereby facilitating the transfer of mitochondria between glial cells. This effectively increases the mitochondrial membrane potential, enhances ATP production, and reduces oxidative damage. Pharmacological inhibition of PI3K, AKT, or mTORC1 effectively blocks TNT formation.^255^

Taken together, the mTOR pathway has emerged as a key regulator of organelle contacts, which are essential not only for mitochondrial bioenergetics and biogenesis but also for broader cellular processes such as autophagy, protein folding, and insulin signaling. Furthermore, emerging evidence links mTORC1 to mitochondrial transfer between cells, underscoring its role in coordinating intercellular mitochondrial rescue mechanisms under stress. These findings highlight the central role of mTOR in orchestrating mitochondrial communication within and between cells, reinforcing its importance in maintaining mitochondrial and cellular homeostasis.

### Role of mTORC1 in the sensing of defective mitochondria

**Fig. 6:**
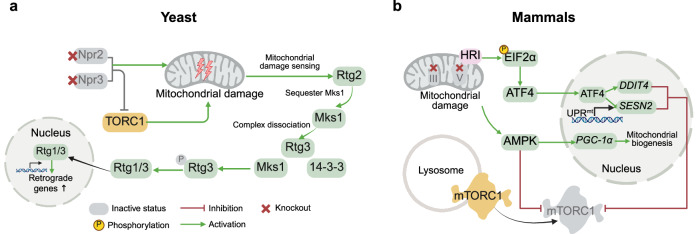
TORC1/mTORC1 sensing of mitochondrial damage. **a** In yeast, retrograde signaling is dysregulated in Npr2- and Npr3-deleted cells. Mitochondrial damage is sensed by Rtg2, which sequesters Mks1 and drives dissociation of the Mks1–Rtg3–14-3-3 complex. Dephosphorylated Rtg3 dimerizes with Rtg1, translocates into the nucleus, and promotes the transcription of RTG response genes. **b** In mammals, the inhibition of complex III or V induces mitochondrial stress that activates heme-regulated inhibitor (HRI), which signals mitochondrial damage to mTORC1 in an ATF4-dependent manner. AMPK also responds to mitochondrial damage (see Fig. 3)

mTORC1 contributes to the detection of defective mitochondria not only by regulating organelle dynamics and isolating damaged organelles through fission but also by modulating communication with the nucleus via retrograde signaling, particularly when the mitochondrial genome needs to be repaired (Fig. 6).

The mitochondrial genome, which comprises numerous mtDNA copies, is particularly vulnerable to mutations due to the absence of histones and has a mutation rate over 10 times greater than that of nuclear DNA. Excessive ROS production, often due to inefficient electron transport chain (ETC) function, can damage mtDNA^256^ and disrupt the proper folding of mitochondrial proteins, thereby initiating the unfolded protein response (UPRmt) in mitochondria. mTORC1 signaling is closely related to the response to DNA damage not only in the nucleus but also in the mitochondria.^128^

To facilitate mitochondrial DNA repair, all the proteins necessary for this process must be imported from the cytoplasm because mtDNA does not encode them. Prior to this export, the required mRNAs must be transcribed from genes encoded in the nucleus. Consequently, effective communication between the mitochondria and nucleus is essential to ensure detection of mtDNA damage signals and activation of appropriate repair mechanisms. This communication occurs via the retrograde signaling process (RTG), which was initially described in yeast.^207^ Retrograde signaling enables mitochondria under stress to relay signals to the nucleus, thereby modulating gene expression programs to adjust nitrogen and carbohydrate metabolism. In yeast, RTG was found to be deregulated in deletion mutants of NPR2 and NPR3, components of the SEACIT complex, homologs of the mammalian GATOR1 complex’s NPRL2 and NPRL3^257^ (Fig. 6). Since both SEACIT and TORC1 are localized to the vacuole membrane, information about dysfunctional mitochondria detected by vCLAMPs can be rapidly transferred to the adjacent TORC1 network to initiate degradation.^241^

To reduce the misfolded protein load in the mitochondria, the nucleus initiates the upregulation of several antioxidant enzymes, mitochondrial import proteins, chaperones, and proteases.^258,259^ One of the mTORC1 targets, the ATF4 transcription factor, was identified as an essential player in the UPRmt.^260^ Mitochondrial stress induced by treatment with antimycin A (an ETC inhibitor) or oligomycin (a mitochondrial ATP synthetase inhibitor) promotes lysosomal localization and activation of mTORC1. This leads to increased mTORC1-mediated phosphorylation of ATF4 at multiple sites and enhanced binding of ATF4 to the promoters of UPRmt genes (Fig. 6). This process is specific to mitochondrial dysfunction because disruption of mTORC1-dependent ATF phosphorylation blocks only the UPRmt but not the UPR of the ER.

Taken together, these findings indicate that mTORC1 plays a critical role as a sensor and integrator of mitochondrial dysfunction, enabling the cell to detect and respond to various forms of mitochondrial stress. It monitors mitochondrial health through multiple inputs, including changes in energy status, redox imbalance, impaired oxidative phosphorylation, and mtDNA damage. These dysfunctions activate retrograde signaling pathways that converge on mTORC1 to modulate its activity on the basis of the severity and duration of mitochondrial stress. However, the most critical outcome of the mTORC1 response to mitochondrial damage is the initiation of mitophagy.

### mTORC1 in the regulation of mitophagy

**Fig. 7:**
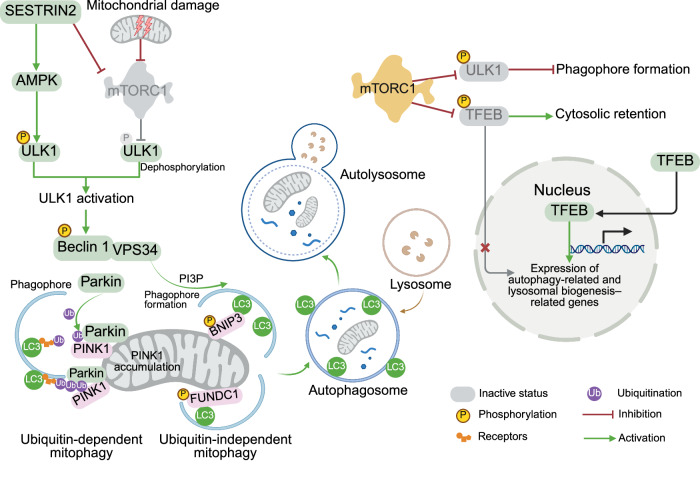
mTORC1 regulation of ubiquitin-dependent and ubiquitin-independent mitophagy. mTORC1 regulates autophagy and mitophagy to eliminate damaged mitochondria. mTORC1 phosphorylates ULK1 and TFEB to inhibit phagophore formation and lysosomal biogenesis. Upon mitochondrial damage or the inhibitory effect of SESTRIN2, mTORC1 is inactivated and dissociates from lysosomes, allowing ULK1 dephosphorylation and AMPK-mediated phosphorylation. Active ULK1 then phosphorylates BECLIN 1 and recruits VPS34 to nucleate the phagophore, triggering PINK1/PARKIN (ubiquitin-dependent) mitophagy and BNIP3- or FUNDC1-mediated (ubiquitin-independent) mitophagy

The elimination of defective mitochondria or organelles in excess is one of the essential functions of mTORC1, which controls both general autophagy and mitophagy^21^ (Fig. 7), whereas mTORC2 is not directly involved in these processes. Overall, the mechanisms by which mTORC1 and AMPK induce autophagy are similar to the mechanisms involved in initiating mitophagy. Both mTORC1 and AMPK are implicated in removing mitochondria at the basal level and through mitophagy when damage to the organelle is excessive.

Mitophagy can be classified into two types on the basis of how mitochondria prime for degradation: ubiquitin-dependent (PINK-PARKIN mitophagy) and ubiquitin-independent pathways. In PINK-PARKIN mitophagy, the protein kinase PTEN-induced kinase 1 (PINK1) accumulates on the outer membrane of defective mitochondria, leading to ubiquitin phosphorylation and the recruitment of the E3 ubiquitin ligase PARKIN. In contrast, PINK1-PARKIN independent mitophagy relies on mitophagy receptors, mitochondrial membrane proteins (e.g., BNIP3, BNIP3L/NIX, FKBP8, FUNDC1), which can interact with LC3 or GABARAP proteins to recruit dysfunctional mitochondria to autophagosomes.^261^

PINK-PARKIN mitophagy can be induced upon rapamycin treatment in cells harboring either large-scale partial deletions of mtDNA or complete depletion of mtDNA.^262^ Importantly, the mitophagic response in such cells requires both mTORC1 inhibition and loss of mitochondrial membrane potential, which activate PARKIN recruitment to mitochondria. Interestingly, mtDNA mutations that impair other mitochondrial functions, such as reduced ATP synthesis or respiratory chain function, are not important for PARKIN recruitment. As a result, mitophagy in these cells is not effective.

Recently, a new mechanism for regulating PINK-PARKIN mitophagy involving mTORC1 was described. Clustered mitochondrial homolog (CLUH) is a cytosolic protein that interacts with cytoplasmic mRNAs encoding mitochondrial proteins, facilitating their translation and distribution.^263^ Dysfunctional mitochondria that accumulate due to impaired protein distribution are likely candidates for mitophagy. CLUH RNP particles act as spatial compartments, preserving mRNAs involved in mitochondrial catabolic pathways and promoting their translation. Additionally, they function as signaling hubs by recruiting mTORC1. Upon starvation, CLUH inhibits mTORC1 activation and mitochondrial anabolic pathways, promoting mitochondrial turnover and allowing efficient metabolic reprogramming. In the absence of CLUH, mitophagy impairment causes mitochondrial clustering to be rescued by rapamycin treatment.^264^ Interestingly, a constitutive lack of CLUH accelerates autophagy but inhibits mitophagy.

Another reason for mTORC1-related mitophagy defects is linked to mTORC1 hyperactivation due to the loss of function of its upstream negative regulators, such as mammalian TSC2,^265,266^ SESTRIN2^267^ and the SEACIT complex in yeast.^241,268^ For example, *Tsc2*-deficient neurons progressively accumulate mitochondria in their soma while becoming depleted of functional mitochondria in axons.^265^ Suppressing mTORC1 hyperactivity with rapamycin or inducing mTOR-independent autophagy with carbamazepine can restore the clearance of damaged mitochondria in *Tsc2*-deficient neurons. A similar effect was observed in pancreatic *β* cells, where *Tsc2* knockout in cells treated with the mitochondrial uncoupler CCCP resulted in a reduced Δψm. mTORC1 hyperactivation and the accumulation of damaged mitochondria with collapsed mitochondrial membrane potential are accompanied by a reduction in PINK1 expression and impaired translocation of PARKIN to uncoupled mitochondria.^266^ The mitophagy defects in *Tsc2*-deleted cells were rescued by inhibition of mTORC1 with rapamycin.

In addition to its function as a leucine sensor, SESTRIN2 is a stress-inducible antioxidant protein and one of the upstream AMPK activators^269^ (Fig. 7). It can negatively regulate mTORC1 through two parallel mechanisms: via AMPK during energy sensing and via GATOR2 during amino acid sensing. In mammalian cells, SESTRIN2 interacts with ULK1 and facilitates ULK1-mediated phosphorylation of BECLIN1, which is required for BECLIN1 binding with PARKIN prior to mitochondrial translocation. SESTRIN2 downregulation inhibits the interaction between BECLIN1 and PARKIN, preventing optimal mitochondrial accumulation of PARKIN.^267^

Finally, during PARKIN-dependent mitophagy, p62, which is phosphorylated by mTORC1, is recruited to ubiquitinated cargo and accumulates on damaged mitochondria.^270^ This phosphorylation enhances the binding of p62 to ubiquitinated substrates and LC3, thereby promoting autophagosome engulfment and subsequent lysosomal degradation of both p62 and damaged mitochondria.

In BNIP3-mediated mitophagy in mammals, ULK1 phosphorylates BNIP3, enhancing its interaction with LC3 and reducing BNIP3 proteasomal degradation of^271,272^ (Fig. 7). Phosphorylated BNIP3 more effectively facilitates the formation of autophagosomes.^273^ The mitochondrial localization of BNIP3 and the translocation of endonuclease G (ENDOG) from mitochondria to the nucleus are crucial steps in BNIP3-related autophagy.^274^ ENDOG suppresses the mTORC1 pathway by interacting with 14-3-3γ, which releases TSC2.^275^ Cytoplasmic ENDOG can also bind to 14-3-3γ to release RICTOR, activating mTORC2 and promoting acetyl-CoA production.^276^

Interestingly, accumulation of the BNIP3 paralog BNIP3L/NIX protein not only triggers mitophagy but also activates the mTORC1 pathway. This phenomenon can be observed during lipotoxicity in muscles, where lipid accumulation leads to mitochondrial dysfunction and insulin resistance. BNIP3L/NIX expression is elevated in the muscle tissue of rodents fed a high-fat diet.^277^ This results in mitochondrial depolarization and mitophagy.^278,279^ In parallel, BNIP3L/NIX accumulation modulates RHEB-dependent phosphorylation of S6K1 by mTORC1. mTOR signaling is involved in a negative feedback pathway that limits glucose uptake through S6K1-mediated phosphorylation and suppression of IRS1.^278^ Impaired glucose uptake, in turn, stimulates BNIP3L-dependent mitophagy.

Another form of mitophagy that is considered ubiquitin independent is FUNDC1-mediated mitophagy. Under hypoxic conditions, ULK1 phosphorylates the mitochondrial outer membrane protein FUNDC1, enhancing its binding to LC3 and thereby initiating receptor-mediated mitophagy to remove damaged mitochondria.^280^ In doxorubicin-injured cardiomyocytes, AMPK phosphorylation activates ULK1, which subsequently elevates FUNDC1 and LC3-II/I levels, resulting in increased mitophagic flux.^281^ Conversely, pharmacological inhibition of either AMPK or ULK1 abolishes FUNDC1-mediated mitophagy. Notably, ULK1 can also be locally activated independently of both AMPK and mTOR signaling.^282^

Yeast lacks direct homologs of mammalian PARKIN, PINK1, BNIP3, and BNIP3L but compensates with a unique mitophagy pathway centered on proteins such as Atg32.^283^ These differences reflect evolutionary divergence between unicellular and multicellular organisms in handling mitochondrial quality control. Nevertheless, the mTORC1 pathway is also involved in mitophagy regulation in yeast. Thus, in *S. cerevisiae*, cells lacking the SEACIT complex exhibited significant suppression of mitophagy during prolonged respiratory growth, whereas other types of selective autophagy were less affected.^241,268,284^ Treatment with rapamycin or deletion of the mTORC1-stimulating RAGA homolog Gtr1 downstream of SEACIT rescued mitophagy defects.^268^ Whether GATOR1 is involved in mitophagy in mammals remains to be determined.

In conclusion, mTORC1 plays a crucial role in mitochondrial degradation, ensuring proper cellular homeostasis. Alterations in mitochondrial mass due to impaired biogenesis, dynamics, and degradation ultimately impact mitochondrial functionality. As mTOR complexes play crucial roles in regulating these processes, they are indispensable for maintaining optimal mitochondrial performance.

### mTORC1 in the regulation of mitochondrial respiration: the TCA cycle, ETC and OXPHOS

**Fig. 8:**
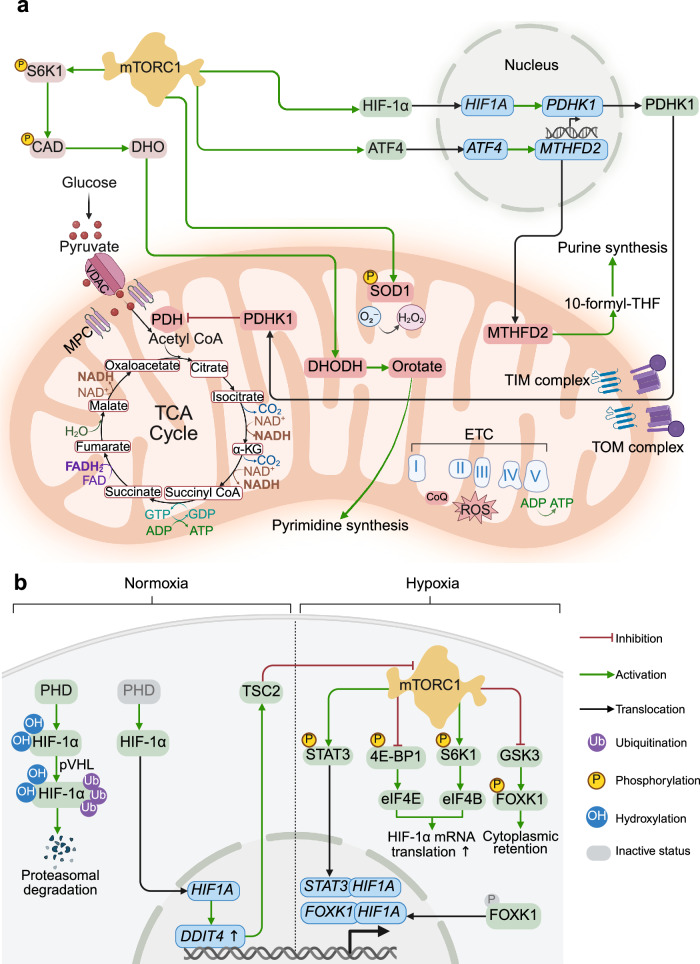
mTOR regulates the TCA cycle, OXPHOS nucleotide biosynthesis, and hypoxia. **a** Before initiating the TCA cycle in mitochondria, glucose is first metabolized through glycolysis in the cytosol, generating pyruvate. Pyruvate then diffuses through VDAC in the outer mitochondrial membrane and is imported into the matrix by the mitochondrial pyruvate carrier (MPC). In the matrix, pyruvate dehydrogenase (PDH) converts pyruvate into acetyl-CoA, linking glycolysis to the TCA cycle and fueling OXPHOS. Inhibition of PI3K/mTOR signaling enhances phosphorylation of the PDH E1α subunit, thereby suppressing PDH activity. mTORC1 promotes pyrimidine and purine synthesis through phosphorylating S6K1. mTORC1 promotes purine synthesis through ATF4. ATF4 induces the expression of the mitochondrial enzyme MTHFD2, which generates the 10-formyl-THF required for purine biosynthesis. mTORC1 promotes de novo pyrimidine synthesis via S6K1-mediated phosphorylation and activation of CAD, which catalyzes the early steps of the pathway, leading to dihydroorotate (DHO), which is subsequently converted by dihydroorotate dehydrogenase (DHOH) toward pyrimidine synthesis. mTORC1 promotes the phosphorylation of the superoxide dismutase SOD1 to decrease DHOH activity. **b** Under normoxia (left panel), prolyl hydroxylase (PHD) hydroxylates HIF-1α for pVHL-mediated proteasomal degradation. In the absence of PHD, HIF-1α is stabilized and translocates to the nucleus. HIF-1α induces DNA damage-inducible transcript 4 (DDIT4), which activates TSC2 to inhibit mTORC1. Under hypoxia (right panel), mTORC1 promotes the phosphorylation of 4E-BP1 and S6K1 to increase HIF-1α mRNA translation, whereas GSK3 inhibition-mediated FOXK1 translocation and STAT3 translocation further facilitates HIF-1α and glycolytic gene expression. TCA cycle, tricarboxylic acid cycle; ETC, electron transport chain; O₂⁻, superoxide; H₂O₂, hydrogen peroxide; CoQ, coenzyme Q (ubiquinone); pVHL, von Hippel–Lindau protein

The regulation of mitochondrial respiration is one of the key functions of mTORC1.^285^ Mitochondrial respiration involves glycolysis in the cytoplasm, followed by the TCA (Krebs) cycle in the mitochondrial matrix (Fig. 8a). The electrons generated from these metabolic processes are transferred to the electron transport chain located in the inner mitochondrial membrane, where oxidative phosphorylation takes place, producing the majority of the cellular ATP.

Glucose is crucial for supplying the necessary substrates for mitochondrial respiration. When glucose uptake is impaired, mitochondrial respiration is negatively affected, leading to decreased ATP levels (Fig. 3). The mTORC1 pathway is involved in the expression of glucose transporters through the upregulation of PGC-1α and in the maintenance of GLUT1 transporters at the plasma membrane.^286^ Under high-glucose conditions, active mTORC1 promotes the expression of YY1, which then translocates to the nucleus and inhibits PGC-1α transcription. This inhibition results in a reduced mitochondrial membrane potential and decreased ATP production.^287^

During exercise, glucose uptake is independent of mTORC1 and PGC-1α but instead is mediated by TFEB. The dephosphorylation of TFEB, which is necessary for its activation and translocation to the nucleus, is mediated by the calcium-dependent phosphatase calcineurin.^288^ Exercise-induced calcium influx activates calcineurin. During physical activity, TFEB translocates to the nucleus of skeletal muscle cells and regulates glucose uptake and glycogen content by controlling the expression of glucose transporters (e.g., GLUT4), glycolytic enzymes (e.g., hexokinase), and pathways related to glucose homeostasis (e.g., nitric oxide synthetase).^289^ In addition, AMPK and mTORC1 regulate mitochondrial respiration by controlling the interaction between the RNA-binding protein SMAUG1 and nuclear transcripts encoding mitochondrial enzymes.^290^

When glucose is abundant, it can be converted to glycogen for storage. Glycogen synthesis is modulated in an mTORC1-dependent manner.^291^ In the absence of TSC2, hyperactivated mTORC1 phosphorylates S6K1 and inactivates GSK3β, leading to the dephosphorylation and activation of glycogen synthase, which promotes glycogen synthesis.^291^ Furthermore, because mTORC1 inhibits ULK1, autophagy is impaired, affecting glycogen clearance.^291^

When a cell requires a sustained energy supply, glucose is converted into pyruvate through glycolysis. mTORC1 regulates pyruvate oxidation through the TCA cycle to promote OXPHOS^200^ (Fig. 8a). Pyruvate dehydrogenase (PDH) is the enzyme that catalyzes the transformation of pyruvate to acetyl-CoA, linking glycolysis and the TCA cycle to drive ATP production through OXPHOS. Inhibition of PI3K/mTOR signaling leads to increased phosphorylation of the E1α subunit of the PDH, inhibiting its activity. This results in decreased mitochondrial respiration capacity due to impaired pyruvate entry into the TCA cycle.^292^

Conditional knockout of the GATOR1 subunit NPRL2 in skeletal muscle leads to chronic mTORC1 activation, which reduces glucose entry into the TCA cycle and decreases the levels of citrate and fumarate.^293^ In *S. cerevisiae* strains lacking the NPRL2 homolog Npr2, the levels of TCA cycle metabolites such as citrate, succinate and α-KG are also significantly reduced.^294^

The reversible hydration of fumarate to malate is catalyzed by fumarase. Fumarase deficiency halts the TCA cycle, leading to fumarate accumulation, which in turn contributes to the upregulation of hypoxia-inducible factor-1α (HIF-1α), a transcription factor that facilitates cellular adaptation to hypoxia by promoting aerobic glycolysis.^295,296^ mTORC1 regulates both glycolysis and mitochondrial oxygen consumption by controlling the transcription and translation of HIF-1α^297,298^ (Fig. 8b). Specifically, mTORC1 stimulates the expression of glycolytic enzymes by upregulating HIF-1α through the transcription factor Forkhead box K1, thereby promoting glycolysis rather than OXPHOS.^299^ Conversely, OXPHOS contributes to lipid metabolism by maintaining phosphatidic acid levels and supporting mTORC1 activity through mtDNA replication. This helps suppress excessive HIF-1α accumulation and maintains metabolic balance.^300^

During hypoxia, mTORC1 promotes HIF-1α expression by directly phosphorylating signal transducer and activating transcription 3 (STAT3) to stimulate HIF-1α mRNA transcription^301^ (Fig. 8b). mTORC1 stabilizes and controls the translation of HIF-1α through the phosphorylation of 4E-BP1 and S6K1.^301^ Under normoxia, HIF-1α is hydroxylated by prolyl-hydroxylases (PHDs) and then recognized and degraded by the ubiquitin‒proteasome system, maintaining its low intracellular levels. Inhibition of PHD leads to suppressed phosphorylation of mTORC1, S6K1 and 4E-BP1, promoting HIF-1α expression.^302^

mTORC1 inhibition through *Raptor* suppression decreases mitochondrial basal respiration and diminishes ATP production.^303^ In addition, mTORC1 inhibition reduces the expression levels of proteins from ETC complexes I–V, whereas mTORC2 inhibition does not affect the expression of ETC proteins. Nevertheless, mTORC2 is also involved in regulating mitochondrial function. The absence of mTORC2 leads to a decrease in the levels of TCA cycle intermediates, especially α-KG.^151^ In *S. cerevisiae*, TORC2 and Ypk1 (S6K1 homolog) control calcineurin activity, modulating mitochondrial respiratory activity.^304^

Importantly, mitochondrial dysfunction also alters mTORC1 activity, primarily through reduced oxidative phosphorylation, which lowers ATP production and increases the AMP/ATP ratio, activating AMPK, which phosphorylates TSC2 and RAPTOR, leading to mTORC1 inhibition and a shift toward energy conservation and stress adaptation.^115,116^ Simultaneously, AMPK activation promotes mitochondrial recovery by stimulating PGC-1α.^209^ Thus, AMPK coordinates a dual response to mitochondrial stress: temporarily suppressing energy-intensive growth to conserve resources and enhancing the regenerative capacity of organelles to recover their function and energy supply.

A recent genome-wide screening design to identify mTORC1 regulators further highlighted the importance of mitochondrial health in mTORC1 function.^305^ This screen revealed that not only AMPK but also another kinase, HRI, signals mitochondrial distress to mTORC1 (Fig. 6). Through its action on ATF4, HRI upregulates SESTRIN2 and REDD1 (stress-induced protein, generally upregulated by DNA damage and ROS). AMPK inhibits mTORC1 shortly after mitochondrial dysfunction begins, whereas HRI do so at later stages. Loss of AMPK and HRI renders mTORC1 signaling resistant to mitochondrial dysfunction induced by oligomycin and antimycin treatments. Another example is the suppression of mTORC1 due to chronic energy stress and elevated AMPK activity in cells with mitochondrial DNA damage. Finally, high levels of ROS produced by defective mitochondria inactivate mTORC1 and initiate mitophagy.

### Role of mTOR complexes in mitochondrial ROS production

Mitochondria generate approximately 90% of cellular ROS,^306^ which are primarily produced during OXPHOS in the ETC. The main sites of mitochondrial ROS production are complex I (NADH dehydrogenase) and complex III (cytochrome bcI complex). At these sites, electrons can leak and interact with molecular oxygen, leading to the formation of superoxide, the primary ROS produced in mitochondria. Superoxide is then converted to hydrogen peroxide by the antioxidant enzymes superoxide dismutases (SOD1 and SOD2). mTORC1 promotes the phosphorylation of SOD1 to decrease its activity.^307^

High levels of ROS lead to the inactivation of mTORC1, which functions as a feedback mechanism. This inactivation reduces the number of mitochondria through mitophagy, thereby preventing further ROS production.^308^ Interestingly, low ROS levels can activate mTORC1 through the oxidation of cysteine residues in the mTORC1 complex itself^309^ or in RHEB.^310^ Rather than causing damage solely, mitochondrial ROS can initiate stress responses and trigger transcriptional changes in the nucleus through retrograde signaling. The RTG pathway can activate stress-defense mechanisms and even help eliminate signaling molecules, including ROS. In addition, ROS can promote autophagy, which in turn reduces oxidative stress.^311,312^ These “beneficial” ROS can protect cells from additional stress-related damage.^313^

Several upstream regulators of mTORC1 are crucial in shaping cellular responses to ROS and influencing mTORC1/mitochondria interactions. For example, overexpression of NPRL2, an mTORC1 inhibitor from the GATOR1 complex, promotes ROS production by activating the NOX2 enzyme and exacerbating mitochondrial dysfunction.^314^

Another key player in the mitochondria-ROS-mTORC1 axis is SESTRIN2, which regulates mitochondrial respiration^315^ and exhibits antioxidant properties that mitigate ROS levels.^311,316^ Structural studies on SESTRIN2 suggest that it may act as a direct ROS scavenger.^317^ Moreover, SESTRIN2 expression is upregulated by UVA exposure (315–400 nm) via mitochondrial ROS, and its absence inhibits UVA-induced ROS production.^318^ Therefore, mitochondrial damage caused by UVA contributes to SESTRIN2 induction.

Recently, mTORC2 has emerged as a crucial mediator of the cellular response to intracellular and mitochondrial ROS, particularly under pathological conditions. For example, in mutant KRAS-driven colorectal cancer cells, drug-induced activation of mutant KRAS promoted mitophagy and mitochondrial fission via phosphorylation of AKT and ROS generation, leading to cell death.^319^ During stress conditions in the brain, high levels of ROS contribute to astrocytes dysfunction, which consequently impairs tissue regeneration or aggravates neurotoxicity. Mitochondrial NADPH oxidase (NOX4) is a major source of ROS production. Increased NOX4-dependent ROS production causes the oxidation of RICTOR, impaired mTORC2 activation and AKT phosphorylation during astrocyte differentiation.^320^

In summary, mitochondrial ROS are not only byproducts of cellular metabolism but also serve as dynamic signaling molecules that influence a range of cellular processes. Their dual role is exemplified by their ability to inactivate mTORC1 at high concentrations, triggering protective processes such as mitophagy, whereas at lower concentrations, they can activate mTORC1 and promote cell survival pathways. Thus, ROS orchestrate a feedback system that not only maintains mitochondrial health and energy balance but also determines cell fate.

### mTORC1 functions in nucleotide biosynthesis

The interplay between mTORC1 and mitochondria plays a central role in coordinating nucleotide biosynthesis with cellular energy and nutrient availability (Fig. 8a). Together, they regulate the production of purines and pyrimidines by integrating metabolic signals to ensure efficient growth and proliferation.^321^ Mitochondria provide the energy, precursors and cofactors required for nucleotide biosynthetic pathways. ATP generated by mitochondrial oxidative phosphorylation is required for energy-intensive steps in purine and pyrimidine synthesis. The TCA cycle provides precursors for nucleotide synthesis, such as aspartate and fumarate. Aspartate is synthesized from oxaloacetate in the mitochondria, whereas fumarate, released during purine biosynthesis, is fed back into the TCA cycle. In parallel, mTORC1 senses the AMP/ATP ratio via AMPK and promotes mitochondrial oxidative phosphorylation to ensure sufficient ATP for nucleotide synthesis. mTORC1-driven mitochondrial activity maintains high levels of oxaloacetate, which is transaminated to produce aspartate.

Carbamoyl-phosphate synthetase 2, aspartate transcarbamylase, and dihydroorotase (CAD) catalyze the first three steps of de novo pyrimidine synthesis. mTORC1 promotes pyrimidine synthesis through S6K1-dependent phosphorylation of CAD.^178^ In addition, RHEB^322^ and mLST8^323^ interact with CAD and upregulate its activity. Loss of the mitochondrial NAD^+^-dependent deacetylase sirtuin 3 results in mTORC1 activation, induction of mTORC1-dependent CAD phosphorylation and increased pyrimidine synthesis.^324^

mTORC1 also promotes purine synthesis via the transcription factors ATF4, MYC and FOXK1,^177,299^ which control a variety of metabolic enzymes that contribute to purine biosynthesis. The expression of mitochondrial methylenetetrahydrofolate dehydrogenase 2 (MTHFD2), which is necessary for purine synthesis, depends on ATF4.^177^ In B cell germinal centers, however, MTHFD2 expression is driven by YY1 rather than by ATF4.^325^ Depletion or inhibition of MTHFD2 leads to a reduction in mTORC1 activity in B cells, whereas supplementation with purines and the antioxidant glutathione effectively restores mTORC1 activity in MTHFD2-deficient cells.

mTORC1 also stimulates the formation of “purinosomes”, enzyme complexes on the mitochondrial surface dedicated to de novo purine synthesis.^326^ mTOR inhibition reduces purinosome–mitochondrion colocalization and suppresses the purinosome formation stimulated by mitochondrial dysregulation.

In summary, mTORC1 and mitochondria synchronize cellular growth with metabolic capacity, ensuring that nucleotides are produced when energy and nutrients are sufficient. mTORC1 not only enhances mitochondrial ATP production and supplies biosynthetic precursors but also directly regulates key enzymes and transcription factors involved in purine and pyrimidine synthesis. This integration ensures that cells maintain a balanced supply of nucleotides to support proliferation and stress adaptation. Disruption of this axis impairs nucleotide metabolism and compromises mTORC1 activity itself, highlighting a reciprocal relationship that is critical for cellular growth and homeostasis.

### mTORC1 and mitochondria in the regulation of cell death

mTORC1 and mitochondria are involved in modulating different forms of programmed cell death, including apoptosis, ferroptosis, cuproptosis, and pyroptosis. mTORC1 does not directly “select” a specific cell death pathway but rather modulates cellular conditions, such as energy status, redox balance, and autophagy activity, that favor or suppress distinct forms of cell death. For example, mTORC1 promotes survival by inhibiting apoptosis and autophagy, while its inhibition can sensitize cells to autophagy-dependent death, ferroptosis, or, in certain contexts, necroptosis and pyroptosis. This regulatory flexibility allows mTORC1 to influence cell fate decisions in a context-dependent manner.

The communication between mTORC1 and mitochondria during apoptosis is influenced by the metabolic state, ROS production, mitochondrial dynamics, and autophagy.^327^ BCL-associated X (BAX) and BCL2 homologous antagonist/killer (BAK) are key effectors that initiate the apoptotic pathway. They form pores on the OMM, increasing membrane permeability and allowing the release of mitochondrial apoptogenic factors such as cytochrome *c* into the cytosol.^328^ Consequently, cytochrome *c* binds to apoptotic protease-activating factor 1 (APAF-1), recruiting and activating caspase-9. This initiates the formation of apoptosomes and activates a cascade of downstream caspases, leading to cell death.^329,330^

mTORC1 can also inhibit apoptosis through autophagy; conversely, inactivation of mTORC1 can induce apoptosis by promoting autophagy.^331^ Necroptosis, pyroptosis and ferroptosis are also regulated by mTORC1 through autophagy.^327^

Ferroptosis is a type of programmed cell death dependent on the accumulation of lipid peroxides. One of the reasons for this accumulation is an increased mitochondrial membrane potential that occurs upon cysteine deprivation.^332^ Cysteine is an essential amino acid that is important for the synthesis of the antioxidant glutathione, which protects cells from oxidative stress. Cysteine activates mTORC1 and facilitates the synthesis of the GPX4 enzyme, which suppresses ferroptosis by using the antioxidant glutathione to remove lipid peroxides.^333^ The inhibition of mTORC1 by Torin1 prevents ferroptosis, restoring mitochondrial ETC dysfunction and promoting the reformation of the tubular mitochondrial network.^334^ Thus, mTORC1 regulates ferroptosis both by autophagy and by modulating GPX4 activity.

Cuproptosis is triggered by the accumulation of copper ions, which target components of the TCA cycle. This binding leads to aberrant protein aggregation and destabilization of iron‒sulfur cluster proteins, causing mitochondrial stress, oxidative damage, and eventual cell death. Ferredoxin 1 (FDX1), a reductase that reduces Cu^2+^ to its more toxic Cu^1+^ form, and a number of factors involved in protein lipoylation (e.g., dihydrolipoamide S-acetyltransferase, DLAT) are key regulators of cuproptosis.^335^ The upregulation of DLAT is associated with mitochondrial dysfunction, activation of the PI3K/mTOR pathway, autophagy suppression, and eventual cell death.^336^

Pyroptosis is a pro-inflammatory form of programmed cell death characterized by gasdermin-mediated membrane pore formation and the release of IL-1β and IL-18.^337^ Pyroptosis is typically initiated in response to lipopolysaccharide, extracellular ATP, mitochondrial DNA leakage, and certain chemical agents, such as cisplatin. These stimuli activate cytosolic pattern recognition receptors (PRRs), leading to the assembly of inflammasomes and culminating in membrane rupture and inflammatory cytokine release. mTORC1 inhibition via rapamycin can attenuate pyroptosis by reducing mitochondrial ROS and activating mitophagy, thereby limiting inflammasome activation. Adrenomedullin, a cytoprotective peptide hormone, further enhances this protective effect by activating the ROS–AMPK–mTOR axis, increasing mitochondrial clearance and suppressing pyroptotic signaling.^338^ mTOR also influences pyroptosis through the STAT3, PPARγ, NRF2, and metabolic signaling pathways, underscoring its multifaceted role in controlling inflammatory cell death.^339,340^

## mTOR in physiology

### mTOR signaling in metabolic tissues

**Fig. 9:**
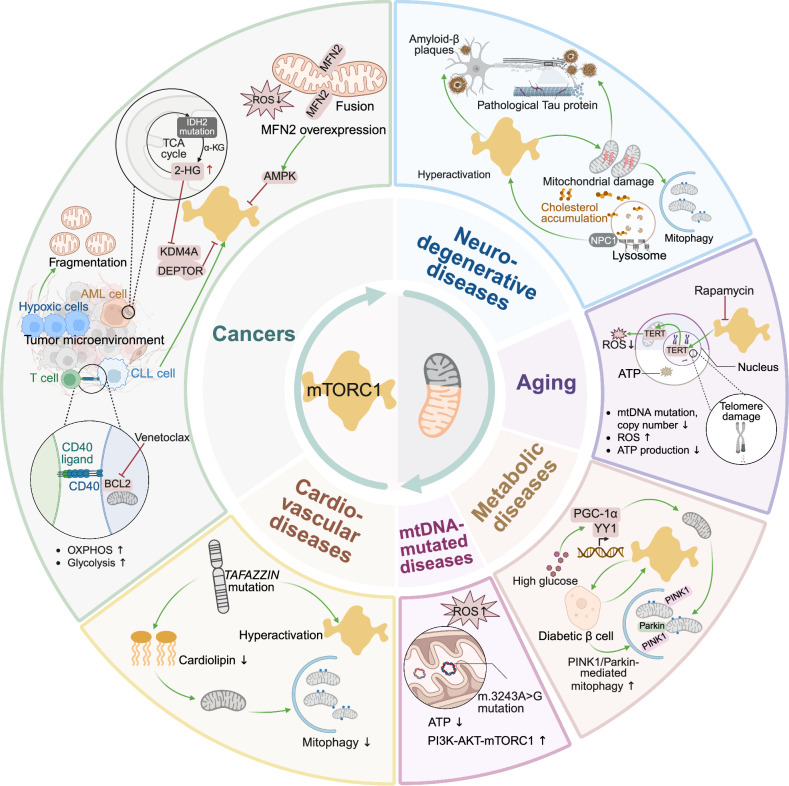
Deregulation of mTORC1 signaling and mitochondrial dysfunction in human diseases. Schematic illustration showing crosstalk between mTORC1 signaling and mitochondrial homeostasis under different pathological conditions. Representative disease contexts are indicated: cancer (AML, CLL), neurodegenerative disorders (Alzheimer’s disease with amyloid-β plaques and tau pathology), aging, metabolic disorders (diabetes, obesity), mtDNA mutation syndromes and cardiovascular diseases. Key mechanisms include altered fusion–fission balance (MFN2), impaired mitophagy (PINK1/Parkin), dysregulated bioenergetics (ATP, ROS), and transcriptional regulation (YY1/PGC-1α). Perturbations in the tumor microenvironment, hypoxia (KDM4A, IDH2 mutation, 2-HG), and therapeutic interventions (rapamycin, venetoclax, DEPTOR) are also illustrated

While mTOR is active across all tissues, its regulatory effects are especially critical in metabolic organs such as the liver, skeletal muscle, and adipose tissue, which maintain glucose and lipid homeostasis. These tissues respond dynamically to nutrient levels, hormonal cues, especially insulin, and the cellular energy status, converging on mTOR complexes to determine whether cells undergo anabolic growth or catabolic breakdown (Fig. 9).

In the liver, mTOR signaling modulates energy metabolism in response to feeding and fasting. During fasting, hepatic glucose production occurs through glycogenolysis, which prevents hypoglycemia. Following a meal, glycolysis, glycogenesis, and lipogenesis become dominant.^341^ During feeding, elevated insulin coactivates mTORC1 and mTORC2, facilitating nutrient storage and tissue growth. In contrast, fasting lowers insulin, shifting the metabolic program toward autophagy.^342^ This transition is regulated by mTORC1 inhibition, enabling the degradation of cellular components to fuel gluconeogenesis. Fasting-induced liver mass loss (~25%) is abolished in mice lacking TSC1, RAPTOR,^343^ or ATG7,^344^ confirming the role of mTORC1 in switching from anabolism to catabolism. Mice with liver-specific knockouts of *Raptor* or *Rictor* exhibit hyperglycemia, glucose intolerance, and insulin resistance, mirroring aspects of type 2 diabetes.^345,346^

The role of mTORC1 in liver fat synthesis is context dependent. Liver-specific mTORC1-deficient mice show reduced triglycerides and cholesterol only under high-fat diets, suggesting that mTORC1 is essential for hepatic steatosis under pathological conditions.^182^ This is relevant for understanding selective hepatic insulin resistance, where insulin fails to suppress gluconeogenesis but continues to activate lipogenesis, paradoxically causing both hyperglycemia and hyperlipidemia.^347^ In contrast to this model, mice with deletions of lymphatic *Tsc1*, which demonstrate persistent mTORC1 activation, are protected from hepatic steatosis.^348^ This is due to impaired AKT signaling via mTORC1-mediated negative feedback loop, as both AKT and mTORC1 are independently essential for full lipogenesis.^343,349^ AKT promotes lipogenesis by suppressing INSIG2a, an SREBP inhibitor, whereas mTORC1 facilitates SREBP activation.

The role of mTORC2 plays an equally significant role in the liver is, acting as a key integrator of hepatic glucose and lipid metabolism. Liver-specific *Rictor* knockout mice exhibit total hepatic insulin resistance, as evidenced by hyperglycemia and defective lipogenesis.^345,346,350^ This results from impaired AKT phosphorylation, which reduces SREBP-1c activity. Rescue of AKT signaling restores lipogenesis, demonstrating that mTORC2 controls SREBP-1c via AKT but not through INSIG2a suppression.^346^ Additionally, mTORC2 enhances glycolysis through the activation of glucokinase and the transcription factor ChREBP and suppresses gluconeogenesis by limiting nuclear FOXO1, a major gluconeogenic regulator.

Mice are unable to suppress mTORC1 during fasting because of constitutively active RAGA and suffer lethal hypoglycemia due to failure of autophagy and energy conservation.^351^ Similar fatal outcomes occur in mice lacking SESTRINs or autophagy-related genes.^352,353^

mTOR complexes are vital for maintaining muscle mass. In skeletal muscle, mTORC2–AKT signaling enhances glucose uptake and glycogen synthesis, whereas mTORC1 supports protein synthesis from circulating amino acids. Muscle-specific deletions of mTOR^354^ or Raptor^355^ lead to progressive muscle dystrophy, diminished oxidative capacity, and glycogen accumulation, despite hyperactivation of AKT caused by disruption of mTORC1 feedback inhibition, which is mediated through S6K1–IRS1 signaling. Cardiac-specific deletions of mTOR^356^ or Raptor^357^ result in dilated cardiomyopathy, which is attributed to loss of 4E-BP1 repression, leading to diminished protein synthesis and heart failure. Disruption of GATOR1 function in skeletal muscle leads to sustained mTORC1 activation, both in fed and fasted states.^293^ Muscle-specific NPRL2 knockout mice exhibit hypertrophic fibers and a shift in fiber-type distribution, characterized by an increase in fast-twitch glycolytic fibers and a reduction in slow-twitch oxidative fibers. These animals also display modified running performance and improved glucose tolerance.

White adipose tissue is the body’s principal energy reservoir, and mTOR signaling regulates adipogenesis, lipid storage, and energy consumption. mTORC1 is essential for the differentiation of preadipocytes via the activation of PPARγ and SREBP transcription factors, which play essential roles in adipogenesis.^358–360^ Mice lacking S6K1 have a lean phenotype and are protected from obesity,^361^ whereas the deletion of 4E-BPs leads to increased obesity and decreased energy expenditure.^362^ mTORC1 inhibition via rapamycin blocks adipocyte differentiation, whereas hyperactivation through TSC2 deletion enhances fat cell formation.^363^ Adipose-specific Raptor knockout mice are lean, resistant to diet-induced obesity, and show enhanced energy expenditure.^358^ These results underscore the mTORC1–S6K1 axis as the major effector controlling adipose physiology.

mTORC2 in adipose tissue affects systemic growth and metabolism through endocrine mechanisms. Adipose-specific *Rictor* knockout mice exhibit increased lean mass, likely due to elevated hepatic IGF-1 and pancreatic insulin secretion, as mTORC2 in white adipose tissue negatively regulates these hormones via feedback loops.^364^

Together, these findings highlight the tissue-specific complexity of mTOR signaling in metabolic regulation. The precise tuning of mTOR activity across these organs is critical for whole-body metabolic homeostasis, and its dysregulation contributes to the pathogenesis of insulin resistance, obesity, and type 2 diabetes.

### mTOR in immunity

mTORC1 is essential for regulating immune cell metabolism, differentiation and effector functions.^365^ In the resting state, T lymphocytes rely on catabolic pathways and autophagy to generate essential biomolecules for maintaining protein synthesis and energy production. Upon activation, these cells undergo a metabolic shift toward anabolism, increasing glycolysis to meet the demands of rapid proliferation. This transition is tightly regulated by mTORC1, which activates key metabolic programs required for T-cell activation. CD4^+^ T cells differentiate into various effector subtypes, including Th1, Th2, and Th17, in response to specific cytokine signals. mTORC1 supports this differentiation by enhancing glycolysis and lipid biosynthesis.^366,367^ mTORC1-deficient T cells have an impaired ability to differentiate.^366^ Similarly, T cells lacking the mTORC1 activator RHEB also fail to differentiate into Th1 and Th17 lineages.^368^ Conversely, deletion of the mTORC1 inhibitor TSC1 leads to hyperactivation of the pathway, resulting in increased Th1 and Th17 differentiation and multiorgan inflammation in mouse models.^369^ Furthermore, deficiencies in amino acid transporters for leucine LAT1^370^ and ASCT2 for glutamine^371^ disrupt Th1 and Th17 differentiation in an mTORC1-dependent manner. In CD8^+^ T cells, mTORC1 activity supports the generation of cytotoxic effector cells that eliminate infected or malignant cells. However, excessive mTORC1 signaling can impair the development of long-lived memory CD8^+^ T cells, ultimately weakening secondary immune responses.^372,373^

Tregs are immune cells that negatively control both cytotoxic effector CD8^+^ T cells and effector CD4^+^ T cells. mTORC1 activity has negative effects on Treg generation,^374,375^ while it is positively correlated with the inhibitory effects of Tregs on other T cells.^376^ Active mTORC1 promotes the conversion of Tregs to effector-like T cells and further impairs Treg stability and function.^369,377,378^ Signals from toll-like receptors, which stimulate Treg proliferation, also increase mTORC1 activity, glycolysis, and the expression of the glucose transporter Glut1.^377^

B cells undergo multiple steps of maturation before becoming plasma cells and acquiring antibody-secreting capacity, which is critical for defense against recurrent infections. B-cell development similarly depends on mTORC1, which is most active in early developmental stages and is gradually downregulated as cells mature.^379,380^ Deletion of RAPTOR prior to B-cell lineage specification results in a complete block in B-cell maturation.^379,380^

mTORC1 and mitochondria are tightly interconnected hubs that orchestrate immune cell metabolism and function and participate in the metabolic reprogramming of immune cells.^381,382^ Like in many other cell types, mTORC1 promotes mitochondrial biogenesis and function via PGC-1α, which enhances mitochondrial oxidative capacity in T cells and macrophages. mTORC1 also drives the metabolic switch to glycolysis in activated CD4^+^ and CD8^+^ T cells, supporting their proliferation and effector functions. Mitochondria play a crucial role in maintaining this metabolic switch by providing substrates for biosynthesis. Both mTORC1 and mTORC2 regulate the balance between effector and memory T-cell formation. Memory T cells rely more on mitochondrial metabolism, including fatty acid oxidation, to sustain their long-term survival and function. TORC1 promotes M1 macrophages (proinflammatory), which depend on glycolysis, while inhibiting M2 macrophages (anti-inflammatory), which rely on OXPHOS and mitochondrial metabolism. Additionally, mTORC1 regulates dendritic cell activation and antigen presentation by influencing mitochondrial dynamics and energy demands during the immune response.

Interestingly, B cells seem to have an mTORC1-independent mechanism for coordinating nutrient sensing and mitochondrial metabolism, which still depends on RAG-GTPases. Specifically, the RAGA/B complex constrains TFEB/TFE3 activity to prevent mitophagy dysregulation and maintain mitochondrial fitness, independent of canonical mTORC1 activation. RAGA/B deficiency results in reduced mitochondrial membrane potential and mitochondrial ROS, a phenotype distinct from that observed in *Raptor*-deficient B cells. This deficiency also leads to TFEB/TFE3 overactivation and abnormal mitophagy. TFEB/TFE3 deletion restores B-cell development and germinal center formation, whereas RAGA/B-deficient B cells fail to form germinal centers, which are crucial for B-cell proliferation and maturation, thus impacting humoral immunity.^383^

An emerging area of interest in immune regulation involves the cGAS–STING pathway, which connects mitochondrial stress to inflammatory signaling. Mitochondria, which retain ancestral bacterial motifs, are released into the cytosol or extracellular space as DAMPs upon cellular damage and are sensed by innate immune receptors to initiate inflammatory and antimicrobial defense responses.^384,385^ Upon stress, mitochondrial DNA leaks into the cytosol to activate the cGAS–STING pathway, while mitophagy is initiated to remove damaged mitochondria and suppress the inflammation triggered by mtDNA release.^386,387^ The mitophagy activator urolithin A, which indirectly inhibits mTOR through the PI3K/AKT/mTOR pathway, has been shown to reduce cytosolic mtDNA-dependent activation of the cGAS/STING pathway and subsequent inflammation.^387,388^ The cGAS–STING pathway also drives lysosome biogenesis via TFEB.^389^ Upon activation of cGAS–STING by cytoplasmic DNA, STING promotes lipidation of the autophagy-related protein GABARAP, which sequesters the FLCN–FNIP complex and blocks its GAP activity toward the RAGC/D GTPase. This selectively prevents mTORC1 from phosphorylating TFEB without affecting mTORC1’s catalytic activity toward its other substrates. Dephosphorylated TFEB then translocates to the nucleus to activate the transcription of lysosomal biogenesis genes, thereby enhancing lysosome formation and autophagic flux.

cGAS-STING activation drives the production of type I interferons (IFNs) as part of the innate immune response.^390^ Type I IFN-α signaling triggers mitochondrial oxidative stress and lysosomal alkalinization via mTOR activation, impairing autophagic flux in monocytes. This leads to cytosolic mtDNA accumulation, which activates the cGAS‒STING pathway, driving proinflammatory cytokine production. Inhibition of mTOR restores lysosomal acidification, rescues autophagy, and suppresses STING-dependent inflammation.^391^

Interleukin-10 (IL-10) is an anti-inflammatory cytokine that modulates macrophage glycolytic reprogramming by suppressing mTOR. Upon LPS stimulation, IL-10 prevents the metabolic shift from OXPHOS to glycolysis in macrophages. By inducing DNA damage inducible transcript 4, an mTOR inhibitor, IL-10 preserves the mitochondrial membrane potential, reduces ROS accumulation and facilitates mitophagy.^392^

These findings suggest that both the proinflammatory cGAS–STING–IFN axis and the anti-inflammatory cytokine IL-10 play crucial roles in mTOR-mediated mitochondrial inflammation and highlight mTOR inhibition as a potential therapeutic strategy for treating inflammatory diseases.

In summary, mTORC1 integrates both internal and external signals, including cues from the immune microenvironment, to orchestrate the development, differentiation, and functional specialization of immune cells and inflammatory responses.

### mTORC1 modulation of aging and lifespan

mTORC1 is recognized as a central driver of aging (Fig. 9). Inhibition of mTORC1 via genetic deletion of mTOR, RAPTOR, or S6K1 extends lifespan in yeast,^393^ worms,^394^ flies,^395^ and mice.^396^ Pharmacological inhibition with rapamycin similarly enhances both lifespan and health span in middle-aged mice, delaying age-related functional decline.^397^

mTORC1 inhibition promotes longevity through multiple pathways. A major mode of action is the restoration of autophagy, which clears damaged proteins and organelles and decreases with age. Autophagy activation increases lifespan in mice,^398^ but mTORC1 inhibition does not extend lifespan in autophagy-deficient worms,^399^ suggesting that without functional autophagy, the longevity benefit of mTORC1 inhibition is limited in this organism.

Another key mechanism involves the suppression of protein synthesis. Inhibition of mTORC1 reduces translation-related stress, limits the production of misfolded proteins, and alleviates oxidative damage. Dietary restriction mimics this effect by reducing mTORC1 activity and protein synthesis. In support of this, loss of S6K1 extends the lifespan of *C. elegans*^400^ and mice,^401^ likely by reducing the translation burden and improving proteostasis.

mTORC1 can regulate life span by modulating telomerase function. In telomerase-deficient mouse models, the mTORC1 pathway is activated, as manifested by increased phosphorylation of S6K1. Inhibition of the mTORC1 pathway in wild-type mice extends the median lifespan by 39%. However, in mice with telomerase deficiency, rapamycin reduces the median lifespan, indicating that mTORC1 inhibition may be detrimental to survival when telomeres are already shortened. In mice with dual deficiency of S6K1 and telomerase, rapamycin treatment also reduces lifespan. This finding suggests that inhibiting mTOR may be unfavorable for survival when telomeres are short, but mTORC1 inhibition can help delay aging when telomere length is normal.^402^ In addition, inhibition of mTORC1 by rapamycin facilitates the relocation of telomerase reverse transcriptase (TERT) to mitochondria. This translocation decreases the levels of mitochondrial superoxide and intracellular peroxides, thereby enhancing mitochondrial function and reducing ROS release.^403^

The accumulation of damaged mitochondria due to inefficient degradation and mtDNA instability is a significant hallmark of aging and age-associated diseases^404,405^ (Fig. 9). Because maintaining low mTOR activity contributes to longevity, low-dose rapamycin administration significantly extends the lifespan of mice with mtDNA depletion.^406^

Prolonged rapamycin administration facilitates the clearance of impaired mitochondria by increasing mitophagy, reduces the incidence of mtDNA deletion mutations, and has dose-dependent effects, with higher doses resulting in more significant outcomes.^407^

Chronic mTORC1 activation contributes to stem cell exhaustion and the accumulation of senescent cells.^408^ Senescent cells exhibit mTORC1 hyperactivity and secrete proinflammatory factors through translation programs controlled by mTORC1, thereby promoting systemic aging.^409,410^ Rapamycin mitigates this secretory phenotype, although whether it can reverse senescence or promote cell clearance remains unclear.

To date, the application of mTOR inhibitors to slow aging is limited by side effects such as insulin resistance and immunosuppression. However, these effects are typically associated with high-dose regimens used in cancer or transplant patients. Emerging evidence suggests that low or intermittent dosing can extend the lifespan of mice without adverse metabolic effects^411^ and may even enhance immune function in elderly humans.^412^ Finally, a recent study surveyed 333 adults who had used rapamycin off-label, primarily for health span and longevity purposes.^413^ Self-reported outcomes indicated enhanced health, cognitive function, and emotional well-being. Importantly, no significant adverse effects were reported, suggesting that low-dose, intermittent rapamycin use may be safe in healthy adults. While the findings are promising, further research, including controlled clinical trials, is necessary to establish the efficacy and safety of rapamycin for healthspan extension.

### mTOR in development

mTOR coordinates environmental cues with intrinsic developmental programs, ensuring that growth and differentiation occur at the right time and place. For example, mutations in *Drosophila* GATOR2 components suppress oocyte growth and differentiation^414^ and are required for ovary growth and female fertility.^415^ In *C. elegans*, NPRL2 and NPRL3 are required for postembryonic development^,^^416^ and NPRL3 deficiency in worm intestines triggers abnormal behavior and impaired growth.^417^

Lowering the activity of the mTOR signaling pathway causes human stem cells and lab-grown embryo-like structures (blastoids) to enter a temporary “resting” or dormant state. Human blastoids treated with mTOR inhibitors have limited proliferation, developmental progression, and capacity to attach to hormonally stimulated endometrial cells. They can also give rise to stem cell derivatives of the blastocyst lineages and can continue to differentiate under post-implantation culture conditions.^418^

Recently, the mTORC1 pathway was shown to play a role in facial phenotypic plasticity in mice.^419^ When pregnant mice are fed diets with low or high protein levels, the activity of mTORC1 in their developing fetuses changes. This affects how facial bones form by influencing the size of the groups of cells that make up the skeleton. As a result, the diet during pregnancy plays a role in determining the shape and structure of the offspring’s face.

Recent work in the axolotl (*Ambystoma mexicanum*), a highly regenerative urodele amphibian, has revealed a unique adaptation of the mTORC1 signaling pathway that underlies its exceptional regenerative capabilities by regulating translational control.^420^ Following injury, axolotls rapidly upregulate protein synthesis from preexisting mRNAs, particularly those encoding ribosomal components and antioxidants, via a hypersensitive mTOR kinase variant. This response is absent in nonregenerative injury mouse models. Notably, the axolotl mTOR (axmTOR) protein has undergone unique evolutionary sequence expansions that are not found in mammals. These alterations confer heightened sensitivity to extracellular signals, enabling mTORC1 activation even in response to low amino acid levels at wound sites. Furthermore, a portion of axmTOR remains constitutively localized to lysosomes, priming the pathway for rapid activation upon injury. This hypersensitive mTOR system allows axolotls to repurpose a classical stress response pathway into a robust pro-regenerative signal, suggesting that species-specific mTOR tuning may be a critical determinant of regenerative capacity in vertebrates.

Together, these studies highlight the remarkable versatility of the mTORC1 signaling pathway across species and developmental stages. Understanding how mTORC1 is fine-tuned in diverse biological contexts may open new avenues for regenerative medicine, reproductive health, and developmental biology.

## mTOR signaling in human diseases

Aberrant mTOR signaling is commonly associated with a wide range of diseases, including cancer, diabetes, obesity, various neurological disorders and other pathologies. This dysregulation can result from several mechanisms: (1) activating mutations within mTOR itself, mTOR complexes or their upstream regulatory proteins; (2) elevated expression levels of mTOR complex components; (3) inactivation or loss of function of negative regulators within the mTOR signaling pathway; and (4) changes in mTOR activity due to metabolic stress and mitochondrial dysfunction.

Although mTOR activation is often observed in the majority of disorders, it is not universally upregulated in all diseases. For example, in cancer-associated cachexia, mTORC1 signaling is downregulated in skeletal muscles. In advanced stages of nonalcoholic fatty liver disease, including cirrhosis, mTORC1 activity may also decline due to mitochondrial deregulation.^421^

In an attempt to classify different mTOR-related diseases, several suggestions for the collective names of various disorders have recently been proposed. The term mTORopathy is used in the context of neurological diseases, especially epileptic encephalopathies and cortical malformations such as tuberous sclerosis due to mutations in *TSC1* or *TSC2*; focal cortical dysplasia, often caused by somatic mutations in *MTOR*, *PIK3CA*, or *AKT3*; hemimegalencephaly; brain malformation associated with mutations that hyperactivate mTOR signaling; and DEPDC5-related epilepsy.^422^

GATORopathies refer more specifically to disorders, other than cancer, caused by mutations in the genes in the GATOR1 complex.^423^ GATORopathies are mostly associated with familial focal epilepsy with variable foci, nonlesional focal epilepsies, and malformations of cortical development. Recently, mutations in DEPDC5 were also observed in autism spectrum disorders^,^^424^ as were mutations in NPRL3 in Parkinson’s disease,^425^ suggesting a broader neurological impact of GATOR1 dysfunction.

RAGopathies are a broader set of disorders resulting from mutations in the RAG GTPases,^426^ such as mutations of RAGA in cataracts,^427^ RAGC^428^ in dilated cardiomyopathy, RAGD in kidney tubulopathy and cardiomyopathy^429,430^ and RAGC in follicular lymphoma.^431^

The upregulation of mTOR in diseases has profound effects on mitochondria (Fig. 9). Conversely, mitochondrial dysfunction can also influence mTOR signaling, creating a bidirectional relationship that plays a critical role in many pathological conditions. Thus, overactive mTORC1 inhibits mitophagy, leading to the accumulation of dysfunctional mitochondria, which contributes to increased ROS production and reduced cellular energy efficiency. On the other hand, mitochondrial dysfunction in cancers forces cells to rely on glycolysis (the Warburg effect) for energy production, which is often accompanied by hyperactivation of mTORC1 to support anabolic needs.

### mTOR signaling in cancer

mTOR signaling is altered in nearly 30% of cancers^432^ (Fig. 9). The activating mutations within the kinase domain of mTOR can directly increase mTOR signaling. For example, approximately 33 mutations localized in six distinct regions of the mTOR gene C-terminal region have been identified in human tumors.^433^ These mutations promote mTOR kinase activity via the inhibition of DEPTOR binding in lung carcinoma, stomach cancer, endometroid carcinoma, colorectal cancer, melanoma and renal cell carcinoma. Owing to these mTOR mutations, various cell lines have become sensitive to mTOR inhibitors both in vitro and in murine models.^433^

RICTOR amplification leads to a poor prognosis and short survival in patients with squamous cell lung carcinoma.^434^ RICTOR is also robustly expressed in HER2-amplified breast carcinoma.^435^ In addition, its upregulation is associated with metastasis and drug resistance in triple-negative breast cancer and promotes the motility and proliferation of glioma cells.^436^

Hyperactivation of mTOR signaling can also be the result of mutations in upstream oncogenes and tumor suppressor genes. Mutations, amplifications, and overexpression of PIK3CA, KRAS, AKT, IGFR and EGFR upstream of mTORC1 are very common in human malignancies.^437^ Additionally, the loss of function of p53, PTEN, and TSC1/2 contributes to mTOR signaling activation in a variety of tumors. For example, p53 depletion can enhance cell proliferation and survival, disrupt apoptosis and increase genomic instability by altering the expression of REDD1, an mTOR suppressor.^438^ In breast and endometrial cancers, alterations in PTEN expression increase the sensitivity of cells to mTOR inhibitors.^439^ Mutations in TSC1/2 identified in renal, liver, bladder, pancreatic and urothelial cancers are associated with tumorigenesis.^440,441^

Several components of the nutrient-sensing input to mTORC1 have also been implicated in cancer progression. For example, RHEB1 upregulation, which increases mTORC1 activity, leads to worse median survival in acute myeloid leukemia patients.^442^ RAGC^431^ is mutated at a high frequency in follicular lymphoma. Causative damage caused by Birt-Hogg-Dube hereditary cancer syndrome is related to folliculin mutations.^443^ Mutations in NPRL3 and DEPDC5 were found in glioblastoma,^47^ and DEPDC5 alternations were detected in colorectal cancer.^444^ NPRL2 has the most recurrent cancer-associated mutations in lung cancer, hepatocellular carcinoma, glioblastoma, and cancers of the ovary, breast, kidney, and colon.^76^ Additionally, NPRL2 loss has been associated with decreased sensitivity to the chemotherapeutic agent cisplatin.^188,445^

Healthy cells primarily carry out OXPHOS in mitochondria to generate ATP. Cancer cells tend to produce energy through glycolysis rather than OXPHOS even when oxygen is sufficient.^446^ Under hypoxic conditions, cells adjust their metabolism through the mTORC1 signaling pathway, promoting a shift from OXPHOS to glycolysis to adapt to the low-oxygen environment and sustain the energy supply during cancer cell proliferation.^21,28^ Dysregulated mitochondrial dynamics, abnormal mitophagy, mtDNA and TCA cycle enzyme mutations are implicated in cancer invasiveness, metastasis, progression and anticancer drug resistance^447^ (Fig. 9).

Mitochondrial fragmentation is common in various cancer types. Targeting mitochondrial fragmentation via the mTOR-MFN2 signaling axis may be a promising strategy to limit tumor growth. Indeed, MFN2 overexpression enhances mitochondrial fusion, reduces ROS generation, activates AMPK, inhibits mTOR, and promotes autophagy, ultimately suppressing ovarian cancer progression.^448^ Mitochondrial fragmentation is also induced by the hypoxic tumor microenvironment, which leads to mTOR signaling activation. One consequence of this is that natural killer (NK) cells, which are crucial for tumor immunosurveillance, are unable to perform their normal antitumor functions, leading to evasion of immune surveillance and accelerating tumorigenesis.^449^ Restoring the antitumor capacity of NK cells can be achieved by inhibiting mitochondrial fragmentation.^449^

Mutations in metabolic enzymes, changes in cell signaling, and a hypoxic environment in cancer cells lead to altered metabolic pathways, driving mitochondria to produce oncometabolites such as lactate, fumarate, succinate and 2-hydroxyglutarate (2-HG).^450^ Although these compounds are normal intermediates in metabolism, their pathological accumulation disrupts cellular homeostasis.

mTOR promotes the accumulation of oncometabolites through mitochondrial biogenesis, glutamine metabolism, and HIF-1α signaling. For example, inactivation of *Tsc1* leads to mTORC1 upregulation, fumarate accumulation and mTOR-dependent downregulation of the TCA enzyme fumarate hydratase (FH). Treatment with rapamycin reduces fumarate levels, affecting renal cancer progression in mice.^451^ mTORC1 upregulation is correlated with FH downregulation and fumarate accumulation in human renal cell carcinoma.^451,452^

Activation of mTORC1 by tumor-derived lactate leads to TFEB retention in the cytoplasm, which in turn decreases the expression of the macrophage-specific vacuolar ATPase subunit ATP6V0d2, which is required for lysosomal-mediated degradation of hypoxia-induced factor 2α (HIF-2α). In turn, HIF-2α promotes expression of protumoral genes in macrophages.^453^

In acute myeloid leukemia and gliomas, cancer-associated mutations in the TCA enzymes IDH1 and IDH2 lead to the production of 2HG from α-KG.^454^ This abnormal generation of 2HG results in mTOR activation through the inhibition of KDM4A, an α-KG-dependent lysine demethylase that stabilizes the mTOR suppressor DEPTOR. KDM4A inhibition decreases DEPTOR stability, promoting mTOR activation.

Another important axis of tumor metabolism is the mTOR-related and mitochondria-driven synthesis of purines, pyrimidines, and amino acids. The excess production of these molecules fuels the uncontrolled proliferation of cancer cells.

Aspartate, especially under conditions of impaired mitochondrial metabolism, is an important factor for tumor growth. The conversion of aspartate to asparagine is crucial for the proliferation of cancer cells.^452,455^ Inhibition of the ETC leads to aspartate depletion, triggering ATF4 upregulation and increasing the transcription of genes related to amino acid transport and synthesis. Asparagine deficiency reduces mTORC1 activity,^455^ whereas aspartate or adenylate supplementation restores mTORC1 activity.^452^

The byproducts of tyrosine catabolism serve as intermediates in the TCA cycle, and dysregulation of this process contributes to the progression of hepatocellular carcinoma.^456^ Deficiencies in the tyrosine metabolic enzyme 4-hydroxyphenylpyruvate dioxygenase (HPD) lead to mTOR activation, promoting the accumulation of TCA cycle intermediates, such as citrate, α-KG, succinate, fumarate, and malate. Inhibition of mTOR or p70S6 kinase helps control the progression of liver cancer in HPD-deficient patients.^456^

Chronic lymphocytic leukemia (CLL) patients with high proliferation features have upregulated OXPHOS and the mTOR pathway.^457^ Venetoclax, a BCL-2 inhibitor, is used in CLL treatment. In the tumor microenvironment, CLL cells are stimulated by various signals, including the activation of CD40 signaling, which enhances OXPHOS and glycolysis, increases the expression of antiapoptotic proteins, and is associated with venetoclax resistance.^458,459^ AZD8055, which suppresses both mTORC1 and mTORC2, effectively reduces mitochondrial mass and respiratory activity and downregulates the expression of antiapoptotic proteins induced by CD40 stimulation to alleviate venetoclax resistance.

The biguanides metformin and phenformin inhibit mitochondrial electron transport chain complex I. Inhibition of mTORC1 restored autophagy in cells treated with these drugs, helping cancer cells bypass the blockage of phosphorylation of the E1 subunit α of PDH and making it a potential therapeutic target in tumor cells that are resistant to mTOR-mediated therapies.^460^

### mTOR signaling in neurological disorders

mTOR signaling is involved in regulating neurological processes^461^ and psychiatric and autism spectrum disorders.^462,463^ Deletions of RAPTOR or RICTOR decrease neuron size, impair oligodendrocyte differentiation and myelination in the central nervous system and are associated with early death, thus affecting the underlying functions of mTORC1 and mTORC2 in the development of the brain.^464^

Hyperactivation of mTORC1, which leads to impaired mitochondrial function, is observed in many neurodegenerative diseases (Fig. 9). For example, one of the main characteristics of Parkinson’s disease is defective PINK/PARKIN-mediated mitophagy.^465^ Treatment with rapamycin enhances mitophagy, prevents mitochondrial dysfunction, and protects against neuronal apoptosis, safeguarding vascular dementia mice from brain injury and cognitive impairment.^466^

In Alzheimer’s disease (AD), mTORC1 overactivation and mitochondrial dysfunction contribute to neurodegeneration by impairing autophagy and mitophagy, increasing ROS production, reducing glucose utilization, disrupting energy metabolism, and promoting the accumulation of amyloid-beta oligomers as well as hyperphosphorylated and aggregated forms of pathological tau protein^467^ (Fig. 9). The induction of synaptic activity has been shown to protect against AD by promoting autophagic flux and the lysosomal degradation of tau.^468^

Hyperactivation of mTORC1 reduces the autophagic clearance of amyloid-beta, promoting its accumulation in the brain. Amyloid-beta directly interacts with mitochondrial membranes, impairing ETC function, increasing ROS production, and blocking communication between lysosomes and mitochondria in vivo.^469^ mTORC1 dysregulation exacerbates mitochondrial vulnerability to amyloid-beta, creating a feedback loop that amplifies neurodegeneration. Amyloid-beta oligomers, which are precursors to amyloid plaques in the Alzheimer’s disease brain, stimulate mTORC1 kinase activity at the plasma membrane but not at lysosomes and block nutrient-induced mitochondrial activity (NiMA) through a mechanism dependent on tau.^470^

Niemann-Pick type C disease is a lethal neurodegenerative disorder caused by mutations in the cholesterol exporter NPC1, leading to aberrant cholesterol accumulation in lysosomes, hyperactivation of mTORC1 signaling, and compromised mitochondrial function (Fig. 9). Treatment of NPC1-null cells with the mTOR inhibitor Torin1 or downregulation of mTORC1 activity via knockdown of LAMTOR5 restores impaired lysosomal and mitochondrial functions in these cells.^471^

In patients with TSC1/2 loss, mTORC1 hyperactivation leads to severe epileptic seizures, which are reduced after rapamycin treatment.^472^ In addition, epileptic disorders can also be caused by mutations within the KICSTOR and GATOR1 complexes.^76^

In recent years, the GATOR1 complex has been shown to play an important role in the pathogenesis of focal epilepsies, with DEPDC5 exhibiting a higher mutation frequency (approximately 85% of reported cases) than NPRL2 (5%) and NPRL3 (10%). Among these GATOR1 variants, most are loss-of-function mutations, suggesting that GATOR1 genes, especially DEPDC5, should be prioritized in clinical genetic screening for focal epilepsy patients.^473^ Clinical studies have also identified loss-of-function mutations in GATOR1 components, especially DEPDC5 and NPRL3, in FCD type IIa. These mutations are associated with impaired autophagic flux, as indicated by the accumulation of p62 in autophagosomes in approximately half of the biopsy samples.^474^ A retrospective study of 50 children with GATOR1 variant-related epilepsy revealed that the majority of patients were resistant to antiseizure medications and presented altered brain structures.^475^ Epilepsy surgery significantly improved seizure outcomes, indicating that surgery might be a more effective therapeutic approach for patients with GATOR1 variants.

Dietary restriction, specifically acute fasting, has been shown to decrease seizure susceptibility, which is linked to a reduction in mTORC1 activity. Interestingly, in neuron-specific DEPDC5 knockout mouse models, increased susceptibility to seizures and seizure-induced death was observed, suggesting the critical role of DEPDC5 in modulating seizure susceptibility following acute fasting.^476^

A recent study identified three novel NPRL3 loss-of-function mutations in families with focal epilepsy.^477^ These mutations disrupt NPRL3 protein stability, impairing the ability of the GATOR1 complex to inhibit mTORC1, leading to increased phosphorylation of S6K1.

### mTOR signaling in cardiovascular diseases

Dysregulation of mTORC1, which leads to mitochondrial dysfunction, contributes to atherosclerosis, cardiomyopathy, ischemia–reperfusion injury, hypertension, and hypertrophy. For example, in high-protein diet-induced atherosclerotic models, amino acid starvation-induced mTORC1 inhibition promotes mitophagy, whereas leucine supplementation activates mTORC1 and suppresses mitophagy, causing macrophage apoptosis. The macrophage mTORC1-mitophagy axis should be considered an important target in high-protein diet-related cardiovascular risk.^478^

PTEN, an upstream negative regulator of mTOR, has been shown to be regulated by YAP, the downstream effector of the Hippo pathway.^479^ Activation of this pathway suppresses the expression of mitochondrial genes, contributing to mitochondrial damage and the progression of cardiomyopathy.^480^

Barth syndrome (BTHS), characterized by dilated cardiomyopathy, is an X-linked genetic disease caused by *TAFAZZIN* gene mutation. The transacylase encoded by *TAFAZZIN* is crucial for the remodeling of cardiolipin phospholipids, which are essential for maintaining mitochondrial structure and function. *TAFAZZIN* deficiency leads to mTORC1 hyperactivation, reduced mature cardiolipin levels, mitochondrial dysfunction, and defective mitophagy (Fig. 9). Treatment with rapamycin in *TAFAZZIN*-knockdown mice restores mitophagy, improves impaired mitochondrial morphology and respiratory function, reduces ROS, and ameliorates BTHS-related cardiomyopathy.^481^ Therefore, targeting the mTORC1 pathway could serve as a novel therapeutic approach for BTHS, a disease that currently lacks effective treatment.

On the other hand, mTORC1 activation has a protective effect in cardiac reperfusion injury. Reduced phosphorylation of TSC2 enhances mTORC1 activity, followed by an increased mitochondrial oxygen consumption rate and a metabolic shift from fatty acid oxidation to glycolysis, which helps attenuate mitochondrial damage during ischemia and reperfusion.^482^

### mTOR signaling in diabetes

The abnormal activation of mTORC1 and impairment of the TCA cycle jointly contribute to the dysfunction of diabetic β-cells, including reduced insulin secretion and increased apoptosis (Fig. 9). Upregulated mTORC1 contributes to the inhibition of pyruvate dehydrogenase, further reducing the entry of pyruvate into the TCA cycle. By restricting glycolysis during hyperglycemia, these metabolic changes may be prevented, potentially slowing the progression of diabetes.^483^

In diabetic nephropathy, high glucose conditions induce the upregulation of YY1, promoting the assembly of the mTOR‒YY1 heterodimer. This complex then binds to the PGC-1α promoter and suppresses PGC-1α activity. This inhibition leads to mitochondrial dysfunction, which in turn promotes renal fibrosis in early diabetic nephropathy. Treatment with rapamycin reverses this process by downregulating YY1 expression.^287^

During the progression of diabetic retinopathy, the expression of the mitophagy proteins PARKIN and PINK1 is upregulated, and mTOR signaling is activated. Overexpression of Wnt inhibitory factor 1 (WIF1) suppresses the Wnt/β-catenin signaling pathway, reduces the activity of the AMPK/mTOR signaling pathway, and improves the mitochondrial function caused by diabetic retinopathy.^484^

### mTOR signaling in other diseases

Lymphangioleiomyomatosis (LAM) is a rare multisystem disorder that predominantly affects women and is characterized by progressive cystic lung destruction, renal angiomyolipomas (AMLs), and lymphangioleiomyomas.^485^ Its hallmark is the proliferation of abnormal smooth muscle–like perivascular epithelioid cells (LAM cells) within the lungs and lymphatics. LAM occurs in two forms: TSC-associated LAM, caused by germline mutations in TSC1 or TSC2, and sporadic LAM, which most often results from somatic loss-of-function mutations in TSC2. TSC1 or TSC2 germline mutations and sporadic LAM are often caused by somatic loss-of-function mutations in TSC2. Both forms share dysregulation of the mTORC1 pathway due to loss of TSC gene function, leading to uncontrolled cell growth and infiltration. In recent years, clinical studies have demonstrated that sirolimus and everolimus can stabilize lung function, shrink AMLs, reduce lymphatic involvement, improve quality of life, and establish mTORC1 inhibition as the current standard of care for LAM patients.

The m.3243 A > G mutation is the most common pathogenic mtDNA mutation in humans, which causes severe conditions, known as MELAS (myopathy, lactic acidosis and stroke episodes) and less severe MIDD (maternally inherited diabetes and deafness). This mutation results in mitochondrial dysfunction and elevated ROS and is associated with constitutive activation of the PI3K-AKT-mTORC1 pathway^486^ and suppressed autophagy and mitophagy (Fig. 9). Inhibition of mTORC1 with rapamycin in patient fibroblasts reduced the mutation load, rescued mitochondrial bioenergetic function, and reduced glucose dependence. Effective mitophagy triggered by mTORC1 inhibition is necessary to eliminate the m.3243 A > G mtDNA mutation.^487^ mTOR hyperactivation and suppressed autophagy are also observed in Down syndrome disease, where restoration of mitophagy and autophagic flux by blocking mTOR activity with the dual mTORC1/mTORC2 inhibitor AZD8055 has been proposed as a promising therapeutic strategy.^488^

Nonalcoholic fatty liver disease (NAFLD) and nonalcoholic steatohepatitis (NASH) are metabolic disorders characterized by significant lipid metabolism abnormalities. Mitochondrial dysfunction is implicated in the progression of NAFLD. Neddylation, a draggable and reversible posttranslational modification, is increased in different liver diseases, including NAFLD.^489^ Neddylation inhibition reduces oxidative stress, lipid peroxidation, fatty acid oxidation, and OXPHOS in preclinical NAFLD models. These effects are linked to mTOR suppression via DEPTOR accumulation.^489^ Another role of mTOR has been identified through its connection with endonuclease G, which promotes NAFLD via mTORC2 upregulation and ER stress.^276^

Taken together, these findings indicate that mTOR signaling has a profound impact across a diverse spectrum of human diseases. While hyperactivation of mTORC1 is a hallmark of many malignancies, metabolic disorders, and neurological pathologies, its role is not universally pro-growth or pro-survival. In fact, under certain pathological conditions, such as cancer-associated cachexia, cirrhosis, and advanced mitochondrial dysfunction, mTOR activity may decline, revealing the context-dependent complexity. As such, a growing body of evidence suggests that mTOR and mitochondria function as interdependent hubs, coordinating metabolic responses, cell fate decisions, and inflammatory signaling. This intricate crosstalk becomes especially relevant in the context of chronic diseases, where metabolic stress and immune dysfunction converge. Understanding how mTOR and mitochondria coregulate cellular fate across tissues and disease states will be essential for designing targeted interventions that restore homeostasis without disrupting essential physiological functions.

## Modulating mtor activities with mtor inhibitors

**Fig. 10:**
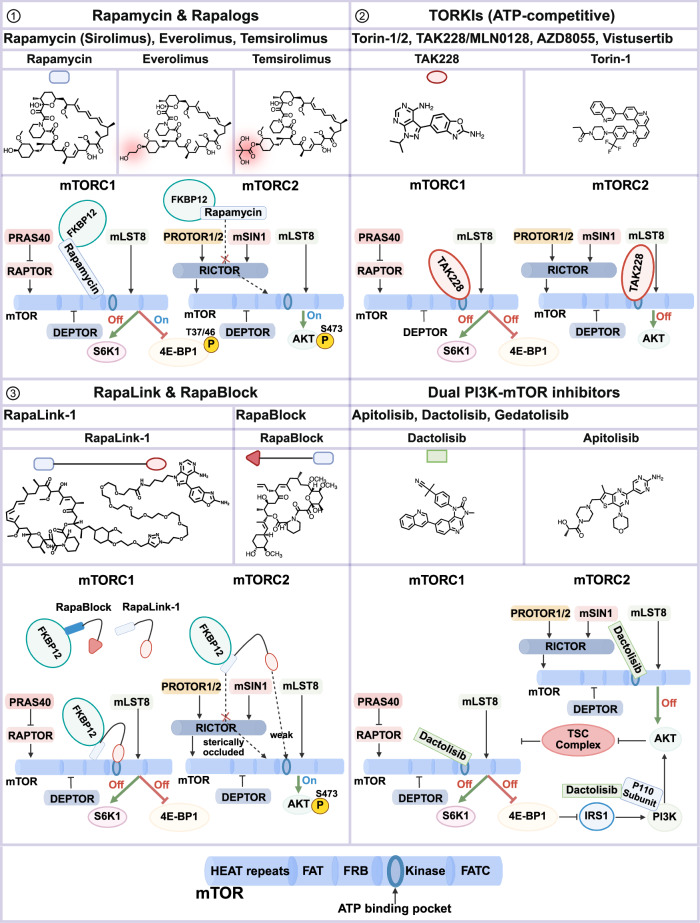
Classification and mechanisms of action of mTOR inhibitors. The names of different generations of mTOR inhibitors are indicated at the top layer and marked by a corresponding number. The second layer lists representative drugs commonly utilized within each inhibitor category. The third layer shows the chemical structures of selected representative compounds. The final layer illustrates the distinct molecular mechanisms of inhibitor action, depicting interactions with mTORC1 and mTORC2 complexes and highlighting specific binding sites, inhibition targets, and downstream effects. The domain composition of mTOR is shown at the bottom. The following classes of mTOR inhibitors are represented: (1) Rapamycin/sirolimus (blue rectangle) and rapalogs (first-generation inhibitors). Red shading indicates key structural variations of everolimus and temsirolimus relative to rapamycin. Sirolimus and everolimus are orally administered; everolimus has improved pharmacokinetics, better oral bioavailability and more predictable absorption. Sirolimus is primarily used in organ transplantation to prevent rejection, whereas everolimus is more commonly employed in oncology (advanced renal cell carcinoma, hormone receptor-positive breast cancer and pancreatic neuroendocrine tumors). Temsirolimus, a water-soluble prodrug of rapamycin that is administered intravenously, is used for the treatment of advanced renal cell carcinoma. (2) ATP-competitive TOR kinase inhibitors (TORKIs, second-generation inhibitors). The chemical structures of TAK228/MLN0128 (pink oval) and Torin-1 are shown. (3) RapaLink-1 (third-generation) and RapaBlock. In RapaLink-1, rapamycin (blue rectangle) is connected to TORKI TAK228/MLN0128 (pink oval) by a linker composed of eight ethylene glycol units (-(CH_2_CH_2_O)_8_-). Dual PI3K‒mTOR inhibitors are illustrated in the lower right box. Phosphorylation by mTORC1 activates S6K1 (green arrow) but inhibits 4E-BP1 (red inhibition sign), whereas phosphorylation by mTORC2 activates AKT (green arrow). First-generation inhibitors block the phosphorylation of S6K1 by mTORC1 but not T37/46 of 4E-BP1 or S473 of AKT by mTORC2. Second-generation inhibitors block all three substrates, whereas RapaLink-1 selectively blocks mTORC1 targets. FKBP12 binding is necessary for the activity of rapalogs and RapaLink-1 but not for TORKIs. Compared with RapaLink-1, RapaBlock binds FKBP12 with higher affinity, preventing FKBP12 from interacting with the mTORC1-RapaLink-1 complex

Given the central role of mTOR in numerous pathological conditions, it is important to design therapeutic strategies that can support appropriate pathway activity in health and disease. A key aspect of this effort lies in the development of various mTOR inhibitors, along with strategies to increase their efficacy through drug combinations (Fig. 10). This section outlines the core mechanisms of these inhibitors and their interactions, while more specific data, such as clinical trial results and IC₅₀ values in models of cancer and other disorders, are covered in detail in several dedicated reviews.^490–495^

Rapamycin, which is used clinically under the generic name sirolimus or marketed under the brand name rapamune by Pfizer and Novartis, was the first drug developed to inhibit mTOR, followed by several analogs that constitute first-generation mTOR inhibitors (Fig. 10). These rapalogs differ in their chemical modifications, which determine their pharmacokinetics and routes of administration.

Although rapalogs have achieved great success in the field of organ transplantation, where their immunosuppressive properties have proven highly effective in preventing graft rejection, their application in oncology has been far less successful. Clinical trials in cancer patients with rapalogs, particularly as monotherapies, revealed only modest efficacy, with limited and inconsistent therapeutic responses across tumor types.

This suboptimal effectiveness reflects not only the complexity of mTOR signaling in cancer but also specific mechanistic limitations inherent to the rapalog mechanism of action. First, the 4E-BP signaling branch downstream of mTORC1 remains largely unresponsive to rapamycin. Second, selective inhibition of mTORC1, without concurrent suppression of mTORC2, triggers compensatory activation of the PI3K–AKT pathway via an S6K1–IRS1 feedback loop, promoting cell survival rather than suppression. Additionally, rapamycin has been shown to promote proliferation within hypoxic, poorly vascularized tumor regions by stimulating lysosomal degradation of extracellular proteins.^496,497^

In response to these limitations, a second generation of mTOR inhibitors, also referred to as pan-mTORC1/2 inhibitors or TORKIs, was developed to more comprehensively inhibit mTOR signaling (Fig. 10). These compounds act as ATP-competitive inhibitors, effectively blocking both mTORC1 and mTORC2 by directly targeting the mTOR kinase domain.^498,499^ Examples include Torin1,^499^ AZD2014 (vistusertib),^500^ and AZD8055.^501^ These inhibitors exhibit greater selectivity for mTOR than for PI3K and other PIKK family kinases. A more advanced generation of TORKIs, such as Torin2, also inhibits the DNA damage-sensing kinases ATM, ATR, and DNA-PK while retaining limited activity against PI3K.^502^ TORKIs have shown superior ability to sensitize cancer cells to DNA-damaging agents by disrupting DNA damage response pathways and interfering with cell cycle checkpoint control, a capability that rapalogs notably lacking.^503^

Despite these advances, the use of first- and second-generation mTOR inhibitors has been associated with a range of side effects such as insulin resistance, hyperglycemia, impaired wound healing, hypertension, and various metabolic, renal, and pulmonary complications. A major contributor to this is the nonselective inhibition of both mTORC1 and mTORC2 during extended treatment. Even if intermittent dosing of rapamycin extends the lifespan of mice and has a milder impact on glucose homeostasis compared to continuous administration, without triggering the glucose intolerance,^411^ a long-term administration of rapamycin eventually inhibits not only mTORC1 but also mTORC2 and may lead to hepatic insulin resistance.^345,504^ Acute mTOR inhibition by AZD8055 also leads to insulin resistance and glucose intolerance.^505^

Building on the quest for selectivity and improved efficacy, a major breakthrough came with the development of third-generation mTOR inhibitors (Fig. 10). These novel agents, exemplified by RapaLink-1, represent a significant leap forward in targeted therapy. RapaLink-1 is a bitopic chimeric molecule that simultaneously engages both the FRB domain and ATP-binding site of mTOR.^506^ RapaLink-1 demonstrates improved potency, durable intracellular retention, reduced toxicity and efficient suppression of downstream targets such as 4E-BP1. Designed by linking an FRB-binding moiety to a TORKI structure, RapaLink-1 is able to overcome resistance in cancer cells that harbor mutations in either the FRB or kinase domains of mTOR. It can also cross the blood‒brain barrier, leading to potent antitumor activity in glioblastoma xenograft mouse models with minimal systemic toxicity.^507^

An important complementary tool in the RapaLink-1 system is RapaBlock (Fig. 10), which selectively binds to FKBP12 and prevents its association with rapamycin.^508^ RapaBlock is not considered a third-generation mTOR inhibitor, as it lacks the dual-site targeting and therapeutic activity characteristic of RapaLink-1. Due to the significantly greater stability of the FKBP12–rapamycin complex than the FRB–rapamycin interaction, RapaBlock effectively inhibits the assembly of the near-irreversible FKBP12–rapamycin–FRB tripartite complex.^509^ As a result, TORC1 remains predominantly active and unbound. Importantly, RapaBlock cannot cross the blood‒brain barrier, enabling the selective activity of RapaLink-1 in the central nervous system. In the brain, RapaLink-1 forms a stable ternary complex with mTORC1 and FKBP12, effectively silencing mTORC1 activity. This tissue-selective targeting strategy, when RapaLink-1 is combined with RapaBlock, has been shown to prolong survival in mouse models of glioblastoma, highlighting its potential for treating brain tumors while minimizing systemic toxicity.

While third-generation mTOR inhibitors represent major advances in specificity and potency, alternative approaches have also been pursued to increase therapeutic efficacy and overcome pathway redundancies. In particular, dual PI3K–mTOR inhibitors such as NVP-BEZ235 (dactolisib),^510^ GDC-0980 (apitolisib),^511^ and PKI-587 (gingatolisib)^512^ are ATP-competitive compounds designed to block both PI3K and mTOR signaling (Fig. 10). By simultaneously targeting these two nodes, they mitigate the compensatory PI3K–AKT activation often seen with selective mTORC1 inhibition. Dual inhibitors are especially effective in sensitizing cancer cells to genotoxic and replication stress, further expanding the arsenal of strategies for targeting the PI3K–mTOR axis in oncology.

mTOR activity can also be modulated indirectly through the actions of several metabolic drugs on mitochondria. For example, metformin, a widely used antidiabetic drug, inhibits mitochondrial complex I, leading to AMPK activation and downstream inhibition of mTORC1.^513–515^ Similarly, resveratrol^516^ and AICAR^517^ activate AMPK and promote mitochondrial biogenesis while suppressing mTOR signaling. The novel mitochondrial inhibitor IACS-010759 (OPi) suppresses mTOR by enhancing the activity of the AMPK pathway and inhibiting OXPHOS.^518^ Drugs such as bezafibrate support fatty acid oxidation and mitochondrial health while also downregulating mTOR signaling.^519^ These mitochondria-targeting agents offer promising avenues for modulating mTOR activity in a context-dependent manner and may complement direct mTOR inhibition strategies, particularly in diseases where metabolic dysfunction and mitochondrial decline are key pathological features, such as neurodegeneration, aging, and certain cancers. On the other hand, pharmacological modulation of mTOR can substantially influence mitochondrial health by promoting mitophagy, enhancing mitochondrial biogenesis, and reducing oxidative stress.^488^ For example, treatment with rapamycin demonstrates mitochondrial protection in models of Parkinson’s and Alzheimer’s diseases.^520,521^ Compared to rapalogs, more potent mitophagy and metabolic reprogramming, as well as effects on mitochondrial dynamics and biogenesis, were observed upon treatment with second-generation mTOR inhibitors.^522^

Current therapeutic strategies increasingly favor combination treatments, where mTOR inhibitors are paired with genotoxic agents. For example, targeting mTORC1 with rapamycin and AKT with MK2206 has been shown to increase radiation sensitivity in breast and non-small cell lung cancer models.^523^ Similarly, the dual PI3K‒mTOR inhibitor NVP-BEZ235 improved the response of glioblastoma cells to ionizing radiation.^524^ Finally, mTOR represents an attractive target for enhancing sensitivity to cisplatin, one of the mainstream chemotherapeutic agents.^188^

An emerging strategy in cancer treatment involves combining mTOR inhibitors with hormonal therapy to stop the growth of hormone-sensitive cancers.^525^ These immunomodulatory effects provide a rationale for the synergistic efficacy when paired with immune checkpoint inhibitors. Combinations of everolimus with antibodies against the key immune checkpoint proteins programmed cell death protein 1 (PD-1) and programmed death-ligand 1 (PD-L1) are under investigation to overcome the immunosuppressive tumor microenvironment and improve therapeutic outcomes in renal cell carcinoma.^526^

Recently, several combination therapies have been developed that, rather than targeting mTOR directly, focus on its upstream regulators in conjunction with immunotherapy. These strategies aim to modulate tumor growth and enhance immune responses by influencing signaling pathways that intersect with mTOR signaling. For example, combining PI3K inhibitors with antibodies against the key immune checkpoint proteins PD-1/PD-L1 has improved antitumor responses in preclinical models by augmenting T-cell-mediated tumor cell killing and reducing immunosuppressive cell populations within the tumor microenvironment.^527,528^ AKT inhibitors can also increase the effectiveness of immunotherapies by promoting apoptosis in tumor cells and improving T-cell responses.^529^ Finally, immediate upstream regulators of mTOR can also serve as effective targets in combination therapies. Recent studies have demonstrated that restoring NPRL2 expression via cationic lipid nanoparticle-mediated gene delivery in humanized mouse models represents a promising therapeutic strategy.^530^ Restoration of NPRL2 expression in KRAS/STK11-mutant anti-PD1-resistant NSCLC models induced DNA damage, cell cycle arrest, and apoptosis while also eliciting a robust antitumor immune response. NPRL2 gene therapy synergized with immune checkpoint blockade enhanced immune-mediated tumor suppression.

Together, the expanding arsenal of mTOR inhibitors, which are capable of targeting different branches of mTOR signaling, either alone or in combination with genotoxic agents or immunotherapies, represents a powerful toolkit for the treatment of cancer and other diseases.

## Conclusions and perspectives

Over the past five decades, the discovery of rapamycin and the subsequent characterization of the TOR pathway have revolutionized our understanding of cellular growth, metabolism, and aging. From the initial identification of rapamycin as an antifungal compound to the current recognition of mTOR as a central modulator of cellular physiology, the trajectory of mTOR research represents a compelling story of scientific progress. Today, the mTOR complexes serve as critical hubs that integrate many environmental cues (nutrients, energy status, oxygen levels, growth factors and DNA damage) to orchestrate cellular and organismal homeostasis.

Mechanistically, significant advances have been made in delineating the regulation and spatial dynamics of mTORC1 signaling, particularly its localization to lysosomes, and evidence of nonlysosomal activity at the Golgi, ER, and even within the nucleus has emerged. Nevertheless, how mTORC1 and mTORC2 interact with their substrates at different cellular locations and how compartment-specific signaling contributes to distinct physiological outcomes are still unclear. It is also poorly understood how mTORC1/2 complexes are targeted to specific subcellular locations and whether there are specific signatures (for example, posttranslational modifications or scaffolding interactions) that maintain them at these sites. The structure and function of the proposed mTORC3 complex also need further investigation.

From a clinical perspective, rapamycin and rapalogs have transitioned from laboratory-approved therapies to FDA-approved therapies for certain cancers and transplant rejection. However, their modest efficacy in solid tumors, coupled with metabolic side effects such as insulin resistance and immunosuppression, highlights the need for more selective and context-dependent modulators. The development of dual PI3K/mTOR inhibitors and third-generation compounds such as Rapalink-1 reflects this shift toward rational, structure-guided drug design aimed at overcoming resistance and achieving deeper therapeutic responses. However, challenges remain in designing therapies that preserve beneficial mTOR activity in healthy tissues.

Recent insights also underscore the systemic role of mTOR in interconnecting metabolic diseases, neurodegeneration, and cancer. The broad involvement of the pathway across tissues means that targeted inhibition can potentially improve multiple pathologies and age-associated disorders. However, this raises the risk of adverse effects, increasing the importance of tissue- and isoform-specific strategies. Moreover, the integration of mTOR-targeting agents with dietary programs, nanoformulations, and immune therapies is emerging as a promising frontier for personalized medicine. Future studies should address how systemic interventions (e.g., intermittent fasting and caloric restriction mimetics) interact with pharmacological mTOR inhibition and whether combinatorial approaches can be tailored to patient-specific metabolic and genetic backgrounds.

As our understanding of the role of mTOR in cellular metabolism, immune regulation, and disease progression deepens, it becomes increasingly clear that future therapies must strike a delicate balance, sufficiently inhibiting pathological mTOR activity while preserving its essential physiological functions. The integration of direct inhibitors with modulators of mitochondrial function and immune responses holds great promise for achieving this goal. Ultimately, rational design of targeted, context-specific, and combination-based interventions will be key to unlocking the full therapeutic potential of mTOR inhibition in both cancer and other diseases.

The next decades of mTOR research must aim for a systems-level understanding of the pathway across physiological contexts and disease states. A key priority will be identifying novel regulatory nodes, particularly in understudied cellular compartments and tissues. At the translational level, there is a need to refine therapeutic regimens and optimize dosing, timing, and combination strategies to balance efficacy with safety. Finally, as mTOR research moves increasingly toward precision and integrative medicine, the challenge will be to apply its therapeutic potential in a manner that is both specific and comprehensive, transforming decades of fundamental research into clinical outcomes.

## References

[1] Vézina, C., Kudelski, A. & Sehgal, S. N. Rapamycin (AY-22,989), a new antifungal antibiotic. I. Taxonomy of the producing streptomycete and isolation of the active principle. *J. Antibiot. (Tokyo)* **28**, 721–726 (1975). 1102508 10.7164/antibiotics.28.721

[2] Sehgal, S. N., Baker, H. & Vézina, C. Rapamycin (AY-22,989), a new antifungal antibiotic. II. Fermentation, isolation and characterization. *J. Antibiot. (Tokyo)* **28**, 727–732 (1975). 1102509 10.7164/antibiotics.28.727

[3] Duffin, J. *Stanley’s Dream: The Medical Expedition to Easter Island*. McGill-Queen’s University Press, Montreal (2019).

[4] Martel, R. R., Klicius, J. & Galet, S. Inhibition of the immune response by rapamycin, a new antifungal antibiotic. *Can. J. Physiol. Pharmacol.* **55**, 48–51 (1977). 843990 10.1139/y77-007

[5] Eng, C. P., Sehgal, S. N. & Vézina, C. Activity of rapamycin (AY-22,989) against transplanted tumors. *J. Antibiot. (Tokyo)* **37**, 1231–1237 (1984). 6501094 10.7164/antibiotics.37.1231

[6] Heitman, J., Movva, N. R. & Hall, M. N. Targets for cell cycle arrest by the immunosuppressant rapamycin in yeast. *Science* **253**, 905–909 (1991). 1715094 10.1126/science.1715094

[7] Loewith, R. et al. Two TOR complexes, only one of which is rapamycin sensitive, have distinct roles in cell growth control. *Mol. Cell* **10**, 457–468 (2002). 12408816 10.1016/s1097-2765(02)00636-6

[8] Brown, E. J. et al. A mammalian protein targeted by G1-arresting rapamycin-receptor complex. *Nature* **369**, 756–758 (1994). 8008069 10.1038/369756a0

[9] Sabatini, D. M., Erdjument-Bromage, H., Lui, M., Tempst, P. & Snyder, S. H. RAFT1: a mammalian protein that binds to FKBP12 in a rapamycin-dependent fashion and is homologous to yeast TORs. *Cell* **78**, 35–43 (1994). 7518356 10.1016/0092-8674(94)90570-3

[10] Chiu, M. I., Katz, H. & Berlin, V. RAPT1, a mammalian homolog of yeast Tor, interacts with the FKBP12/rapamycin complex. *Proc. Natl. Acad. Sci. USA* **91**, 12574–12578 (1994). 7809080 10.1073/pnas.91.26.12574 PMC45481

[11] Sabers, C. J. et al. Isolation of a protein target of the FKBP12-rapamycin complex in mammalian cells. *J. Biol. Chem.* **270**, 815–822 (1995). 7822316 10.1074/jbc.270.2.815

[12] Powers, T. The origin story of rapamycin: systemic bias in biomedical research and cold war politics. *Mol. Biol. Cell* **33**, pe7 (2022). 36228182 10.1091/mbc.E22-08-0377 PMC9634974

[13] Baker, H., Sidorowicz, A., Sehgal, S. N. & Vézina, C. Rapamycin (AY-22,989), a new antifungal antibiotic. III. In vitro and in vivo evaluation. *J. Antibiot. (Tokyo)* **31**, 539–545 (1978). 28309 10.7164/antibiotics.31.539

[14] Singh, K., Sun, S. & Vézina, C. Rapamycin (AY-22,989), a new antifungal antibiotic. IV. Mechanism of action. *J. Antibiot. (Tokyo)* **32**, 630–645 (1979). 381274 10.7164/antibiotics.32.630

[15] Swindells, D. N., White, P. S. & Findlay, J. A. The X-ray crystal structure of rapamycin, C51H79NO13. *Can. J. Chem.* **56**, 2491–2492 (1978).

[16] Dumont, F. J., Staruch, M. J., Koprak, S. L., Melino, M. R. & Sigal, N. H. Distinct mechanisms of suppression of murine T-cell activation by the related macrolides FK-506 and rapamycin. *J. Immunol.* **144**, 251–258 (1990). 1688572

[17] Koltin, Y. et al. Rapamycin sensitivity in *Saccharomyces cerevisiae* is mediated by a peptidyl-prolyl cis-trans isomerase related to human FK506-binding protein. *Mol. Cell. Biol.* **11**, 1718–1723 (1991). 1996117 10.1128/mcb.11.3.1718-1723.1991 PMC369480

[18] Siekierka, J. J. et al. The cytosolic-binding protein for the immunosuppressant FK-506 is both a ubiquitous and highly conserved peptidyl-prolyl cis-trans isomerase. *J. Biol. Chem.* **265**, 21011–21015 (1990). 1701173

[19] Livi, G. P. Halcyon days of TOR: Reflections on the multiple independent discovery of the yeast and mammalian TOR proteins. *Gene* **692**, 145–155 (2019). 30639424 10.1016/j.gene.2018.12.046

[20] Cafferkey, R. et al. Dominant missense mutations in a novel yeast protein related to mammalian phosphatidylinositol 3-kinase and VPS34 abrogate rapamycin cytotoxicity. *Mol. Cell. Biol.* **13**, 6012–6023 (1993). 8413204 10.1128/mcb.13.10.6012-6023.1993 PMC364661

[21] He, L., Cho, S. & Blenis, J. mTORC1, the maestro of cell metabolism and growth. *Genes. Dev.* **39**, 109–131 (2025). 39572234 10.1101/gad.352084.124 PMC11789495

[22] Dazert, E. & Hall, M. N. mTOR signaling in disease. *Curr. Opin. Cell. Biol.* **23**, 744–755 (2011). 21963299 10.1016/j.ceb.2011.09.003

[23] Barbet, N. C. et al. TOR controls translation initiation and early G1 progression in yeast. *Mol. Biol. Cell* **7**, 25–42 (1996). 8741837 10.1091/mbc.7.1.25 PMC278610

[24] Keith, C. T. & Schreiber, S. L. PIK-related kinases: DNA repair, recombination, and cell cycle checkpoints. *Science* **270**, 50–51 (1995). 7569949 10.1126/science.270.5233.50

[25] Helliwell, S. B. et al. TOR1 and TOR2 are structurally and functionally similar but not identical phosphatidylinositol kinase homologues in yeast. *Mol. Biol. Cell* **5**, 105–118 (1994). 8186460 10.1091/mbc.5.1.105 PMC301013

[26] Loewith, R. & Hall, M. N. Target of rapamycin (TOR) in nutrient signaling and growth control. *Genetics* **189**, 1177–1201 (2011). 22174183 10.1534/genetics.111.133363 PMC3241408

[27] Ragupathi, A., Kim, C. & Jacinto, E. The mTORC2 signaling network: Targets and cross-talks. *Biochem. J.* **481**, 45–91 (2024). 38270460 10.1042/BCJ20220325 PMC10903481

[28] Liu, G. Y. & Sabatini, D. M. mTOR at the nexus of nutrition, growth, ageing and disease. *Nat. Rev. Mol. Cell Biol.* **21**, 183–203 (2020). 31937935 10.1038/s41580-019-0199-y PMC7102936

[29] Harwood, F. C. et al. ETV7 is an essential component of a rapamycin-insensitive mTOR complex in cancer. *Sci. Adv.* **4**, eaar3938 (2018). 30258985 10.1126/sciadv.aar3938 PMC6156121

[30] Hara, K. et al. Raptor, a binding partner of target of rapamycin (TOR), mediates TOR action. *Cell* **110**, 177–189 (2002). 12150926 10.1016/s0092-8674(02)00833-4

[31] Sarbassov, D. D. et al. Rictor, a novel binding partner of mTOR, defines a rapamycin-insensitive and raptor-independent pathway that regulates the cytoskeleton. *Curr. Biol.* **14**, 1296–1302 (2004). 15268862 10.1016/j.cub.2004.06.054

[32] Yang, H. et al. 4.4 Å Resolution Cryo-EM structure of human mTOR Complex 1. *Protein Cell* **7**, 878–887 (2016). 27909983 10.1007/s13238-016-0346-6 PMC5205667

[33] Scaiola, A. et al. The 3.2-Å resolution structure of human mTORC2. *Sci. Adv.* **6**, eabc1251 (2020). 33158864 10.1126/sciadv.abc1251 PMC7673708

[34] Betz, C. & Hall, M. N. Where is mTOR and what is it doing there? *J. Cell Biol.* **203**, 563–574 (2013). 24385483 10.1083/jcb.201306041 PMC3840941

[35] Gosavi, P., Houghton, F. J., McMillan, P. J., Hanssen, E. & Gleeson, P. A. The Golgi ribbon in mammalian cells negatively regulates autophagy by modulating mTOR activity. *J. Cell Sci.* **131**, jcs211987 (2018). 29361552 10.1242/jcs.211987

[36] Goul, C., Peruzzo, R. & Zoncu, R. The molecular basis of nutrient sensing and signalling by mTORC1 in metabolism regulation and disease. *Nat. Rev. Mol. Cell Biol.* **24**, 857–875 (2023). 37612414 10.1038/s41580-023-00641-8

[37] Ebner, M., Sinkovics, B., Szczygieł, M., Ribeiro, D. W. & Yudushkin, I. Localization of mTORC2 activity inside cells. *J. Cell Biol.* **216**, 343–353 (2017). 28143890 10.1083/jcb.201610060 PMC5294791

[38] Battaglioni, S., Benjamin, D., Wälchli, M., Maier, T. & Hall, M. N. mTOR substrate phosphorylation in growth control. *Cell* **185**, 1814–1836 (2022). 35580586 10.1016/j.cell.2022.04.013

[39] Beretta, L., Gingras, A. C., Svitkin, Y. V., Hall, M. N. & Sonenberg, N. Rapamycin blocks the phosphorylation of 4E-BP1 and inhibits cap-dependent initiation of translation. *EMBO J.* **15**, 658–664 (1996). 8599949 PMC449984

[40] Hara, K. et al. Regulation of eIF-4E BP1 phosphorylation by mTOR. *J. Biol. Chem.* **272**, 26457–26463 (1997). 9334222 10.1074/jbc.272.42.26457

[41] Hara, K. et al. Amino acid sufficiency and mTOR regulate p70 S6 kinase and eIF-4E BP1 through a common effector mechanism. *J. Biol. Chem.* **273**, 14484–14494 (1998). 9603962 10.1074/jbc.273.23.14484

[42] Vega-Rubin-de-Celis, S., Peña-Llopis, S., Konda, M. & Brugarolas, J. Multistep regulation of TFEB by MTORC1. *Autophagy* **13**, 464–472 (2017). 28055300 10.1080/15548627.2016.1271514 PMC5361595

[43] Settembre, C. et al. A lysosome-to-nucleus signalling mechanism senses and regulates the lysosome via mTOR and TFEB. *EMBO J.* **31**, 1095–1108 (2012). 22343943 10.1038/emboj.2012.32 PMC3298007

[44] Martina, J. A., Chen, Y., Gucek, M. & Puertollano, R. MTORC1 functions as a transcriptional regulator of autophagy by preventing nuclear transport of TFEB. *Autophagy* **8**, 903–914 (2012). 22576015 10.4161/auto.19653 PMC3427256

[45] Martina, J. A. et al. The nutrient-responsive transcription factor TFE3 promotes autophagy, lysosomal biogenesis, and clearance of cellular debris. *Sci. Signal.* **7**, ra9 (2014). 24448649 10.1126/scisignal.2004754 PMC4696865

[46] Kim, J., Kundu, M., Viollet, B. & Guan, K.-L. AMPK and mTOR regulate autophagy through direct phosphorylation of Ulk1. *Nat. Cell Biol.* **13**, 132–141 (2011). 21258367 10.1038/ncb2152 PMC3987946

[47] Bar-Peled, L. et al. A Tumor suppressor complex with GAP activity for the Rag GTPases that signal amino acid sufficiency to mTORC1. *Science* **340**, 1100–1106 (2013). 23723238 10.1126/science.1232044 PMC3728654

[48] Gao, X. et al. Tsc tumour suppressor proteins antagonize amino-acid-TOR signalling. *Nat. Cell Biol.* **4**, 699–704 (2002). 12172555 10.1038/ncb847

[49] Inoki, K., Li, Y., Xu, T. & Guan, K.-L. Rheb GTPase is a direct target of TSC2 GAP activity and regulates mTOR signaling. *Genes Dev.* **17**, 1829–1834 (2003). 12869586 10.1101/gad.1110003 PMC196227

[50] Kim, E., Goraksha-Hicks, P., Li, L., Neufeld, T. P. & Guan, K.-L. Regulation of TORC1 by Rag GTPases in nutrient response. *Nat. Cell Biol.* **10**, 935–945 (2008). 18604198 10.1038/ncb1753 PMC2711503

[51] Sancak, Y. et al. The Rag GTPases bind raptor and mediate amino acid signaling to mTORC1. *Science* **320**, 1496–1501 (2008). 18497260 10.1126/science.1157535 PMC2475333

[52] Gollwitzer, P., Grützmacher, N., Wilhelm, S., Kümmel, D. & Demetriades, C. A Rag GTPase dimer code defines the regulation of mTORC1 by amino acids. *Nat. Cell Biol.* **24**, 1394–1406 (2022). 36097072 10.1038/s41556-022-00976-y PMC9481461

[53] Li, K. et al. Folliculin promotes substrate-selective mTORC1 activity by activating RagC to recruit TFE3. *PLoS Biol.* **20**, e3001594 (2022). 35358174 10.1371/journal.pbio.3001594 PMC9004751

[54] Napolitano, G., Di Malta, C. & Ballabio, A. Noncanonical mTORC1 signaling at the lysosome. *Trends Cell Biol.* **32**, 920–931 (2022). 35654731 10.1016/j.tcb.2022.04.012

[55] Figlia, G. et al. Brain-enriched RagB isoforms regulate the dynamics of mTORC1 activity through GATOR1 inhibition. *Nat. Cell Biol.* **24**, 1407–1421 (2022). 36097071 10.1038/s41556-022-00977-x PMC9481464

[56] Petit, C. S., Roczniak-Ferguson, A. & Ferguson, S. M. Recruitment of folliculin to lysosomes supports the amino acid-dependent activation of Rag GTPases. *J. Cell Biol.* **202**, 1107–1122 (2013). 24081491 10.1083/jcb.201307084 PMC3787382

[57] Castro, A. F., Rebhun, J. F., Clark, G. J. & Quilliam, L. A. Rheb binds tuberous sclerosis complex 2 (TSC2) and promotes S6 kinase activation in a rapamycin- and farnesylation-dependent manner. *J. Biol. Chem.* **278**, 32493–32496 (2003). 12842888 10.1074/jbc.C300226200

[58] Angarola, B. & Ferguson, S. M. Coordination of Rheb lysosomal membrane interactions with mTORC1 activation. *F1000Res* **9**, F1000 Faculty Rev-450 (2020). 32518628 10.12688/f1000research.22367.1 PMC7255682

[59] Bar-Peled, L., Schweitzer, L. D., Zoncu, R. & Sabatini, D. M. Ragulator is a GEF for the rag GTPases that signal amino acid levels to mTORC1. *Cell* **150**, 1196–1208 (2012). 22980980 10.1016/j.cell.2012.07.032 PMC3517996

[60] Feng, R. et al. The rapid proximity labeling system PhastID identifies ATP6AP1 as an unconventional GEF for Rheb. *Cell Res* **34**, 355–369 (2024). 38448650 10.1038/s41422-024-00938-z PMC11061317

[61] He, X. et al. Lysosomal EGFR acts as a Rheb-GEF independent of its kinase activity to activate mTORC1. *Cell Res* **35**, 497–509 (2025). 40259053 10.1038/s41422-025-01110-x PMC12205066

[62] Shen, K. & Sabatini, D. M. Ragulator and SLC38A9 activate the Rag GTPases through noncanonical GEF mechanisms. *Proc. Natl. Acad. Sci. USA* **115**, 9545–9550 (2018). 30181260 10.1073/pnas.1811727115 PMC6156610

[63] Wolfson, R. L. et al. Sestrin2 is a leucine sensor for the mTORC1 pathway. *Science* **351**, 43–48 (2016). 26449471 10.1126/science.aab2674 PMC4698017

[64] Chen, J. et al. SAR1B senses leucine levels to regulate mTORC1 signalling. *Nature* **596**, 281–284 (2021). 34290409 10.1038/s41586-021-03768-w

[65] Chantranupong, L. et al. The CASTOR proteins are Arginine sensors for the mTORC1 pathway. *Cell* **165**, 153–164 (2016). 26972053 10.1016/j.cell.2016.02.035 PMC4808398

[66] Kim, S.-H. et al. Mitochondrial Threonyl-tRNA Synthetase TARS2 is required for Threonine-sensitive mTORC1 activation. *Mol. Cell* **81**, 398–407.e4 (2021). 33340489 10.1016/j.molcel.2020.11.036

[67] Rebsamen, M. et al. SLC38A9 is a component of the lysosomal amino acid sensing machinery that controls mTORC1. *Nature* **519**, 477–481 (2015). 25561175 10.1038/nature14107 PMC4376665

[68] Wang, S. et al. Metabolism. Lysosomal amino acid transporter SLC38A9 signals arginine sufficiency to mTORC1. *Science* **347**, 188–194 (2015). 25567906 10.1126/science.1257132 PMC4295826

[69] Jung, J., Genau, H. M. & Behrends, C. Amino acid-dependent mTORC1 regulation by the lysosomal membrane protein SLC38A9. *Mol. Cell. Biol.* **35**, 2479–2494 (2015). 25963655 10.1128/MCB.00125-15 PMC4475919

[70] Gu, X. et al. SAMTOR is an S-adenosylmethionine sensor for the mTORC1 pathway. *Science* **358**, 813–818 (2017). 29123071 10.1126/science.aao3265 PMC5747364

[71] Tang, X. et al. Molecular mechanism of S-adenosylmethionine sensing by SAMTOR in mTORC1 signaling. *Sci. Adv.* **8**, eabn3868 (2022). 35776786 10.1126/sciadv.abn3868 PMC10883374

[72] Jiang, C. et al. PRMT1 orchestrates with SAMTOR to govern mTORC1 methionine sensing via Arg-methylation of NPRL2. *Cell Metab.* **35**, 2183–2199.e7 (2023). 38006878 10.1016/j.cmet.2023.11.001 PMC11192564

[73] Shen, K. et al. Architecture of the human GATOR1 and GATOR1-Rag GTPases complexes. *Nature* **556**, 64–69 (2018). 29590090 10.1038/nature26158 PMC5975964

[74] Shen, K., Valenstein, M. L., Gu, X. & Sabatini, D. M. Arg-78 of Nprl2 catalyzes GATOR1-stimulated GTP hydrolysis by the Rag GTPases. *J. Biol. Chem.* **294**, 2970–2975 (2019). 30651352 10.1074/jbc.AC119.007382 PMC6393612

[75] Dokudovskaya, S. et al. A conserved coatomer-related complex containing Sec13 and Seh1 dynamically associates with the vacuole in *Saccharomyces cerevisiae*. *Mol. Cell. Proteom.* **10**, M110.006478 (2011). 10.1074/mcp.M110.006478 PMC3108837 21454883

[76] Loissell-Baltazar, Y. A. & Dokudovskaya, S. SEA and GATOR 10 years later. *Cells* **10**, 2689 (2021). 34685669 10.3390/cells10102689 PMC8534245

[77] Jiang, C. et al. Ring domains are essential for GATOR2-dependent mTORC1 activation. *Mol. Cell* **83**, 74–89.e9 (2023). 36528027 10.1016/j.molcel.2022.11.021 PMC11027793

[78] Dokudovskaya, S. & Rout, M. P. SEA you later alli-GATOR–a dynamic regulator of the TORC1 stress response pathway. *J. Cell Sci.* **128**, 2219–2228 (2015). 25934700 10.1242/jcs.168922 PMC4487016

[79] Algret, R. et al. Molecular architecture and function of the SEA complex, a modulator of the TORC1 pathway. *Mol. Cell. Proteom.* **13**, 2855–2870 (2014). 10.1074/mcp.M114.039388 PMC4223477 25073740

[80] Tafur, L. et al. Cryo-EM structure of the SEA complex. *Nature* **611**, 399–404 (2022). 36289347 10.1038/s41586-022-05370-0 PMC9646525

[81] Wolfson, R. L. et al. KICSTOR recruits GATOR1 to the lysosome and is necessary for nutrients to regulate mTORC1. *Nature* **543**, 438–442 (2017). 28199306 10.1038/nature21423 PMC5360989

[82] Zhao, T. et al. VWCE modulates amino acid-dependent mTOR signaling and coordinates with KICSTOR to recruit GATOR1 to the lysosomes. *Nat. Commun.* **14**, 8464 (2023). 38123554 10.1038/s41467-023-44241-8 PMC10733324

[83] Yan, G. et al. Genome-wide CRISPR screens identify ILF3 as a mediator of mTORC1-dependent amino acid sensing. *Nat. Cell Biol.* **25**, 754–764 (2023). 37037994 10.1038/s41556-023-01123-x

[84] Parmigiani, A. et al. Sestrins inhibit mTORC1 kinase activation through the GATOR complex. *Cell Rep.* **9**, 1281–1291 (2014). 25457612 10.1016/j.celrep.2014.10.019 PMC4303546

[85] Saxton, R. A. et al. Structural basis for leucine sensing by the Sestrin2-mTORC1 pathway. *Science* **351**, 53–58 (2016). 26586190 10.1126/science.aad2087 PMC4698039

[86] Han, J. M. et al. Leucyl-tRNA synthetase is an intracellular leucine sensor for the mTORC1-signaling pathway. *Cell* **149**, 410–424 (2012). 22424946 10.1016/j.cell.2012.02.044

[87] Livneh, I. et al. Regulation of nucleo-cytosolic 26S proteasome translocation by aromatic amino acids via mTOR is essential for cell survival under stress. *Mol. Cell* **83**, 3333–3346.e5 (2023). 37738964 10.1016/j.molcel.2023.08.016

[88] Valenstein, M. L. et al. Rag-Ragulator is the central organizer of the physical architecture of the mTORC1 nutrient-sensing pathway. *Proc. Natl. Acad. Sci. USA* **121**, e2322755121 (2024). 39163330 10.1073/pnas.2322755121 PMC11363303

[89] Jewell, J. L. et al. Metabolism. Differential regulation of mTORC1 by leucine and glutamine. *Science* **347**, 194–198 (2015). 25567907 10.1126/science.1259472 PMC4384888

[90] Meng, D. et al. Glutamine and asparagine activate mTORC1 independently of Rag GTPases. *J. Biol. Chem.* **295**, 2890–2899 (2020). 32019866 10.1074/jbc.AC119.011578 PMC7062167

[91] Thomas, J. D. et al. Rab1A is an mTORC1 activator and a colorectal oncogene. *Cancer Cell* **26**, 754–769 (2014). 25446900 10.1016/j.ccell.2014.09.008 PMC4288827

[92] Ukai, H. et al. Gtr/Ego-independent TORC1 activation is achieved through a glutamine-sensitive interaction with Pib2 on the vacuolar membrane. *PLoS Genet* **14**, e1007334 (2018). 29698392 10.1371/journal.pgen.1007334 PMC5919408

[93] Hesketh, G. G. et al. The GATOR-Rag GTPase pathway inhibits mTORC1 activation by lysosome-derived amino acids. *Science* **370**, 351–356 (2020). 33060361 10.1126/science.aaz0863

[94] Mutvei, A. P. et al. Rap1-GTPases control mTORC1 activity by coordinating lysosome organization with amino acid availability. *Nat. Commun.* **11**, 1416 (2020). 32184389 10.1038/s41467-020-15156-5 PMC7078236

[95] Angarola, B. & Ferguson, S. M. Weak membrane interactions allow Rheb to activate mTORC1 signaling without major lysosome enrichment. *Mol. Biol. Cell* **30**, 2750–2760 (2019). 31532697 10.1091/mbc.E19-03-0146 PMC6789162

[96] Zhong, Y., Zhou, X., Guan, K.-L. & Zhang, J. Rheb regulates nuclear mTORC1 activity independent of farnesylation. *Cell Chem. Biol.* **29**, 1037–1045.e4 (2022). 35294906 10.1016/j.chembiol.2022.02.006 PMC9667991

[97] Fernandes, S. A. et al. Spatial and functional separation of mTORC1 signalling in response to different amino acid sources. *Nat. Cell Biol.* **26**, 1918–1933 (2024). 39385049 10.1038/s41556-024-01523-7 PMC11567901

[98] Wolfson, R. L. & Sabatini, D. M. The dawn of the age of amino acid sensors for the mTORC1 pathway. *Cell Metab.* **26**, 301–309 (2017). 28768171 10.1016/j.cmet.2017.07.001 PMC5560103

[99] Liu, G. Y., Jouandin, P., Bahng, R. E., Perrimon, N. & Sabatini, D. M. An evolutionary mechanism to assimilate new nutrient sensors into the mTORC1 pathway. *Nat. Commun.* **15**, 2517 (2024). 38514639 10.1038/s41467-024-46680-3 PMC10957897

[100] Bettedi, L., Zhang, Y., Yang, S. & Lilly, M. A. Unveiling GATOR2 function: novel insights from Drosophila research. *Cells* **13**, 1795 (2024). 39513902 10.3390/cells13211795 PMC11545208

[101] Nicastro, R., Sardu, A., Panchaud, N. & De Virgilio, C. The architecture of the Rag GTPase signaling network. *Biomolecules* **7**, 48 (2017). 28788436 10.3390/biom7030048 PMC5618229

[102] Yang, H. et al. Structural insights into TSC complex assembly and GAP activity on Rheb. *Nat. Commun.* **12**, 339 (2021). 33436626 10.1038/s41467-020-20522-4 PMC7804450

[103] Garami, A. et al. Insulin activation of Rheb, a mediator of mTOR/S6K/4E-BP signaling, is inhibited by TSC1 and 2. *Mol. Cell* **11**, 1457–1466 (2003). 12820960 10.1016/s1097-2765(03)00220-x

[104] Zhang, Y. et al. Rheb is a direct target of the tuberous sclerosis tumour suppressor proteins. *Nat. Cell Biol.* **5**, 578–581 (2003). 12771962 10.1038/ncb999

[105] Potter, C. J., Pedraza, L. G. & Xu, T. Akt regulates growth by directly phosphorylating Tsc2. *Nat. Cell Biol.* **4**, 658–665 (2002). 12172554 10.1038/ncb840

[106] Menon, S. et al. Spatial control of the TSC complex integrates insulin and nutrient regulation of mTORC1 at the lysosome. *Cell* **156**, 771–785 (2014). 24529379 10.1016/j.cell.2013.11.049 PMC4030681

[107] Shah, O. J., Wang, Z. & Hunter, T. Inappropriate activation of the TSC/Rheb/mTOR/S6K cassette induces IRS1/2 depletion, insulin resistance, and cell survival deficiencies. *Curr. Biol.* **14**, 1650–1656 (2004). 15380067 10.1016/j.cub.2004.08.026

[108] Harrington, L. S. et al. The TSC1-2 tumor suppressor controls insulin-PI3K signaling via regulation of IRS proteins. *J. Cell Biol.* **166**, 213–223 (2004). 15249583 10.1083/jcb.200403069 PMC2172316

[109] Ma, L., Chen, Z., Erdjument-Bromage, H., Tempst, P. & Pandolfi, P. P. Phosphorylation and functional inactivation of TSC2 by Erk implications for tuberous sclerosis and cancer pathogenesis. *Cell* **121**, 179–193 (2005). 15851026 10.1016/j.cell.2005.02.031

[110] Lee, D.-F. et al. IKK beta suppression of TSC1 links inflammation and tumor angiogenesis via the mTOR pathway. *Cell* **130**, 440–455 (2007). 17693255 10.1016/j.cell.2007.05.058

[111] Inoki, K. et al. TSC2 integrates Wnt and energy signals via a coordinated phosphorylation by AMPK and GSK3 to regulate cell growth. *Cell* **126**, 955–968 (2006). 16959574 10.1016/j.cell.2006.06.055

[112] Vander Haar, E., Lee, S.-I., Bandhakavi, S., Griffin, T. J. & Kim, D.-H. Insulin signalling to mTOR mediated by the Akt/PKB substrate PRAS40. *Nat. Cell Biol.* **9**, 316–323 (2007). 17277771 10.1038/ncb1547

[113] Sancak, Y. et al. PRAS40 is an insulin-regulated inhibitor of the mTORC1 protein kinase. *Mol. Cell* **25**, 903–915 (2007). 17386266 10.1016/j.molcel.2007.03.003

[114] Muller, M. et al. GATOR1 mutations impair PI3 Kinase-dependent growth factor signaling regulation of mTORC1. *Int. J. Mol. Sci.* **25**, 2068 (2024). 38396745 10.3390/ijms25042068 PMC10889792

[115] Gwinn, D. M. et al. AMPK phosphorylation of raptor mediates a metabolic checkpoint. *Mol. Cell* **30**, 214–226 (2008). 18439900 10.1016/j.molcel.2008.03.003 PMC2674027

[116] Inoki, K., Zhu, T. & Guan, K.-L. TSC2 mediates cellular energy response to control cell growth and survival. *Cell* **115**, 577–590 (2003). 14651849 10.1016/s0092-8674(03)00929-2

[117] Dai, X. et al. AMPK-dependent phosphorylation of the GATOR2 component WDR24 suppresses glucose-mediated mTORC1 activation. *Nat. Metab.* **5**, 265–276 (2023). 36732624 10.1038/s42255-022-00732-4 PMC11070849

[118] Yoon, I. et al. Glucose-dependent control of leucine metabolism by leucyl-tRNA synthetase 1. *Science* **367**, 205–210 (2020). 31780625 10.1126/science.aau2753

[119] Herzig, S. & Shaw, R. J. AMPK: guardian of metabolism and mitochondrial homeostasis. *Nat. Rev. Mol. Cell Biol.* **19**, 121–135 (2018). 28974774 10.1038/nrm.2017.95 PMC5780224

[120] Orozco, J. M. et al. Dihydroxyacetone phosphate signals glucose availability to mTORC1. *Nat. Metab.* **2**, 893–901 (2020). 32719541 10.1038/s42255-020-0250-5 PMC7995735

[121] Li, M. et al. Aldolase is a sensor for both low and high glucose, linking to AMPK and mTORC1. *Cell Res* **31**, 478–481 (2021). 33349646 10.1038/s41422-020-00456-8 PMC8115481

[122] Chen, X. et al. Intracellular galectin-3 is a lipopolysaccharide sensor that promotes glycolysis through mTORC1 activation. *Nat. Commun.* **13**, 7578 (2022). 36481721 10.1038/s41467-022-35334-x PMC9732310

[123] Shin, H. R. et al. Lysosomal GPCR-like protein LYCHOS signals cholesterol sufficiency to mTORC1. *Science* **377**, 1290–1298 (2022). 36007018 10.1126/science.abg6621 PMC10023259

[124] Castellano, B. M. et al. Lysosomal cholesterol activates mTORC1 via an SLC38A9-Niemann-Pick C1 signaling complex. *Science* **355**, 1306–1311 (2017). 28336668 10.1126/science.aag1417 PMC5823611

[125] Koundouros, N. et al. Direct sensing of dietary ω-6 linoleic acid through FABP5-mTORC1 signaling. *Science* **387**, eadm9805 (2025). 40080571 10.1126/science.adm9805

[126] Menon, D. et al. Lipid sensing by mTOR complexes via de novo synthesis of phosphatidic acid. *J. Biol. Chem.* **292**, 6303–6311 (2017). 28223357 10.1074/jbc.M116.772988 PMC5391759

[127] Alexander, A. et al. ATM signals to TSC2 in the cytoplasm to regulate mTORC1 in response to ROS. *Proc. Natl. Acad. Sci. USA* **107**, 4153–4158 (2010). 20160076 10.1073/pnas.0913860107 PMC2840158

[128] Ma, Y., Vassetzky, Y. & Dokudovskaya, S. mTORC1 pathway in DNA damage response. *Biochim. Biophys. Acta Mol. Cell Res.* **1865**, 1293–1311 (2018). 29936127 10.1016/j.bbamcr.2018.06.011

[129] Sancar, A., Lindsey-Boltz, L. A., Unsal-Kaçmaz, K. & Linn, S. Molecular mechanisms of mammalian DNA repair and the DNA damage checkpoints. *Annu. Rev. Biochem.* **73**, 39–85 (2004). 15189136 10.1146/annurev.biochem.73.011303.073723

[130] Feng, Z. et al. The regulation of AMPK beta1, TSC2, and PTEN expression by p53: stress, cell and tissue specificity, and the role of these gene products in modulating the IGF-1-AKT-mTOR pathways. *Cancer Res* **67**, 3043–3053 (2007). 17409411 10.1158/0008-5472.CAN-06-4149

[131] Lai, K. P. et al. S6K1 is a multifaceted regulator of Mdm2 that connects nutrient status and DNA damage response. *EMBO J.* **29**, 2994–3006 (2010). 20657550 10.1038/emboj.2010.166 PMC2944047

[132] Budanov, A. V. & Karin, M. p53 target genes sestrin1 and sestrin2 connect genotoxic stress and mTOR signaling. *Cell* **134**, 451–460 (2008). 18692468 10.1016/j.cell.2008.06.028 PMC2758522

[133] Muñoz-Gámez, J. A. et al. PARP-1 is involved in autophagy induced by DNA damage. *Autophagy* **5**, 61–74 (2009). 19001878 10.4161/auto.5.1.7272

[134] Rodríguez-Vargas, J. M. et al. ROS-induced DNA damage and PARP-1 are required for optimal induction of starvation-induced autophagy. *Cell Res* **22**, 1181–1198 (2012). 22525338 10.1038/cr.2012.70 PMC3391023

[135] Li, Y. et al. Protein phosphatase 2A and DNA-dependent protein kinase are involved in mediating rapamycin-induced Akt phosphorylation. *J. Biol. Chem.* **288**, 13215–13224 (2013). 23536185 10.1074/jbc.M113.463679 PMC3650361

[136] Thedieck, K. et al. PRAS40 and PRR5-like protein are new mTOR interactors that regulate apoptosis. *PLoS One* **2**, e1217 (2007). 18030348 10.1371/journal.pone.0001217 PMC2075366

[137] Inoki, K., Li, Y., Zhu, T., Wu, J. & Guan, K.-L. TSC2 is phosphorylated and inhibited by Akt and suppresses mTOR signalling. *Nat. Cell Biol.* **4**, 648–657 (2002). 12172553 10.1038/ncb839

[138] Yuan, H.-X. & Guan, K.-L. The SIN1-PH Domain Connects mTORC2 to PI3K. *Cancer Discov.* **5**, 1127–1129 (2015). 26526694 10.1158/2159-8290.CD-15-1125 PMC4638136

[139] Liu, P. et al. PtdIns(3,4,5)P3-dependent activation of the mTORC2 kinase complex. *Cancer Discov.* **5**, 1194–1209 (2015). 26293922 10.1158/2159-8290.CD-15-0460 PMC4631654

[140] Senoo, H. et al. KARATE: PKA-induced KRAS4B-RHOA-mTORC2 supercomplex phosphorylates AKT in insulin signaling and glucose homeostasis. *Mol. Cell* **81**, 4622–4634.e8 (2021). 34551282 10.1016/j.molcel.2021.09.001

[141] Oh, W. J. et al. mTORC2 can associate with ribosomes to promote cotranslational phosphorylation and stability of nascent Akt polypeptide. *EMBO J.* **29**, 3939–3951 (2010). 21045808 10.1038/emboj.2010.271 PMC3020639

[142] Zinzalla, V., Stracka, D., Oppliger, W. & Hall, M. N. Activation of mTORC2 by association with the ribosome. *Cell* **144**, 757–768 (2011). 21376236 10.1016/j.cell.2011.02.014

[143] Sato, M. et al. Improving type 2 diabetes through a distinct adrenergic signaling pathway involving mTORC2 that mediates glucose uptake in skeletal muscle. *Diabetes* **63**, 4115–4129 (2014). 25008179 10.2337/db13-1860

[144] Sato, M. et al. α1A-Adrenoceptors activate mTOR signalling and glucose uptake in cardiomyocytes. *Biochem. Pharmacol.* **148**, 27–40 (2018). 29175420 10.1016/j.bcp.2017.11.016

[145] Kim, T. et al. Hepatic glucagon receptor signaling enhances insulin-stimulated glucose disposal in rodents. *Diabetes* **67**, 2157–2166 (2018). 30150304 10.2337/db18-0068 PMC6198333

[146] Tato, I., Bartrons, R., Ventura, F. & Rosa, J. L. Amino acids activate mammalian target of rapamycin complex 2 (mTORC2) via PI3K/Akt signaling. *J. Biol. Chem.* **286**, 6128–6142 (2011). 21131356 10.1074/jbc.M110.166991 PMC3057817

[147] Kazyken, D., Lentz, S. I. & Fingar, D. C. Alkaline intracellular pH (pHi) activates AMPK-mTORC2 signaling to promote cell survival during growth factor limitation. *J. Biol. Chem.* **297**, 101100 (2021). 34418433 10.1016/j.jbc.2021.101100 PMC8479482

[148] Merhi, A., Delrée, P. & Marini, A. M. The metabolic waste ammonium regulates mTORC2 and mTORC1 signaling. *Sci. Rep.* **7**, 44602 (2017). 28303961 10.1038/srep44602 PMC5355986

[149] Kazyken, D. et al. AMPK directly activates mTORC2 to promote cell survival during acute energetic stress. *Sci. Signal.* **12**, eaav3249 (2019). 31186373 10.1126/scisignal.aav3249 PMC6935248

[150] Wang, G. et al. Cystine induced-mTORC2 activation through promoting Sin1 phosphorylation to suppress cancer cell ferroptosis. *Mol. Nutr. Food Res.* **66**, e2200186 (2022). 36189894 10.1002/mnfr.202200186

[151] Moloughney, J. G. et al. mTORC2 responds to glutamine catabolite levels to modulate the hexosamine biosynthesis enzyme GFAT1. *Mol. Cell* **63**, 811–826 (2016). 27570073 10.1016/j.molcel.2016.07.015 PMC5006067

[152] Masui, K. et al. Glucose-dependent acetylation of Rictor promotes targeted cancer therapy resistance. *Proc. Natl. Acad. Sci. USA* **112**, 9406–9411 (2015). 26170313 10.1073/pnas.1511759112 PMC4522814

[153] Lee, G. et al. Posttranscriptional regulation of de novo lipogenesis by mTORC1-S6K1-SRPK2 signaling. *Cell* **171**, 1545–1558.e18 (2017). 29153836 10.1016/j.cell.2017.10.037 PMC5920692

[154] Cho, S. et al. mTORC1 promotes cell growth via m6A-dependent mRNA degradation. *Mol. Cell* **81**, 2064–2075.e8 (2021). 33756105 10.1016/j.molcel.2021.03.010 PMC8356906

[155] Cho, S. et al. FAM120A couples SREBP-dependent transcription and splicing of lipogenesis enzymes downstream of mTORC1. *Mol. Cell* **83**, 3010–3026.e8 (2023). 37595559 10.1016/j.molcel.2023.07.017 PMC10494788

[156] Tang, H.-W. et al. mTORC1-chaperonin CCT signaling regulates m6A RNA methylation to suppress autophagy. *Proc. Natl. Acad. Sci. USA* **118**, e2021945118 (2021). 33649236 10.1073/pnas.2021945118 PMC7958400

[157] Villa, E. et al. mTORC1 stimulates cell growth through SAM synthesis and m6A mRNA-dependent control of protein synthesis. *Mol. Cell* **81**, 2076–2093.e9 (2021). 33756106 10.1016/j.molcel.2021.03.009 PMC8141029

[158] Ma, X. M., Yoon, S.-O., Richardson, C. J., Jülich, K. & Blenis, J. SKAR links pre-mRNA splicing to mTOR/S6K1-mediated enhanced translation efficiency of spliced mRNAs. *Cell* **133**, 303–313 (2008). 18423201 10.1016/j.cell.2008.02.031

[159] Huang, W. et al. Decreased spliceosome fidelity and egl-8 intron retention inhibit mTORC1 signaling to promote longevity. *Nat. Aging* **2**, 796–808 (2022). 37118503 10.1038/s43587-022-00275-z PMC10154236

[160] Yang, C. et al. The role of m6A modification in physiology and disease. *Cell Death Dis.* **11**, 960 (2020). 33162550 10.1038/s41419-020-03143-z PMC7649148

[161] Wolfe, A. L. et al. RNA G-quadruplexes cause eIF4A-dependent oncogene translation in cancer. *Nature* **513**, 65–70 (2014). 25079319 10.1038/nature13485 PMC4492470

[162] Zhu, J., Blenis, J. & Yuan, J. Activation of PI3K/Akt and MAPK pathways regulates Myc-mediated transcription by phosphorylating and promoting the degradation of Mad1. *Proc. Natl. Acad. Sci. USA* **105**, 6584–6589 (2008). 18451027 10.1073/pnas.0802785105 PMC2373325

[163] Fumagalli, S. & Pende, M. S6 kinase 1 at the central node of cell size and ageing. *Front. Cell Dev. Biol.* **10**, 949196 (2022). 36036012 10.3389/fcell.2022.949196 PMC9417411

[164] Gingras, A. C. et al. Regulation of 4E-BP1 phosphorylation: a novel two-step mechanism. *Genes Dev.* **13**, 1422–1437 (1999). 10364159 10.1101/gad.13.11.1422 PMC316780

[165] Thoreen, C. C. et al. A unifying model for mTORC1-mediated regulation of mRNA translation. *Nature* **485**, 109–113 (2012). 22552098 10.1038/nature11083 PMC3347774

[166] Philippe, L., van den Elzen, A. M. G., Watson, M. J. & Thoreen, C. C. Global analysis of LARP1 translation targets reveals tunable and dynamic features of 5’ TOP motifs. *Proc. Natl. Acad. Sci. USA* **117**, 5319–5328 (2020). 32094190 10.1073/pnas.1912864117 PMC7071917

[167] Hong, S. et al. LARP1 functions as a molecular switch for mTORC1-mediated translation of an essential class of mRNAs. *eLife* **6**, e25237 (2017). 28650797 10.7554/eLife.25237 PMC5484620

[168] Jia, J.-J. et al. mTORC1 promotes TOP mRNA translation through site-specific phosphorylation of LARP1. *Nucleic Acids Res* **49**, 3461–3489 (2021). 33398329 10.1093/nar/gkaa1239 PMC8034618

[169] Chang, J.-W. et al. mRNA 3’-UTR shortening is a molecular signature of mTORC1 activation. *Nat. Commun.* **6**, 7218 (2015). 26074333 10.1038/ncomms8218

[170] Chang, J.-W. et al. An integrative model for alternative polyadenylation, IntMAP, delineates mTOR-modulated endoplasmic reticulum stress response. *Nucleic Acids Res* **46**, 5996–6008 (2018). 29733382 10.1093/nar/gky340 PMC6158760

[171] Mayer, C., Zhao, J., Yuan, X. & Grummt, I. mTOR-dependent activation of the transcription factor TIF-IA links rRNA synthesis to nutrient availability. *Genes Dev.* **18**, 423–434 (2004). 15004009 10.1101/gad.285504 PMC359396

[172] Iadevaia, V., Caldarola, S., Tino, E., Amaldi, F. & Loreni, F. All translation elongation factors and the e, f, and h subunits of translation initiation factor 3 are encoded by 5’-terminal oligopyrimidine (TOP) mRNAs. *RNA* **14**, 1730–1736 (2008). 18658124 10.1261/rna.1037108 PMC2525946

[173] Kantidakis, T., Ramsbottom, B. A., Birch, J. L., Dowding, S. N. & White, R. J. mTOR associates with TFIIIC, is found at tRNA and 5S rRNA genes, and targets their repressor Maf1. *Proc. Natl. Acad. Sci. USA* **107**, 11823–11828 (2010). 20543138 10.1073/pnas.1005188107 PMC2900655

[174] Michels, A. A. et al. mTORC1 directly phosphorylates and regulates human MAF1. *Mol. Cell. Biol.* **30**, 3749–3757 (2010). 20516213 10.1128/MCB.00319-10 PMC2916396

[175] Shor, B. et al. Requirement of the mTOR kinase for the regulation of Maf1 phosphorylation and control of RNA polymerase III-dependent transcription in cancer cells. *J. Biol. Chem.* **285**, 15380–15392 (2010). 20233713 10.1074/jbc.M109.071639 PMC2865278

[176] Delarue, M. et al. mTORC1 controls phase separation and the biophysical properties of the cytoplasm by tuning crowding. *Cell* **174**, 338–349.e20 (2018). 29937223 10.1016/j.cell.2018.05.042 PMC10080728

[177] Ben-Sahra, I., Hoxhaj, G., Ricoult, S. J. H., Asara, J. M. & Manning, B. D. mTORC1 induces purine synthesis through control of the mitochondrial tetrahydrofolate cycle. *Science* **351**, 728–733 (2016). 26912861 10.1126/science.aad0489 PMC4786372

[178] Ben-Sahra, I., Howell, J. J., Asara, J. M. & Manning, B. D. Stimulation of de novo pyrimidine synthesis by growth signaling through mTOR and S6K1. *Science* **339**, 1323–1328 (2013). 23429703 10.1126/science.1228792 PMC3753690

[179] Robitaille, A. M. et al. Quantitative phosphoproteomics reveal mTORC1 activates de novo pyrimidine synthesis. *Science* **339**, 1320–1323 (2013). 23429704 10.1126/science.1228771

[180] Valvezan, A. J. et al. mTORC1 couples nucleotide synthesis to nucleotide demand resulting in a targetable metabolic vulnerability. *Cancer Cell* **32**, 624–638.e5 (2017). 29056426 10.1016/j.ccell.2017.09.013 PMC5687294

[181] Düvel, K. et al. Activation of a metabolic gene regulatory network downstream of mTOR complex 1. *Mol. Cell* **39**, 171–183 (2010). 20670887 10.1016/j.molcel.2010.06.022 PMC2946786

[182] Peterson, T. R. et al. mTOR complex 1 regulates lipin 1 localization to control the SREBP pathway. *Cell* **146**, 408–420 (2011). 21816276 10.1016/j.cell.2011.06.034 PMC3336367

[183] Hu, Y. & Reggiori, F. Molecular regulation of autophagosome formation. *Biochem. Soc. Trans.* **50**, 55–69 (2022). 35076688 10.1042/BST20210819 PMC9022990

[184] Licheva, M., Raman, B., Kraft, C. & Reggiori, F. Phosphoregulation of the autophagy machinery by kinases and phosphatases. *Autophagy* **18**, 104–123 (2022). 33970777 10.1080/15548627.2021.1909407 PMC8865292

[185] Duran, A. et al. p62 is a key regulator of nutrient sensing in the mTORC1 pathway. *Mol. Cell* **44**, 134–146 (2011). 21981924 10.1016/j.molcel.2011.06.038 PMC3190169

[186] Abudu, Y. P., Kournoutis, A., Brenne, H. B., Lamark, T. & Johansen, T. MORG1 limits mTORC1 signaling by inhibiting Rag GTPases. *Mol. Cell* **84**, 552–569.e11 (2024). 38103557 10.1016/j.molcel.2023.11.023

[187] Egan, D. F. et al. Phosphorylation of ULK1 (hATG1) by AMP-activated protein kinase connects energy sensing to mitophagy. *Science* **331**, 456–461 (2011). 21205641 10.1126/science.1196371 PMC3030664

[188] Pan, Z., Zhang, H. & Dokudovskaya, S. The role of mTORC1 pathway and autophagy in resistance to platinum-based chemotherapeutics. *Int. J. Mol. Sci.* **24**, 10651 (2023). 37445831 10.3390/ijms241310651 PMC10341996

[189] Zhang, Y. et al. Coordinated regulation of protein synthesis and degradation by mTORC1. *Nature* **513**, 440–443 (2014). 25043031 10.1038/nature13492 PMC4402229

[190] Shimizu, K. et al. The SCFβ-TRCP E3 ubiquitin ligase complex targets Lipin1 for ubiquitination and degradation to promote hepatic lipogenesis. *Sci. Signal.* **10**, eaah4117 (2017). 28049764 10.1126/scisignal.aah4117 PMC5215841

[191] Zhao, J., Zhai, B., Gygi, S. P. & Goldberg, A. L. mTOR inhibition activates overall protein degradation by the ubiquitin proteasome system as well as by autophagy. *Proc. Natl. Acad. Sci. USA* **112**, 15790–15797 (2015). 26669439 10.1073/pnas.1521919112 PMC4703015

[192] Livneh, I. et al. Inhibition of nucleo-cytoplasmic proteasome translocation by the aromatic amino acids or silencing Sestrin3-their sensing mediator-is tumor suppressive. *Cell Death Differ.* **31**, 1242–1254 (2024). 39266717 10.1038/s41418-024-01370-x PMC11445514

[193] Manning, B. D. & Toker, A. AKT/PKB signaling: Navigating the network. *Cell* **169**, 381–405 (2017). 28431241 10.1016/j.cell.2017.04.001 PMC5546324

[194] Sarbassov, D. D., Guertin, D. A., Ali, S. M. & Sabatini, D. M. Phosphorylation and regulation of Akt/PKB by the Rictor-mTOR complex. *Science* **307**, 1098–1101 (2005). 15718470 10.1126/science.1106148

[195] Hresko, R. C. & Mueckler, M. mTOR.RICTOR is the Ser473 kinase for Akt/protein kinase B in 3T3-L1 adipocytes. *J. Biol. Chem.* **280**, 40406–40416 (2005). 16221682 10.1074/jbc.M508361200

[196] Facchinetti, V. et al. The mammalian target of rapamycin complex 2 controls folding and stability of Akt and protein kinase C. *EMBO J.* **27**, 1932–1943 (2008). 18566586 10.1038/emboj.2008.120 PMC2486276

[197] Chen, C.-H. et al. Autoregulation of the mechanistic target of rapamycin (mTOR) complex 2 integrity is controlled by an ATP-dependent mechanism. *J. Biol. Chem.* **288**, 27019–27030 (2013). 23928304 10.1074/jbc.M113.498055 PMC3779703

[198] Baffi, T. R. & Newton, A. C. Protein kinase C: release from quarantine by mTORC2. *Trends Biochem. Sci.* **47**, 518–530 (2022). 35361526 10.1016/j.tibs.2022.03.003 PMC10936736

[199] Gleason, C. E. et al. Phosphorylation at distinct subcellular locations underlies specificity in mTORC2-mediated activation of SGK1 and Akt. *J. Cell Sci.* **132**, jcs224931 (2019). 30837283 10.1242/jcs.224931 PMC6467483

[200] Szwed, A., Kim, E. & Jacinto, E. Regulation and metabolic functions of mTORC1 and mTORC2. *Physiol. Rev.* **101**, 1371–1426 (2021). 33599151 10.1152/physrev.00026.2020 PMC8424549

[201] de la Cruz López, K. G., Toledo Guzmán, M. E., Sánchez, E. O. & García Carrancá A. mTORC1 as a regulator of mitochondrial functions and a therapeutic target in cancer. *Front. Oncol.* **9**, 1373 (2019). 31921637 10.3389/fonc.2019.01373 PMC6923780

[202] Ni, H.-M., Williams, J. A. & Ding, W.-X. Mitochondrial dynamics and mitochondrial quality control. *Redox Biol.* **4**, 6–13 (2015). 25479550 10.1016/j.redox.2014.11.006 PMC4309858

[203] Popov, L.-D. Mitochondrial biogenesis: An update. *J. Cell. Mol. Med.* **24**, 4892–4899 (2020). 32279443 10.1111/jcmm.15194 PMC7205802

[204] Ploumi, C., Daskalaki, I. & Tavernarakis, N. Mitochondrial biogenesis and clearance: a balancing act. *FEBS J.* **284**, 183–195 (2017). 27462821 10.1111/febs.13820

[205] Priesnitz, C. & Becker, T. Pathways to balance mitochondrial translation and protein import. *Genes Dev.* **32**, 1285–1296 (2018). 30275044 10.1101/gad.316547.118 PMC6169841

[206] Quirós, P. M., Mottis, A. & Auwerx, J. Mitonuclear communication in homeostasis and stress. *Nat. Rev. Mol. Cell Biol.* **17**, 213–226 (2016). 26956194 10.1038/nrm.2016.23

[207] Jazwinski, S. M. The retrograde response: a conserved compensatory reaction to damage from within and from without. *Prog. Mol. Biol. Transl. Sci.* **127**, 133–154 (2014). 25149216 10.1016/B978-0-12-394625-6.00005-2 PMC4271643

[208] Cunningham, J. T. et al. mTOR controls mitochondrial oxidative function through a YY1-PGC-1alpha transcriptional complex. *Nature* **450**, 736–740 (2007). 18046414 10.1038/nature06322

[209] Jäger, S., Handschin, C., St-Pierre, J. & Spiegelman, B. M. AMP-activated protein kinase (AMPK) action in skeletal muscle via direct phosphorylation of PGC-1alpha. *Proc. Natl. Acad. Sci. USA* **104**, 12017–12022 (2007). 17609368 10.1073/pnas.0705070104 PMC1924552

[210] Marin, T. L. et al. AMPK promotes mitochondrial biogenesis and function by phosphorylating the epigenetic factors DNMT1, RBBP7, and HAT1. *Sci. Signal.* **10**, eaaf7478 (2017). 28143904 10.1126/scisignal.aaf7478 PMC5830108

[211] Malik, N. et al. Induction of lysosomal and mitochondrial biogenesis by AMPK phosphorylation of FNIP1. *Science* **380**, eabj5559 (2023). 37079666 10.1126/science.abj5559 PMC10794112

[212] Lynch, M. R. et al. TFEB-driven lysosomal biogenesis is pivotal for PGC1α-dependent renal stress resistance. *JCI Insight* **5**, e126749 (2019). 30870143 10.1172/jci.insight.126749 PMC6538327

[213] Farge, G. & Falkenberg, M. Organization of DNA in mammalian mitochondria. *Int. J. Mol. Sci.* **20**, 2770 (2019). 31195723 10.3390/ijms20112770 PMC6600607

[214] Morita, M. et al. mTORC1 controls mitochondrial activity and biogenesis through 4E-BP-dependent translational regulation. *Cell Metab.* **18**, 698–711 (2013). 24206664 10.1016/j.cmet.2013.10.001

[215] Morita, M. et al. mTOR coordinates protein synthesis, mitochondrial activity and proliferation. *Cell Cycle* **14**, 473–480 (2015). 25590164 10.4161/15384101.2014.991572 PMC4615141

[216] Chatterjee, S., Chakrabarty, Y., Banerjee, S., Ghosh, S. & Bhattacharyya, S. N. Mitochondria control mTORC1 activity-linked compartmentalization of eIF4E to regulate extracellular export of microRNAs. *J. Cell Sci.* **133**, jcs250241 (2020). 33262313 10.1242/jcs.250241

[217] Watson, A. R. et al. mTORC2 deficiency alters the metabolic profile of conventional dendritic cells. *Front. Immunol.* **10**, 1451 (2019). 31338091 10.3389/fimmu.2019.01451 PMC6626913

[218] Giacomello, M., Pyakurel, A., Glytsou, C. & Scorrano, L. The cell biology of mitochondrial membrane dynamics. *Nat. Rev. Mol. Cell Biol.* **21**, 204–224 (2020). 32071438 10.1038/s41580-020-0210-7

[219] Rambold, A. S., Kostelecky, B., Elia, N. & Lippincott-Schwartz, J. Tubular network formation protects mitochondria from autophagosomal degradation during nutrient starvation. *Proc. Natl. Acad. Sci. USA* **108**, 10190–10195 (2011). 21646527 10.1073/pnas.1107402108 PMC3121813

[220] Schrepfer, E. & Scorrano, L. Mitofusins, from mitochondria to metabolism. *Mol. Cell* **61**, 683–694 (2016). 26942673 10.1016/j.molcel.2016.02.022

[221] Karren, M. A., Coonrod, E. M., Anderson, T. K. & Shaw, J. M. The role of Fis1p-Mdv1p interactions in mitochondrial fission complex assembly. *J. Cell Biol.* **171**, 291–301 (2005). 16247028 10.1083/jcb.200506158 PMC2171191

[222] Lee, J. E., Westrate, L. M., Wu, H., Page, C. & Voeltz, G. K. Multiple dynamin family members collaborate to drive mitochondrial division. *Nature* **540**, 139–143 (2016). 27798601 10.1038/nature20555 PMC5656044

[223] Lenzi, P. et al. Rapamycin ameliorates defects in mitochondrial fission and mitophagy in glioblastoma cells. *Int. J. Mol. Sci.* **22**, 5379 (2021). 34065350 10.3390/ijms22105379 PMC8161366

[224] Zimmerman, M. A., Wilkison, S., Qi, Q., Chen, G. & Li, P. A. Mitochondrial dysfunction contributes to rapamycin-induced apoptosis of human glioblastoma cells - a synergistic effect with Temozolomide. *Int. J. Med. Sci.* **17**, 2831–2843 (2020). 33162811 10.7150/ijms.40159 PMC7645350

[225] Bravo-Sagua, R. et al. mTORC1 inhibitor rapamycin and ER stressor tunicamycin induce differential patterns of ER-mitochondria coupling. *Sci. Rep.* **6**, 1–12 (2016). 27808250 10.1038/srep36394 PMC5093439

[226] Martinez-Lopez, N. et al. mTORC2-NDRG1-CDC42 axis couples fasting to mitochondrial fission. *Nat. Cell Biol.* **25**, 989–1003 (2023). 37386153 10.1038/s41556-023-01163-3 PMC10344787

[227] Romanello, V. et al. Mitochondrial fission and remodelling contributes to muscle atrophy. *EMBO J.* **29**, 1774–1785 (2010). 20400940 10.1038/emboj.2010.60 PMC2876965

[228] Weir, H. J. et al. Dietary restriction and AMPK increase lifespan via mitochondrial network and peroxisome remodeling. *Cell Metab.* **26**, 884–896.e5 (2017). 29107506 10.1016/j.cmet.2017.09.024 PMC5718936

[229] Tan, H. W. S., Sim, A. Y. L. & Long, Y. C. Glutamine metabolism regulates autophagy-dependent mTORC1 reactivation during amino acid starvation. *Nat. Commun.* **8**, 338 (2017). 28835610 10.1038/s41467-017-00369-y PMC5569045

[230] Buel, G. R., Dang, H. Q., Asara, J. M., Blenis, J. & Mutvei, A. P. Prolonged deprivation of arginine or leucine induces PI3K/Akt-dependent reactivation of mTORC1. *J. Biol. Chem.* **298**, 102030 (2022). 35577075 10.1016/j.jbc.2022.102030 PMC9194872

[231] Yuan, Q., Chen, M., Yang, W. & Xiao, B. Circadian Rheb oscillation alters the dynamics of hepatic mTORC1 activity and mitochondrial morphology. *FEBS Lett.* **595**, 360–369 (2021). 33247956 10.1002/1873-3468.14009

[232] Morita, M. et al. mTOR controls mitochondrial dynamics and cell survival via MTFP1. *Mol. Cell* **67**, 922–935.e5 (2017). 28918902 10.1016/j.molcel.2017.08.013

[233] Tábara, L. C. et al. MTFP1 controls mitochondrial fusion to regulate inner membrane quality control and maintain mtDNA levels. *Cell* **187**, 3619–3637.e27 (2024). 38851188 10.1016/j.cell.2024.05.017

[234] Tran, Q. et al. S6 kinase 1 plays a key role in mitochondrial morphology and cellular energy flow. *Cell. Signal.* **48**, 13–24 (2018). 29673648 10.1016/j.cellsig.2018.04.002

[235] Santin, Y. et al. Oxidative stress by monoamine oxidase-A impairs transcription factor EB activation and autophagosome clearance, leading to cardiomyocyte necrosis and heart failure. *Antioxid. Redox Signal.* **25**, 10–27 (2016). 26959532 10.1089/ars.2015.6522

[236] Wang, Z., Jiang, H., Chen, S., Du, F. & Wang, X. The mitochondrial phosphatase PGAM5 functions at the convergence point of multiple necrotic death pathways. *Cell* **148**, 228–243 (2012). 22265414 10.1016/j.cell.2011.11.030

[237] Yu, B. et al. Mitochondrial phosphatase PGAM5 modulates cellular senescence by regulating mitochondrial dynamics. *Nat. Commun.* **11**, 2549 (2020). 32439975 10.1038/s41467-020-16312-7 PMC7242393

[238] Liu, P., Chang, K., Requejo, G. & Bai, H. mTORC2 protects the heart from high-fat diet-induced cardiomyopathy through mitochondrial fission in Drosophila. *Front. Cell Dev. Biol.* **10**, 866210 (2022). 35912118 10.3389/fcell.2022.866210 PMC9334792

[239] Yasuda, T., Ishihara, T., Ichimura, A. & Ishihara, N. Mitochondrial dynamics define muscle fiber type by modulating cellular metabolic pathways. *Cell Rep.* **42**, 112434 (2023). 37097817 10.1016/j.celrep.2023.112434

[240] Deus, C. M., Yambire, K. F., Oliveira, P. J. & Raimundo, N. Mitochondria-lysosome crosstalk: from physiology to neurodegeneration. *Trends Mol. Med.* **26**, 71–88 (2020). 31791731 10.1016/j.molmed.2019.10.009

[241] Ma, Y., Moors, A., Camougrand, N. & Dokudovskaya, S. The SEACIT complex is involved in the maintenance of vacuole-mitochondria contact sites and controls mitophagy. *Cell. Mol. Life Sci.* **76**, 1623–1640 (2019). 30673821 10.1007/s00018-019-03015-6 PMC11105764

[242] Lin, J.-X. et al. Rab7a-mTORC1 signaling-mediated cholesterol trafficking from the lysosome to mitochondria ameliorates hepatic lipotoxicity induced by aflatoxin B1 exposure. *Chemosphere* **320**, 138071 (2023). 36754296 10.1016/j.chemosphere.2023.138071

[243] Umanah, G. K. E. et al. AAA + ATPase Thorase inhibits mTOR signaling through the disassembly of the mTOR complex 1. *Nat. Commun.* **13**, 4836 (2022). 35977929 10.1038/s41467-022-32365-2 PMC9385847

[244] Ma, X. et al. Mitochondria-lysosome-related organelles mediate mitochondrial clearance during cellular dedifferentiation. *Cell Rep.* **42**, 113291 (2023). 37862166 10.1016/j.celrep.2023.113291 PMC10842364

[245] Rieusset, J. The role of endoplasmic reticulum-mitochondria contact sites in the control of glucose homeostasis: an update. *Cell Death Dis.* **9**, 388 (2018). 29523782 10.1038/s41419-018-0416-1 PMC5844895

[246] Depaoli, M. R., Hay, J. C., Graier, W. F. & Malli, R. The enigmatic ATP supply of the endoplasmic reticulum. *Biol. Rev. Camb. Philos. Soc.* **94**, 610–628 (2019). 30338910 10.1111/brv.12469 PMC6446729

[247] Cárdenas, C. et al. Essential regulation of cell bioenergetics by constitutive InsP3 receptor Ca2+ transfer to mitochondria. *Cell* **142**, 270–283 (2010). 20655468 10.1016/j.cell.2010.06.007 PMC2911450

[248] Vance, J. E. MAM (mitochondria-associated membranes) in mammalian cells: lipids and beyond. *Biochim. Biophys. Acta* **1841**, 595–609 (2014). 24316057 10.1016/j.bbalip.2013.11.014

[249] Verfaillie, T. et al. PERK is required at the ER-mitochondrial contact sites to convey apoptosis after ROS-based ER stress. *Cell Death Differ.* **19**, 1880–1891 (2012). 22705852 10.1038/cdd.2012.74 PMC3469056

[250] Betz, C. et al. mTOR complex 2-Akt signaling at mitochondria-associated endoplasmic reticulum membranes (MAM) regulates mitochondrial physiology. *Proc. Natl. Acad. Sci. USA* **110**, 12526–12534 (2013). 23852728 10.1073/pnas.1302455110 PMC3732980

[251] Bantug, G. R. et al. Mitochondria-endoplasmic reticulum contact sites function as immunometabolic hubs that orchestrate the rapid recall response of memory CD8+ T cells. *Immunity* **48**, 542–555.e6 (2018). 29523440 10.1016/j.immuni.2018.02.012 PMC6049611

[252] Wang, J.-D., Shao, Y., Liu, D., Liu, N.-Y. & Zhu, D.-Y. Rictor/mTORC2 involves mitochondrial function in ES cells derived cardiomyocytes via mitochondrial Connexin 43. *Acta Pharmacol. Sin.* **42**, 1790–1797 (2021). 33547375 10.1038/s41401-020-00591-3 PMC8563760

[253] Tubbs, E. et al. Mitochondria-associated endoplasmic reticulum membrane (MAM) integrity is required for insulin signaling and is implicated in hepatic insulin resistance. *Diabetes* **63**, 3279–3294 (2014). 24947355 10.2337/db13-1751

[254] Islam, M. N. et al. Mitochondrial transfer from bone-marrow-derived stromal cells to pulmonary alveoli protects against acute lung injury. *Nat. Med.* **18**, 759–765 (2012). 22504485 10.1038/nm.2736 PMC3727429

[255] Lin, X. et al. ROS/mtROS promotes TNTs formation via the PI3K/AKT/mTOR pathway to protect against mitochondrial damages in glial cells induced by engineered nanomaterials. *Part. Fibre Toxicol.* **21**, 1 (2024). 38225661 10.1186/s12989-024-00562-0 PMC10789074

[256] Huang, Z., Chen, Y. & Zhang, Y. Mitochondrial reactive oxygen species cause major oxidative mitochondrial DNA damages and repair pathways. *J. Biosci.* **45**, 84 (2020). 32661211

[257] Neklesa, T. K. & Davis, R. W. A genome-wide screen for regulators of TORC1 in response to amino acid starvation reveals a conserved Npr2/3 complex. *PLoS Genet* **5**, e1000515 (2009). 19521502 10.1371/journal.pgen.1000515 PMC2686269

[258] Torres, A. K., Fleischhart, V. & Inestrosa, N. C. Mitochondrial unfolded protein response (UPRmt): what we know thus far. *Front. Cell Dev. Biol.* **12**, 1405393 (2024). 38882057 10.3389/fcell.2024.1405393 PMC11176431

[259] Shpilka, T. & Haynes, C. M. The mitochondrial UPR: mechanisms, physiological functions and implications in ageing. *Nat. Rev. Mol. Cell Biol.* **19**, 109–120 (2018). 29165426 10.1038/nrm.2017.110

[260] Torrence, M. E. et al. The mTORC1-mediated activation of ATF4 promotes protein and glutathione synthesis downstream of growth signals. *eLife* **10**, e63326 (2021). 33646118 10.7554/eLife.63326 PMC7997658

[261] Terešak, P. et al. Regulation of PRKN-independent mitophagy. *Autophagy* **18**, 24–39 (2022). 33570005 10.1080/15548627.2021.1888244 PMC8865282

[262] Gilkerson, R. W. et al. Mitochondrial autophagy in cells with mtDNA mutations results from synergistic loss of transmembrane potential and mTORC1 inhibition. *Hum. Mol. Genet.* **21**, 978–990 (2012). 22080835 10.1093/hmg/ddr529 PMC3277306

[263] Schatton, D. et al. CLUH regulates mitochondrial metabolism by controlling translation and decay of target mRNAs. *J. Cell Biol.* **216**, 675–693 (2017). 28188211 10.1083/jcb.201607019 PMC5350512

[264] Pla-Martín, D. et al. CLUH granules coordinate translation of mitochondrial proteins with mTORC1 signaling and mitophagy. *EMBO J.* **39**, e102731 (2020). 32149416 10.15252/embj.2019102731 PMC7196838

[265] Ebrahimi-Fakhari, D. et al. Impaired mitochondrial dynamics and mitophagy in neuronal models of tuberous sclerosis complex. *Cell Rep.* **17**, 1053–1070 (2016). 27760312 10.1016/j.celrep.2016.09.054 PMC5078873

[266] Bartolomé, A. et al. mTORC1 regulates both general autophagy and mitophagy induction after oxidative phosphorylation uncoupling. *Mol. Cell. Biol.* **37**, e00441–17 (2017). 28894028 10.1128/MCB.00441-17 PMC5686580

[267] Kumar, A. & Shaha, C. SESN2 facilitates mitophagy by helping Parkin translocation through ULK1 mediated Beclin1 phosphorylation. *Sci. Rep.* **8**, 615 (2018). 29330382 10.1038/s41598-017-19102-2 PMC5766514

[268] Liu, Y. & Okamoto, K. The TORC1 signaling pathway regulates respiration-induced mitophagy in yeast. *Biochem. Biophys. Res. Commun.* **502**, 76–83 (2018). 29787763 10.1016/j.bbrc.2018.05.123

[269] Lu, C., Jiang, Y., Xu, W. & Bao, X. Sestrin2: multifaceted functions, molecular basis, and its implications in liver diseases. *Cell Death Dis.* **14**, 160 (2023). 36841824 10.1038/s41419-023-05669-4 PMC9968343

[270] Ichimura, Y. et al. Phosphorylation of p62 activates the Keap1-Nrf2 pathway during selective autophagy. *Mol. Cell* **51**, 618–631 (2013). 24011591 10.1016/j.molcel.2013.08.003

[271] Wu, X. et al. BNIP3L/NIX degradation leads to mitophagy deficiency in ischemic brains. *Autophagy* **17**, 1934–1946 (2021). 32722981 10.1080/15548627.2020.1802089 PMC8386707

[272] Liu, K. et al. BNIP3 (BCL2 interacting protein 3) regulates pluripotency by modulating mitochondrial homeostasis via mitophagy. *Cell Death Dis.* **13**, 334 (2022). 35410319 10.1038/s41419-022-04795-9 PMC9001722

[273] Poole, L. P., Bock-Hughes, A., Berardi, D. E. & Macleod, K. F. ULK1 promotes mitophagy via phosphorylation and stabilization of BNIP3. *Sci. Rep.* **11**, 20526 (2021). 34654847 10.1038/s41598-021-00170-4 PMC8519931

[274] Zhang, X., Bian, X. & Kong, J. The proapoptotic protein BNIP3 interacts with VDAC to induce mitochondrial release of endonuclease G. *PLoS One* **9**, e113642 (2014). 25436615 10.1371/journal.pone.0113642 PMC4249980

[275] Wang, W. et al. Endonuclease G promotes autophagy by suppressing mTOR signaling and activating the DNA damage response. *Nat. Commun.* **12**, 476 (2021). 33473107 10.1038/s41467-020-20780-2 PMC7817833

[276] Wang, W. et al. Cytoplasmic Endonuclease G promotes nonalcoholic fatty liver disease via mTORC2-AKT-ACLY and endoplasmic reticulum stress. *Nat. Commun.* **14**, 6201 (2023). 37794041 10.1038/s41467-023-41757-x PMC10550995

[277] Mughal, W. et al. A conserved MADS-box phosphorylation motif regulates differentiation and mitochondrial function in skeletal, cardiac, and smooth muscle cells. *Cell Death Dis.* **6**, e1944 (2015). 26512955 10.1038/cddis.2015.306 PMC5399178

[278] da Silva Rosa, S. C. et al. BNIP3L/Nix-induced mitochondrial fission, mitophagy, and impaired myocyte glucose uptake are abrogated by PRKA/PKA phosphorylation. *Autophagy* **17**, 2257–2272 (2021). 33044904 10.1080/15548627.2020.1821548 PMC8496715

[279] Melser, S. et al. Rheb regulates mitophagy induced by mitochondrial energetic status. *Cell Metab.* **17**, 719–730 (2013). 23602449 10.1016/j.cmet.2013.03.014

[280] Wu, W. et al. FUNDC1 regulates mitochondrial dynamics at the ER-mitochondrial contact site under hypoxic conditions. *EMBO J.* **35**, 1368–1384 (2016). 27145933 10.15252/embj.201593102 PMC4864280

[281] Zhao, J. et al. AP39 through AMPK-ULK1-FUNDC1 pathway regulates mitophagy, inhibits pyroptosis, and improves doxorubicin-induced myocardial fibrosis. *iScience* **27**, 109321 (2024). 38558936 10.1016/j.isci.2024.109321 PMC10981016

[282] Vargas, J. N. S. et al. Spatiotemporal control of ULK1 activation by NDP52 and TBK1 during selective autophagy. *Mol. Cell* **74**, 347–362.e6 (2019). 30853401 10.1016/j.molcel.2019.02.010 PMC6642318

[283] Bhatia-Kissova, I. & Camougrand, N. Mitophagy in yeast: decades of research. *Cells* **10**, 3541 (2021). 34944049 10.3390/cells10123541 PMC8700663

[284] Wu, X. & Tu, B. P. Selective regulation of autophagy by the Iml1-Npr2-Npr3 complex in the absence of nitrogen starvation. *Mol. Biol. Cell* **22**, 4124–4133 (2011). 21900499 10.1091/mbc.E11-06-0525 PMC3204073

[285] Bennett, C. F., Latorre-Muro, P. & Puigserver, P. Mechanisms of mitochondrial respiratory adaptation. *Nat. Rev. Mol. Cell Biol.* **23**, 817–835 (2022). 35804199 10.1038/s41580-022-00506-6 PMC9926497

[286] Makinoshima, H. et al. Signaling through the phosphatidylinositol 3-kinase (PI3K)/mammalian target of rapamycin (mTOR) axis is responsible for aerobic glycolysis mediated by glucose transporter in epidermal growth factor receptor (EGFR)-mutated lung adenocarcinoma. *J. Biol. Chem.* **290**, 17495–17504 (2015). 26023239 10.1074/jbc.M115.660498 PMC4498084

[287] Yang, T. et al. YY1 inactivated transcription coregulator PGC-1α to promote mitochondrial dysfunction of early diabetic nephropathy-associated tubulointerstitial fibrosis. *Cell Biol. Toxicol.* **39**, 391–413 (2023). 35445903 10.1007/s10565-022-09711-7

[288] Medina, D. L. et al. Lysosomal calcium signalling regulates autophagy through calcineurin and TFEB. *Nat. Cell Biol.* **17**, 288–299 (2015). 25720963 10.1038/ncb3114 PMC4801004

[289] Mansueto, G. et al. Transcription factor EB controls metabolic flexibility during exercise. *Cell Metab.* **25**, 182–196 (2017). 28011087 10.1016/j.cmet.2016.11.003 PMC5241227

[290] Fernández-Alvarez, A. J. et al. Smaug1 membrane-less organelles respond to AMPK and mTOR and affect mitochondrial function. *J. Cell Sci.* **135**, jcs253591 (2022). 34859817 10.1242/jcs.253591

[291] Pal, R., Xiong, Y. & Sardiello, M. Abnormal glycogen storage in tuberous sclerosis complex caused by impairment of mTORC1-dependent and -independent signaling pathways. *Proc. Natl. Acad. Sci. USA* **116**, 2977–2986 (2019). 30728291 10.1073/pnas.1812943116 PMC6386676

[292] Cerniglia, G. J. et al. The PI3K/Akt pathway regulates oxygen metabolism via pyruvate dehydrogenase (PDH)-E1α phosphorylation. *Mol. Cancer Ther.* **14**, 1928–1938 (2015). 25995437 10.1158/1535-7163.MCT-14-0888 PMC4529780

[293] Dutchak, P. A. et al. Loss of a negative regulator of mTORC1 induces aerobic glycolysis and altered fiber composition in skeletal muscle. *Cell Rep.* **23**, 1907–1914 (2018). 29768191 10.1016/j.celrep.2018.04.058 PMC6038807

[294] Chen, J., Sutter, B. M., Shi, L. & Tu, B. P. GATOR1 regulates nitrogenic cataplerotic reactions of the mitochondrial TCA cycle. *Nat. Chem. Biol.* **13**, 1179–1186 (2017). 28920930 10.1038/nchembio.2478 PMC5659745

[295] Valcarcel-Jimenez, L. & Frezza, C. Fumarate hydratase (FH) and cancer: a paradigm of oncometabolism. *Br. J. Cancer* **129**, 1546–1557 (2023). 37689804 10.1038/s41416-023-02412-w PMC10645937

[296] Borkum, J. M. The tricarboxylic acid cycle as a central regulator of the rate of aging: Implications for metabolic interventions. *Adv. Biol. (Weinh.)* **7**, e2300095 (2023). 37132059 10.1002/adbi.202300095

[297] Papandreou, I., Cairns, R. A., Fontana, L., Lim, A. L. & Denko, N. C. HIF-1 mediates adaptation to hypoxia by actively downregulating mitochondrial oxygen consumption. *Cell Metab.* **3**, 187–197 (2006). 16517406 10.1016/j.cmet.2006.01.012

[298] Cam, H., Easton, J. B., High, A. & Houghton, P. J. mTORC1 signaling under hypoxic conditions is controlled by ATM-dependent phosphorylation of HIF-1α. *Mol. Cell* **40**, 509–520 (2010). 21095582 10.1016/j.molcel.2010.10.030 PMC3004768

[299] He, L. et al. mTORC1 promotes metabolic reprogramming by the suppression of GSK3-dependent Foxk1 phosphorylation. *Mol. Cell* **70**, 949–960.e4 (2018). 29861159 10.1016/j.molcel.2018.04.024 PMC6591025

[300] Urbanczyk, S. et al. Mitochondrial respiration in B lymphocytes is essential for humoral immunity by controlling the flux of the TCA cycle. *Cell Rep.* **39**, 110912 (2022). 35675769 10.1016/j.celrep.2022.110912

[301] Dodd, K. M., Yang, J., Shen, M. H., Sampson, J. R. & Tee, A. R. mTORC1 drives HIF-1α and VEGF-A signalling via multiple mechanisms involving 4E-BP1, S6K1 and STAT3. *Oncogene* **34**, 2239–2250 (2015). 24931163 10.1038/onc.2014.164 PMC4172452

[302] García-Aguilar, A., Martínez-Reyes, I. & Cuezva, J. M. Changes in the turnover of the cellular proteome during metabolic reprogramming: a role for mtROS in proteostasis. *J. Proteome Res.* **18**, 3142–3155 (2019). 31293153 10.1021/acs.jproteome.9b00239

[303] Rosario, F. J. et al. Mechanistic target of rapamycin complex 1 promotes the expression of genes encoding electron transport chain proteins and stimulates oxidative phosphorylation in primary human trophoblast cells by regulating mitochondrial biogenesis. *Sci. Rep.* **9**, 246 (2019). 30670706 10.1038/s41598-018-36265-8 PMC6343003

[304] Vlahakis, A., Lopez Muniozguren, N. & Powers, T. Mitochondrial respiration links TOR complex 2 signaling to calcium regulation and autophagy. *Autophagy* **13**, 1256–1257 (2017). 28324658 10.1080/15548627.2017.1299314 PMC5529078

[305] Condon, K. J. et al. Genome-wide CRISPR screens reveal multitiered mechanisms through which mTORC1 senses mitochondrial dysfunction. *Proc. Natl. Acad. Sci. USA* **118**, e2022120118 (2021). 33483422 10.1073/pnas.2022120118 PMC7848693

[306] Balaban, R. S., Nemoto, S. & Finkel, T. Mitochondria, oxidants, and aging. *Cell* **120**, 483–495 (2005). 15734681 10.1016/j.cell.2005.02.001

[307] Tsang, C. K. et al. SOD1 phosphorylation by mTORC1 couples nutrient sensing and redox regulation. *Mol. Cell* **70**, 502–515.e8 (2018). 29727620 10.1016/j.molcel.2018.03.029 PMC6108545

[308] Ding, W.-X. et al. Nix is critical to two distinct phases of mitophagy, reactive oxygen species-mediated autophagy induction and Parkin-ubiquitin-p62-mediated mitochondrial priming. *J. Biol. Chem.* **285**, 27879–27890 (2010). 20573959 10.1074/jbc.M110.119537 PMC2934655

[309] Sarbassov, D. D. & Sabatini, D. M. Redox regulation of the nutrient-sensitive raptor-mTOR pathway and complex. *J. Biol. Chem.* **280**, 39505–39509 (2005). 16183647 10.1074/jbc.M506096200

[310] Yoshida, S. et al. Redox regulates mammalian target of rapamycin complex 1 (mTORC1) activity by modulating the TSC1/TSC2-Rheb GTPase pathway. *J. Biol. Chem.* **286**, 32651–32660 (2011). 21784859 10.1074/jbc.M111.238014 PMC3173157

[311] Bae, S. H. et al. Sestrins activate Nrf2 by promoting p62-dependent autophagic degradation of Keap1 and prevent oxidative liver damage. *Cell Metab.* **17**, 73–84 (2013). 23274085 10.1016/j.cmet.2012.12.002

[312] Scherz-Shouval, R. et al. Reactive oxygen species are essential for autophagy and specifically regulate the activity of Atg4. *EMBO J.* **26**, 1749–1760 (2007). 17347651 10.1038/sj.emboj.7601623 PMC1847657

[313] Kawagishi, H. & Finkel, T. Unraveling the truth about antioxidants: ROS and disease: finding the right balance. *Nat. Med.* **20**, 711–713 (2014). 24999942 10.1038/nm.3625

[314] Ma, Y., Silveri, L., LaCava, J. & Dokudovskaya, S. Tumor suppressor NPRL2 induces ROS production and DNA damage response. *Sci. Rep.* **7**, 15311 (2017). 29127423 10.1038/s41598-017-15497-0 PMC5681675

[315] Ding, B. et al. Sestrin2 is induced by glucose starvation via the unfolded protein response and protects cells from noncanonical necroptotic cell death. *Sci. Rep.* **6**, 22538 (2016). 26932729 10.1038/srep22538 PMC4773760

[316] Budanov, A. V., Sablina, A. A., Feinstein, E., Koonin, E. V. & Chumakov, P. M. Regeneration of peroxiredoxins by p53-regulated sestrins, homologs of bacterial AhpD. *Science* **304**, 596–600 (2004). 15105503 10.1126/science.1095569

[317] Kim, H. et al. Janus-faced Sestrin2 controls ROS and mTOR signalling through two separate functional domains. *Nat. Commun.* **6**, 10025 (2015). 26612684 10.1038/ncomms10025 PMC4674687

[318] Zhao, B. et al. Distinct role of Sesn2 in response to UVB-induced DNA damage and UVA-induced oxidative stress in melanocytes. *Photochem. Photobiol.* **93**, 375–381 (2017). 27463837 10.1111/php.12624 PMC5272907

[319] Iskandar, K. et al. A novel MTORC2-AKT-ROS axis triggers mitofission and mitophagy-associated execution of colorectal cancer cells upon drug-induced activation of mutant KRAS. *Autophagy* **20**, 1418–1441 (2024). 38261660 10.1080/15548627.2024.2307224 PMC11210925

[320] Rodriguez-Vargas, J.-M. et al. Parp3 promotes astrocytic differentiation through a tight regulation of Nox4-induced ROS and mTorc2 activation. *Cell Death Dis.* **11**, 954 (2020). 33159039 10.1038/s41419-020-03167-5 PMC7648797

[321] Ali, E. S. & Ben-Sahra, I. Regulation of nucleotide metabolism in cancers and immune disorders. *Trends Cell Biol.* **33**, 950–966 (2023). 36967301 10.1016/j.tcb.2023.03.003 PMC10518033

[322] Sato, T. et al. Rheb protein binds CAD (carbamoyl-phosphate synthetase 2, aspartate transcarbamoylase, and dihydroorotase) protein in a GTP- and effector domain-dependent manner and influences its cellular localization and carbamoyl-phosphate synthetase (CPSase) activity. *J. Biol. Chem.* **290**, 1096–1105 (2015). 25422319 10.1074/jbc.M114.592402 PMC4294477

[323] Nakashima, A. et al. Association of CAD, a multifunctional protein involved in pyrimidine synthesis, with mLST8, a component of the mTOR complexes. *J. Biomed. Sci.* **20**, 24 (2013). 23594158 10.1186/1423-0127-20-24 PMC3639846

[324] Gonzalez Herrera, K. N. et al. Small-molecule screen identifies de novo nucleotide synthesis as a vulnerability of cells lacking SIRT3. *Cell Rep.* **22**, 1945–1955 (2018). 29466723 10.1016/j.celrep.2018.01.076 PMC5902027

[325] Wu, J. et al. Metabolic determinants of germinal center B-cell formation and responses. *Nat. Chem. Biol.* **21**, 371–382 (2025). 39060389 10.1038/s41589-024-01690-6

[326] French, J. B. et al. Spatial colocalization and functional link of purinosomes with mitochondria. *Science* **351**, 733–737 (2016). 26912862 10.1126/science.aac6054 PMC4881839

[327] Xie, Y., Lei, X., Zhao, G., Guo, R. & Cui, N. mTOR in programmed cell death and its therapeutic implications. *Cytokine Growth Factor Rev.* **71–72**, 66–81 (2023). 37380596 10.1016/j.cytogfr.2023.06.002

[328] Wolf, P., Schoeniger, A. & Edlich, F. Pro-apoptotic complexes of BAX and BAK on the outer mitochondrial membrane. *Biochim. Biophys. Acta Mol. Cell Res.* **1869**, 119317 (2022). 35752202 10.1016/j.bbamcr.2022.119317

[329] Bock, F. J. & Tait, S. W. G. Mitochondria as multifaceted regulators of cell death. *Nat. Rev. Mol. Cell Biol.* **21**, 85–100 (2020). 31636403 10.1038/s41580-019-0173-8

[330] Vringer, E. & Tait, S. W. G. Mitochondria and cell death-associated inflammation. *Cell Death Differ.* **30**, 304–312 (2023). 36447047 10.1038/s41418-022-01094-w PMC9950460

[331] Hu, X. et al. Dual PI3K/mTOR inhibitor PKI-402 suppresses the growth of ovarian cancer cells by degradation of Mcl-1 through autophagy. *Biomed. Pharmacother.* **129**, 110397 (2020). 32585451 10.1016/j.biopha.2020.110397

[332] Gao, M. et al. Role of mitochondria in ferroptosis. *Mol. Cell* **73**, 354–363.e3 (2019). 30581146 10.1016/j.molcel.2018.10.042 PMC6338496

[333] Zhang, Y. et al. mTORC1 couples cyst(e)ine availability with GPX4 protein synthesis and ferroptosis regulation. *Nat. Commun.* **12**, 1589 (2021). 33707434 10.1038/s41467-021-21841-w PMC7952727

[334] Cosialls, E. et al. mTOR inhibition suppresses salinomycin-induced ferroptosis in breast cancer stem cells by ironing out mitochondrial dysfunctions. *Cell Death Dis.* **14**, 744 (2023). 37968262 10.1038/s41419-023-06262-5 PMC10651934

[335] Tsvetkov, P. et al. Copper induces cell death by targeting lipoylated TCA cycle proteins. *Science* **375**, 1254–1261 (2022). 35298263 10.1126/science.abf0529 PMC9273333

[336] Wen, H. et al. Cuproptosis enhances docetaxel chemosensitivity by inhibiting autophagy via the DLAT/mTOR pathway in prostate cancer. *FASEB J.* **37**, e23145 (2023). 37584654 10.1096/fj.202300980R

[337] Yu, P. et al. Pyroptosis: mechanisms and diseases. *Signal Transduct. Target. Ther.* **6**, 1–21 (2021). 33776057 10.1038/s41392-021-00507-5 PMC8005494

[338] Li, M. et al. Adrenomedullin alleviates the pyroptosis of Leydig cells by promoting autophagy via the ROS–AMPK–mTOR axis. *Cell Death Dis.* **10**, 489 (2019). 31222000 10.1038/s41419-019-1728-5 PMC6586845

[339] Yao, R. et al. Pathogenic effects of inhibition of mTORC1/STAT3 axis facilitates *Staphylococcus aureus*-induced pyroptosis in human macrophages. *Cell Commun. Signal.* **18**, 187 (2020). 33256738 10.1186/s12964-020-00677-9 PMC7706204

[340] Zhao, G., Xie, Y., Lei, X., Guo, R. & Cui, N. mTOR aggravated CD4+ T-cell pyroptosis by regulating the PPARγ-Nrf2 pathway in sepsis. *Int. Immunopharmacol.* **140**, 112822 (2024). 39096877 10.1016/j.intimp.2024.112822

[341] Koo, S.-H. et al. The CREB coactivator TORC2 is a key regulator of fasting glucose metabolism. *Nature* **437**, 1109–1111 (2005). 16148943 10.1038/nature03967

[342] Um, S. H. et al. Absence of S6K1 protects against age- and diet-induced obesity while enhancing insulin sensitivity. *Nature* **431**, 200–205 (2004). 15306821 10.1038/nature02866

[343] Sengupta, S., Peterson, T. R., Laplante, M., Oh, S. & Sabatini, D. M. mTORC1 controls fasting-induced ketogenesis and its modulation by ageing. *Nature* **468**, 1100–1104 (2010). 21179166 10.1038/nature09584

[344] Komatsu, M. et al. Impairment of starvation-induced and constitutive autophagy in Atg7-deficient mice. *J. Cell Biol.* **169**, 425–434 (2005). 15866887 10.1083/jcb.200412022 PMC2171928

[345] Lamming, D. W. et al. Rapamycin-induced insulin resistance is mediated by mTORC2 loss and uncoupled from longevity. *Science* **335**, 1638–1643 (2012). 22461615 10.1126/science.1215135 PMC3324089

[346] Hagiwara, A. et al. Hepatic mTORC2 activates glycolysis and lipogenesis through Akt, glucokinase, and SREBP1c. *Cell Metab.* **15**, 725–738 (2012). 22521878 10.1016/j.cmet.2012.03.015

[347] Biddinger, S. B. et al. Hepatic insulin resistance is sufficient to produce dyslipidemia and susceptibility to atherosclerosis. *Cell Metab.* **7**, 125–134 (2008). 18249172 10.1016/j.cmet.2007.11.013 PMC4251554

[348] Kenerson, H. L., Yeh, M. M. & Yeung, R. S. Tuberous sclerosis complex-1 deficiency attenuates diet-induced hepatic lipid accumulation. *PLoS One* **6**, e18075 (2011). 21479224 10.1371/journal.pone.0018075 PMC3066210

[349] Yecies, J. L. et al. Akt stimulates hepatic SREBP1c and lipogenesis through parallel mTORC1-dependent and independent pathways. *Cell Metab.* **14**, 21–32 (2011). 21723501 10.1016/j.cmet.2011.06.002 PMC3652544

[350] Yuan, M., Pino, E., Wu, L., Kacergis, M. & Soukas, A. A. Identification of Akt-independent regulation of hepatic lipogenesis by mammalian target of rapamycin (mTOR) complex 2. *J. Biol. Chem.* **287**, 29579–29588 (2012). 22773877 10.1074/jbc.M112.386854 PMC3436168

[351] Efeyan, A. et al. Regulation of mTORC1 by the Rag GTPases is necessary for neonatal autophagy and survival. *Nature* **493**, 679–683 (2013). 23263183 10.1038/nature11745 PMC4000705

[352] Peng, M., Yin, N. & Li, M. O. Sestrins function as guanine nucleotide dissociation inhibitors for Rag GTPases to control mTORC1 signaling. *Cell* **159**, 122–133 (2014). 25259925 10.1016/j.cell.2014.08.038 PMC4181352

[353] Kuma, A. et al. The role of autophagy during the early neonatal starvation period. *Nature* **432**, 1032–1036 (2004). 15525940 10.1038/nature03029

[354] Risson, V. et al. Muscle inactivation of mTOR causes metabolic and dystrophin defects leading to severe myopathy. *J. Cell Biol.* **187**, 859–874 (2009). 20008564 10.1083/jcb.200903131 PMC2806319

[355] Bentzinger, C. F. et al. Skeletal muscle-specific ablation of raptor, but not of rictor, causes metabolic changes and results in muscle dystrophy. *Cell Metab.* **8**, 411–424 (2008). 19046572 10.1016/j.cmet.2008.10.002

[356] Zhang, D. et al. MTORC1 regulates cardiac function and myocyte survival through 4E-BP1 inhibition in mice. *J. Clin. Invest.* **120**, 2805–2816 (2010). 20644257 10.1172/JCI43008 PMC2912201

[357] Shende, P. et al. Cardiac raptor ablation impairs adaptive hypertrophy, alters metabolic gene expression, and causes heart failure in mice. *Circulation* **123**, 1073–1082 (2011). 21357822 10.1161/CIRCULATIONAHA.110.977066

[358] Polak, P. et al. Adipose-specific knockout of raptor results in lean mice with enhanced mitochondrial respiration. *Cell Metab.* **8**, 399–410 (2008). 19046571 10.1016/j.cmet.2008.09.003

[359] El-Chaâr, D., Gagnon, A. & Sorisky, A. Inhibition of insulin signaling and adipogenesis by rapamycin: effect on phosphorylation of p70 S6 kinase vs eIF4E-BP1. *Int. J. Obes. Relat. Metab. Disord.* **28**, 191–198 (2004). 14970836 10.1038/sj.ijo.0802554

[360] Kim, J. E. & Chen, J. regulation of peroxisome proliferator-activated receptor-gamma activity by mammalian target of rapamycin and amino acids in adipogenesis. *Diabetes* **53**, 2748–2756 (2004). 15504954 10.2337/diabetes.53.11.2748

[361] Carnevalli, L. S. et al. S6K1 plays a critical role in early adipocyte differentiation. *Dev. Cell* **18**, 763–774 (2010). 20493810 10.1016/j.devcel.2010.02.018 PMC2918254

[362] Le Bacquer, O. et al. Elevated sensitivity to diet-induced obesity and insulin resistance in mice lacking 4E-BP1 and 4E-BP2. *J. Clin. Invest.* **117**, 387–396 (2007). 17273556 10.1172/JCI29528 PMC1783830

[363] Zhang, H. H. et al. Insulin stimulates adipogenesis through the Akt-TSC2-mTORC1 pathway. *PLoS One* **4**, e6189 (2009). 19593385 10.1371/journal.pone.0006189 PMC2703782

[364] Cybulski, N., Polak, P., Auwerx, J., Rüegg, M. A. & Hall, M. N. mTOR complex 2 in adipose tissue negatively controls whole-body growth. *Proc. Natl. Acad. Sci. USA* **106**, 9902–9907 (2009). 19497867 10.1073/pnas.0811321106 PMC2700987

[365] Powell, J. D., Pollizzi, K. N., Heikamp, E. B. & Horton, M. R. Regulation of immune responses by mTOR. *Annu. Rev. Immunol.* **30**, 39–68 (2012). 22136167 10.1146/annurev-immunol-020711-075024 PMC3616892

[366] Delgoffe, G. M. et al. The mTOR kinase differentially regulates effector and regulatory T-cell lineage commitment. *Immunity* **30**, 832–844 (2009). 19538929 10.1016/j.immuni.2009.04.014 PMC2768135

[367] Yang, K. et al. T-cell exit from quiescence and differentiation into Th2 cells depend on Raptor-mTORC1-mediated metabolic reprogramming. *Immunity* **39**, 1043–1056 (2013). 24315998 10.1016/j.immuni.2013.09.015 PMC3986063

[368] Delgoffe, G. M. et al. The kinase mTOR regulates the differentiation of helper T cells through the selective activation of signaling by mTORC1 and mTORC2. *Nat. Immunol.* **12**, 295–303 (2011). 21358638 10.1038/ni.2005 PMC3077821

[369] Park, Y. et al. TSC1 regulates the balance between effector and regulatory T cells. *J. Clin. Invest.* **123**, 5165–5178 (2013). 24270422 10.1172/JCI69751 PMC3859395

[370] Sinclair, L. V. et al. Control of amino-acid transport by antigen receptors coordinates the metabolic reprogramming essential for T-cell differentiation. *Nat. Immunol.* **14**, 500–508 (2013). 23525088 10.1038/ni.2556 PMC3672957

[371] Nakaya, M. et al. Inflammatory T-cell responses rely on amino acid transporter ASCT2 facilitation of glutamine uptake and mTORC1 kinase activation. *Immunity* **40**, 692–705 (2014). 24792914 10.1016/j.immuni.2014.04.007 PMC4074507

[372] Araki, K. et al. mTOR regulates memory CD8 T-cell differentiation. *Nature* **460**, 108–112 (2009). 19543266 10.1038/nature08155 PMC2710807

[373] Rao, R. R., Li, Q., Odunsi, K. & Shrikant, P. A. The mTOR kinase determines effector versus memory CD8+ T-cell fate by regulating the expression of transcription factors T-bet and Eomesodermin. *Immunity* **32**, 67–78 (2010). 20060330 10.1016/j.immuni.2009.10.010 PMC5836496

[374] Zeng, H. et al. mTORC1 couples immune signals and metabolic programming to establish T(reg)-cell function. *Nature* **499**, 485–490 (2013). 23812589 10.1038/nature12297 PMC3759242

[375] Battaglia, M., Stabilini, A. & Roncarolo, M.-G. Rapamycin selectively expands CD4+CD25+FoxP3+ regulatory T cells. *Blood* **105**, 4743–4748 (2005). 15746082 10.1182/blood-2004-10-3932

[376] De Rosa, V. et al. Glycolysis controls the induction of human regulatory T cells by modulating the expression of FOXP3 exon 2 splicing variants. *Nat. Immunol.* **16**, 1174–1184 (2015). 26414764 10.1038/ni.3269 PMC4868085

[377] Gerriets, V. A. et al. Foxp3 and Toll-like receptor signaling balance Treg cell anabolic metabolism for suppression. *Nat. Immunol.* **17**, 1459–1466 (2016). 27695003 10.1038/ni.3577 PMC5215903

[378] Wei, J. et al. Autophagy enforces functional integrity of regulatory T cells by coupling environmental cues and metabolic homeostasis. *Nat. Immunol.* **17**, 277–285 (2016). 26808230 10.1038/ni.3365 PMC4755832

[379] Iwata, T. N. et al. Conditional disruption of Raptor reveals an essential role for mTORC1 in B-cell development, survival, and metabolism. *J. Immunol.* **197**, 2250–2260 (2016). 27521345 10.4049/jimmunol.1600492 PMC5009877

[380] Raybuck, A. L. et al. B-cell-intrinsic mTORC1 promotes germinal center-defining transcription factor gene expression, somatic hypermutation, and memory B-Cell generation in humoral immunity. *J. Immunol.* **200**, 2627–2639 (2018). 29531165 10.4049/jimmunol.1701321 PMC5893413

[381] Breda, C. N. et al. Mitochondria as central hub of the immune system. *Redox Biol.* **26**, 101255 (2019). 31247505 10.1016/j.redox.2019.101255 PMC6598836

[382] Jones, R. G. & Pearce, E. J. MenTORing immunity: mTOR signaling in the development and function of tissue-resident immune cells. *Immunity* **46**, 730–742 (2017). 28514674 10.1016/j.immuni.2017.04.028 PMC5695239

[383] Zhu, X. et al. The nutrient-sensing Rag-GTPase complex in B cells controls humoral immunity via TFEB/TFE3-dependent mitochondrial fitness. *Nat. Commun.* **15**, 10163 (2024). 39580479 10.1038/s41467-024-54344-5 PMC11585635

[384] Riley, J. S. & Tait, S. W. Mitochondrial DNA in inflammation and immunity. *EMBO Rep.* **21**, e49799 (2020). 32202065 10.15252/embr.201949799 PMC7132203

[385] Zhang, Q. et al. Circulating mitochondrial DAMPs cause inflammatory responses to injury. *Nature* **464**, 104–107 (2010). 20203610 10.1038/nature08780 PMC2843437

[386] Hu, M.-M. & Shu, H.-B. Mitochondrial DNA-triggered innate immune response: mechanisms and diseases. *Cell. Mol. Immunol.* **20**, 1403–1412 (2023). 37932533 10.1038/s41423-023-01086-x PMC10687031

[387] Jiménez-Loygorri, J. I. et al. Mitophagy curtails cytosolic mtDNA-dependent activation of cGAS/STING inflammation during aging. *Nat. Commun.* **15**, 830 (2024). 38280852 10.1038/s41467-024-45044-1 PMC10821893

[388] Totiger, T. M. et al. Urolithin A, a novel natural compound to target PI3K/AKT/mTOR pathway in pancreatic cancer. *Mol. Cancer Ther.* **18**, 301–311 (2019). 30404927 10.1158/1535-7163.MCT-18-0464 PMC6363854

[389] Xu, Y. et al. The cGAS-STING pathway activates transcription factor TFEB to stimulate lysosome biogenesis and pathogen clearance. *Immunity* **58**, 309–325.e6 (2025). 39689715 10.1016/j.immuni.2024.11.017

[390] Dvorkin, S., Cambier, S., Volkman, H. E. & Stetson, D. B. New frontiers in the cGAS-STING intracellular DNA-sensing pathway. *Immunity* **57**, 718–730 (2024). 38599167 10.1016/j.immuni.2024.02.019 PMC11013568

[391] Gkirtzimanaki, K. et al. IFNα impairs autophagic degradation of mtDNA promoting autoreactivity of SLE monocytes in a STING-dependent fashion. *Cell Rep.* **25**, 921–933.e5 (2018). 30355498 10.1016/j.celrep.2018.09.001 PMC6218203

[392] Ip, W. K. E., Hoshi, N., Shouval, D. S., Snapper, S. & Medzhitov, R. Anti-inflammatory effect of IL-10 mediated by metabolic reprogramming of macrophages. *Science* **356**, 513–519 (2017). 28473584 10.1126/science.aal3535 PMC6260791

[393] Kaeberlein, M. et al. Regulation of yeast replicative life span by TOR and Sch9 in response to nutrients. *Science* **310**, 1193–1196 (2005). 16293764 10.1126/science.1115535

[394] Vellai, T. et al. Genetics: influence of TOR kinase on lifespan in *C. elegans*. *Nature* **426**, 620 (2003). 14668850 10.1038/426620a

[395] Kapahi, P. et al. Regulation of lifespan in Drosophila by modulation of genes in the TOR signaling pathway. *Curr. Biol.* **14**, 885–890 (2004). 15186745 10.1016/j.cub.2004.03.059 PMC2754830

[396] Harrison, D. E. et al. Rapamycin fed late in life extends lifespan in genetically heterogeneous mice. *Nature* **460**, 392–395 (2009). 19587680 10.1038/nature08221 PMC2786175

[397] Bitto, A. et al. Transient rapamycin treatment can increase lifespan and healthspan in middle-aged mice. *eLife* **5**, e16351 (2016). 27549339 10.7554/eLife.16351 PMC4996648

[398] Fernández, ÁF. et al. Disruption of the beclin 1-BCL2 autophagy regulatory complex promotes longevity in mice. *Nature* **558**, 136–140 (2018). 29849149 10.1038/s41586-018-0162-7 PMC5992097

[399] Hansen, M. et al. A role for autophagy in the extension of lifespan by dietary restriction in *C. elegans*. *PLoS Genet* **4**, e24 (2008). 18282106 10.1371/journal.pgen.0040024 PMC2242811

[400] Hansen, M. et al. Lifespan extension by conditions that inhibit translation in *Caenorhabditis elegans*. *Aging Cell* **6**, 95–110 (2007). 17266679 10.1111/j.1474-9726.2006.00267.x

[401] Selman, C. et al. Ribosomal protein S6 kinase 1 signaling regulates mammalian life span. *Science* **326**, 140–144 (2009). 19797661 10.1126/science.1177221 PMC4954603

[402] Ferrara-Romeo, I. et al. The mTOR pathway is necessary for survival of mice with short telomeres. *Nat. Commun.* **11**, 1168 (2020). 32127537 10.1038/s41467-020-14962-1 PMC7054554

[403] Miwa, S. et al. Decreased mTOR signalling reduces mitochondrial ROS in brain via accumulation of the telomerase protein TERT within mitochondria. *Aging (Albany NY)* **8**, 2551–2564 (2016). 27777385 10.18632/aging.101089 PMC5115906

[404] Chen, G., Kroemer, G. & Kepp, O. Mitophagy: an emerging role in aging and age-associated diseases. *Front. Cell Dev. Biol.* **8**, 200 (2020). 32274386 10.3389/fcell.2020.00200 PMC7113588

[405] López-Otín, C., Blasco, M. A., Partridge, L., Serrano, M. & Kroemer, G. Hallmarks of aging: An expanding universe. *Cell* **186**, 243–278 (2023). 36599349 10.1016/j.cell.2022.11.001

[406] Mannick, J. B. & Lamming, D. W. Targeting the biology of aging with mTOR inhibitors. *Nat. Aging* **3**, 642–660 (2023). 37142830 10.1038/s43587-023-00416-y PMC10330278

[407] Bielas, J. et al. Long term rapamycin treatment improves mitochondrial DNA quality in aging mice. *Exp. Gerontol.* **106**, 125–131 (2018). 29486228 10.1016/j.exger.2018.02.021 PMC5911406

[408] Chen, C., Liu, Y., Liu, Y. & Zheng, P. mTOR regulation and therapeutic rejuvenation of aging hematopoietic stem cells. *Sci. Signal.* **2**, ra75 (2009). 19934433 10.1126/scisignal.2000559 PMC4020596

[409] Carroll, B. et al. Persistent mTORC1 signaling in cell senescence results from defects in amino acid and growth factor sensing. *J. Cell Biol.* **216**, 1949–1957 (2017). 28566325 10.1083/jcb.201610113 PMC5496614

[410] Herranz, N. et al. mTOR regulates MAPKAPK2 translation to control the senescence-associated secretory phenotype. *Nat. Cell Biol.* **17**, 1205–1217 (2015). 26280535 10.1038/ncb3225 PMC4589897

[411] Arriola Apelo, S. I., Pumper, C. P., Baar, E. L., Cummings, N. E. & Lamming, D. W. Intermittent administration of rapamycin extends the life span of female C57BL/6J mice. *J. Gerontol. A. Biol. Sci. Med. Sci.* **71**, 876–881 (2016). 27091134 10.1093/gerona/glw064 PMC4906329

[412] Mannick, J. B. et al. TORC1 inhibition enhances immune function and reduces infections in the elderly. *Sci. Transl. Med.* **10**, eaaq1564 (2018). 29997249 10.1126/scitranslmed.aaq1564

[413] Kaeberlein, T. L. et al. Evaluation of off-label rapamycin use to promote healthspan in 333 adults. *Geroscience* **45**, 2757–2768 (2023). 37191826 10.1007/s11357-023-00818-1 PMC10187519

[414] Iida, T. & Lilly, M. A. missing oocyte encodes a highly conserved nuclear protein required for the maintenance of the meiotic cycle and oocyte identity in Drosophila. *Development* **131**, 1029–1039 (2004). 14973288 10.1242/dev.01001

[415] Cai, W., Wei, Y., Jarnik, M., Reich, J. & Lilly, M. A. The GATOR2 component Wdr24 regulates TORC1 activity and lysosome function. *PLoS Genet* **12**, e1006036 (2016). 27166823 10.1371/journal.pgen.1006036 PMC4864241

[416] Zhu, H., Shen, H., Sewell, A. K., Kniazeva, M. & Han, M. A novel sphingolipid-TORC1 pathway critically promotes postembryonic development in *Caenorhabditis elegans*. *eLife* **2**, e00429 (2013). 23705068 10.7554/eLife.00429 PMC3660743

[417] Qi, B., Kniazeva, M. & Han, M. A vitamin-B2-sensing mechanism that regulates gut protease activity to impact animal’s food behavior and growth. *eLife* **6**, e26243 (2017). 28569665 10.7554/eLife.26243 PMC5478268

[418] Iyer, D. P. et al. mTOR activity paces human blastocyst stage developmental progression. *Cell* **187**, 6566–6583.e22 (2024). 39332412 10.1016/j.cell.2024.08.048 PMC7617234

[419] Xie, M. et al. The level of protein in the maternal murine diet modulates the facial appearance of the offspring via mTORC1 signaling. *Nat. Commun.* **15**, 2367 (2024). 38531868 10.1038/s41467-024-46030-3 PMC10965948

[420] Zhulyn, O. et al. Evolutionarily divergent mTOR remodels translatome for tissue regeneration. *Nature* **620**, 163–171 (2023). 37495694 10.1038/s41586-023-06365-1 PMC11181899

[421] Marcondes-de-Castro, I. A., Reis-Barbosa, P. H., Marinho, T. S., Aguila, M. B. & Mandarim-de-Lacerda, C. A. AMPK/mTOR pathway significance in healthy liver and nonalcoholic fatty liver disease and its progression. *J. Gastroenterol. Hepatol.* **38**, 1868–1876 (2023). 37438882 10.1111/jgh.16272

[422] Crino, P. B. The mTOR signalling cascade: paving new roads to cure neurological disease. *Nat. Rev. Neurol.* **12**, 379–392 (2016). 27340022 10.1038/nrneurol.2016.81

[423] Iffland, P. H., Carson, V., Bordey, A. & Crino, P. B. GATORopathies: The role of amino acid regulatory gene mutations in epilepsy and cortical malformations. *Epilepsia* **60**, 2163–2173 (2019). 31625153 10.1111/epi.16370 PMC7155771

[424] Burger, B. J. et al. Autistic siblings with novel mutations in two different genes: insight for genetic workups of autistic siblings and connection to mitochondrial dysfunction. *Front. Pediatr.* **5**, 219 (2017). 29075622 10.3389/fped.2017.00219 PMC5643424

[425] Fang, Y. et al. Opposing functions of β-arrestin 1 and 2 in Parkinson’s disease via microglia inflammation and Nprl3. *Cell Death Differ.* **28**, 1822–1836 (2021). 33686256 10.1038/s41418-020-00704-9 PMC8184754

[426] Sambri, I., Ferniani, M. & Ballabio, A. Ragopathies and the rising influence of RagGTPases on human diseases. *Nat. Commun.* **15**, 5812 (2024). 38987251 10.1038/s41467-024-50034-4 PMC11237164

[427] Chen, J.-H. et al. Mutations of RagA GTPase in mTORC1 pathway are associated with autosomal dominant cataracts. *PLoS Genet* **12**, e1006090 (2016). 27294265 10.1371/journal.pgen.1006090 PMC4905677

[428] Long, P. A. et al. De novo RRAGC mutation activates mTORC1 signaling in syndromic fetal dilated cardiomyopathy. *Hum. Genet.* **135**, 909–917 (2016). 27234373 10.1007/s00439-016-1685-3 PMC4947566

[429] Schlingmann, K. P. et al. mTOR-activating mutations in RRAGD are causative for kidney tubulopathy and cardiomyopathy. *J. Am. Soc. Nephrol.* **32**, 2885–2899 (2021). 34607910 10.1681/ASN.2021030333 PMC8806087

[430] Sambri, I. et al. RagD autoactivating mutations impair MiT/TFE activity in kidney tubulopathy and cardiomyopathy syndrome. *Nat. Commun.* **14**, 2775 (2023). 37188688 10.1038/s41467-023-38428-2 PMC10185561

[431] Okosun, J. et al. Recurrent mTORC1-activating RRAGC mutations in follicular lymphoma. *Nat. Genet.* **48**, 183–188 (2016). 26691987 10.1038/ng.3473 PMC4731318

[432] Tian, T., Li, X. & Zhang, J. mTOR signaling in cancer and mTOR inhibitors in solid tumor targeting therapy. *Int. J. Mol. Sci.* **20**, 755 (2019). 30754640 10.3390/ijms20030755 PMC6387042

[433] Grabiner, B. C. et al. A diverse array of cancer-associated MTOR mutations are hyperactivating and can predict rapamycin sensitivity. *Cancer Discov.* **4**, 554–563 (2014). 24631838 10.1158/2159-8290.CD-13-0929 PMC4012430

[434] Cheng, H. et al. RICTOR amplification defines a novel subset of patients with lung cancer who may benefit from treatment with mTORC1/2 inhibitors. *Cancer Discov.* **5**, 1262–1270 (2015). 26370156 10.1158/2159-8290.CD-14-0971 PMC4670806

[435] Morrison Joly, M. et al. Rictor/mTORC2 drives progression and therapeutic resistance of HER2-amplified breast cancers. *Cancer Res* **76**, 4752–4764 (2016). 27197158 10.1158/0008-5472.CAN-15-3393 PMC5758362

[436] El Shamieh, S., Saleh, F., Moussa, S., Kattan, J. & Farhat, F. RICTOR gene amplification is correlated with metastasis and therapeutic resistance in triple-negative breast cancer. *Pharmacogenomics* **19**, 757–760 (2018). 29790419 10.2217/pgs-2018-0019

[437] Guertin, D. A. et al. mTOR complex 2 is required for the development of prostate cancer induced by Pten loss in mice. *Cancer Cell* **15**, 148–159 (2009). 19185849 10.1016/j.ccr.2008.12.017 PMC2701381

[438] Sengupta, S., Ghufran, S. M., Khan, A., Biswas, S. & Roychoudhury, S. Transition of amyloid/mutant p53 from tumor suppressor to an oncogene and therapeutic approaches to ameliorate metastasis and cancer stemness. *Cancer Cell Int* **22**, 416 (2022). 36567312 10.1186/s12935-022-02831-4 PMC9791775

[439] DeGraffenried, L. A. et al. Reduced PTEN expression in breast cancer cells confers susceptibility to inhibitors of the PI3 kinase/Akt pathway. *Ann. Oncol.* **15**, 1510–1516 (2004). 15367412 10.1093/annonc/mdh388

[440] Sjödahl, G. et al. A systematic study of gene mutations in urothelial carcinoma; inactivating mutations in TSC2 and PIK3R1. *PLoS One* **6**, e18583 (2011). 21533174 10.1371/journal.pone.0018583 PMC3077383

[441] Kwiatkowski, D. J. et al. A mouse model of TSC1 reveals sex-dependent lethality from liver hemangiomas, and upregulation of p70S6 kinase activity in Tsc1 null cells. *Hum. Mol. Genet.* **11**, 525–534 (2002). 11875047 10.1093/hmg/11.5.525

[442] Gao, Y. et al. Rheb1 promotes tumor progression through mTORC1 in MLL-AF9-initiated murine acute myeloid leukemia. *J. Hematol. Oncol.* **9**, 36 (2016). 27071307 10.1186/s13045-016-0264-3 PMC4830070

[443] Nickerson, M. L. et al. Mutations in a novel gene lead to kidney tumors, lung wall defects, and benign tumors of the hair follicle in patients with the Birt-Hogg-Dubé syndrome. *Cancer Cell* **2**, 157–164 (2002). 12204536 10.1016/s1535-6108(02)00104-6

[444] Solanki, S. et al. Dysregulated amino acid sensing drives colorectal cancer growth and metabolic reprogramming leading to chemoresistance. *Gastroenterology* **164**, 376–391.e13 (2023). 36410445 10.1053/j.gastro.2022.11.014 PMC10448739

[445] Ueda, K. et al. The 3p21.3 tumor suppressor NPRL2 plays an important role in cisplatin-induced resistance in human non-small cell lung cancer cells. *Cancer Res* **66**, 9682–9690 (2006). 17018626 10.1158/0008-5472.CAN-06-1483

[446] Fendt, S.-M. 100 years of the Warburg effect: A cancer metabolism endeavor. *Cell* **187**, 3824–3828 (2024). 39059359 10.1016/j.cell.2024.06.026

[447] Guerra, F. et al. Mitochondrial dysfunction: A novel potential driver of epithelial-to-mesenchymal transition in cancer. *Front. Oncol.* **7**, 295 (2017). 29250487 10.3389/fonc.2017.00295 PMC5716985

[448] Ashraf, R. & Kumar, S. Mfn2-mediated mitochondrial fusion promotes autophagy and suppresses ovarian cancer progression by reducing ROS through AMPK/mTOR/ERK signaling. *Cell. Mol. Life Sci.* **79**, 573 (2022). 36308626 10.1007/s00018-022-04595-6 PMC11803038

[449] Zheng, X. et al. Mitochondrial fragmentation limits NK cell-based tumor immunosurveillance. *Nat. Immunol.* **20**, 1656–1667 (2019). 31636463 10.1038/s41590-019-0511-1

[450] Lanzetti, L. Oncometabolites at the crossroads of genetic, epigenetic and ecological alterations in cancer. *Cell Death Differ.* **31**, 1582–1594 (2024). 39438765 10.1038/s41418-024-01402-6 PMC11618380

[451] Drusian, L. et al. mTORC1 upregulation leads to accumulation of the oncometabolite fumarate in a mouse model of renal cell carcinoma. *Cell Rep.* **24**, 1093–1104.e6 (2018). 30067967 10.1016/j.celrep.2018.06.106

[452] Madala, H. R. et al. Nitrogen trapping as a therapeutic strategy in tumors with mitochondrial dysfunction. *Cancer Res* **80**, 3492–3506 (2020). 32651261 10.1158/0008-5472.CAN-20-0246 PMC7484159

[453] Liu, N. et al. Lactate inhibits ATP6V0d2 expression in tumor-associated macrophages to promote HIF-2α-mediated tumor progression. *J. Clin. Invest.* **129**, 631–646 (2019). 30431439 10.1172/JCI123027 PMC6355226

[454] Carbonneau, M. et al. The oncometabolite 2-hydroxyglutarate activates the mTOR signalling pathway. *Nat. Commun.* **7**, 12700 (2016). 27624942 10.1038/ncomms12700 PMC5027283

[455] Krall, A. S. et al. Asparagine couples mitochondrial respiration to ATF4 activity and tumor growth. *Cell Metab.* **33**, 1013–1026.e6 (2021). 33609439 10.1016/j.cmet.2021.02.001 PMC8102379

[456] Tong, M. et al. Loss of tyrosine catabolic enzyme HPD promotes glutamine anaplerosis through mTOR signaling in liver cancer. *Cell Rep.* **36**, 109617 (2021). 34433044 10.1016/j.celrep.2021.109617

[457] Lu, J. et al. Multiomics reveals clinically relevant proliferative drive associated with mTOR-MYC-OXPHOS activity in chronic lymphocytic leukemia. *Nat. Cancer* **2**, 853–864 (2021). 34423310 10.1038/s43018-021-00216-6 PMC7611543

[458] Kielbassa, K. et al. Ibrutinib sensitizes CLL cells to venetoclax by interrupting TLR9-induced CD40 upregulation and protein translation. *Leukemia* **37**, 1268–1276 (2023). 37100883 10.1038/s41375-023-01898-w PMC10244160

[459] Chen, Z. et al. Electron transport chain and mTOR inhibition synergistically decrease CD40 signaling and counteract venetoclax resistance in chronic lymphocytic leukemia. *Haematologica* **109**, 151–162 (2024). 37439352 10.3324/haematol.2023.282760 PMC10772535

[460] Gremke, N. et al. mTOR-mediated cancer drug resistance suppresses autophagy and generates a druggable metabolic vulnerability. *Nat. Commun.* **11**, 4684 (2020). 32943635 10.1038/s41467-020-18504-7 PMC7499183

[461] Lipton, J. O. & Sahin, M. The neurology of mTOR. *Neuron* **84**, 275–291 (2014). 25374355 10.1016/j.neuron.2014.09.034 PMC4223653

[462] Li, N. et al. mTOR-dependent synapse formation underlies the rapid antidepressant effects of NMDA antagonists. *Science* **329**, 959–964 (2010). 20724638 10.1126/science.1190287 PMC3116441

[463] Sato, A. et al. Rapamycin reverses impaired social interaction in mouse models of tuberous sclerosis complex. *Nat. Commun.* **3**, 1292 (2012). 23250422 10.1038/ncomms2295 PMC3535343

[464] Bercury, K. K. et al. Conditional ablation of raptor or rictor has differential impact on oligodendrocyte differentiation and CNS myelination. *J. Neurosci.* **34**, 4466–4480 (2014). 24671993 10.1523/JNEUROSCI.4314-13.2014 PMC3965777

[465] Gonçalves, F. B. & Morais, V. A. PINK1: A bridge between mitochondria and Parkinson’s disease. *Life (Basel)* **11**, 371 (2021). 33919398 10.3390/life11050371 PMC8143285

[466] Zheng, G. et al. Rapamycin alleviates cognitive impairment in murine vascular dementia: The enhancement of mitophagy by PI3K/AKT/mTOR axis. *Tissue Cell* **69**, 101481 (2021). 33383488 10.1016/j.tice.2020.101481

[467] Davoody, S., Asgari Taei, A., Khodabakhsh, P. & Dargahi, L. mTOR signaling and Alzheimer’s disease: What we know and where we are?. *CNS Neurosci. Ther.* **30**, e14463 (2024). 37721413 10.1111/cns.14463 PMC11017461

[468] Akwa, Y. et al. Synaptic activity protects against AD and FTD-like pathology via autophagic-lysosomal degradation. *Mol. Psychiatry* **23**, 1530–1540 (2018). 28696431 10.1038/mp.2017.142 PMC5641448

[469] Norambuena, A. et al. Disrupted mitochondrial response to nutrients is a presymptomatic event in the cortex of the APPSAA knock-in mouse model of Alzheimer’s disease. *Alzheimers Dement* **20**, 6844–6859 (2024). 39171353 10.1002/alz.14144 PMC11485302

[470] Norambuena, A. et al. A novel lysosome-to-mitochondria signaling pathway disrupted by amyloid-β oligomers. *EMBO J.* **37**, e100241 (2018). 30348864 10.15252/embj.2018100241 PMC6236329

[471] Davis, O. B. et al. NPC1-mTORC1 signaling couples cholesterol sensing to organelle homeostasis and is a targetable pathway in Niemann-Pick type C. *Dev. Cell* **56**, 260–276.e7 (2021). 33308480 10.1016/j.devcel.2020.11.016 PMC8919971

[472] Zeng, L.-H., Xu, L., Gutmann, D. H. & Wong, M. Rapamycin prevents epilepsy in a mouse model of tuberous sclerosis complex. *Ann. Neurol.* **63**, 444–453 (2008). 18389497 10.1002/ana.21331 PMC3937593

[473] Baldassari, S. et al. The landscape of epilepsy-related GATOR1 variants. *Genet. Med.* **21**, 398–408 (2019). 30093711 10.1038/s41436-018-0060-2 PMC6292495

[474] Honke, J. et al. Deep histopathology genotype–phenotype analysis of focal cortical dysplasia type II differentiates between the GATOR1-altered autophagocytic subtype IIa and MTOR-altered migration deficient subtype IIb. *Acta Neuropathol. Commun.* **11**, 179 (2023). 37946310 10.1186/s40478-023-01675-x PMC10633947

[475] Wang, H. et al. Seizure features and outcomes in 50 children with GATOR1 variants: A retrospective study, more favorable for epilepsy surgery. *Epilepsia Open* **8**, 969–979 (2023). 37259768 10.1002/epi4.12770 PMC10472406

[476] Yuskaitis, C. J. et al. DEPDC5-dependent mTORC1 signaling mechanisms are critical for the anti-seizure effects of acute fasting. *Cell Rep.* **40**, 111278 (2022). 36044864 10.1016/j.celrep.2022.111278 PMC9508617

[477] Du, S. et al. Functional characterization of novel NPRL3 mutations identified in three families with focal epilepsy. *Sci. China Life Sci.* **66**, 2152–2166 (2023). 37071290 10.1007/s11427-022-2313-1

[478] Zhang, X. et al. High-protein diets increase cardiovascular risk by activating macrophage mTOR to suppress mitophagy. *Nat. Metab.* **2**, 110–125 (2020). 32128508 10.1038/s42255-019-0162-4 PMC7053091

[479] Tumaneng, K. et al. YAP mediates crosstalk between the Hippo and PI(3)K–TOR pathways by suppressing PTEN via miR-29. *Nat. Cell Biol.* **14**, 1322–1329 (2012). 23143395 10.1038/ncb2615 PMC4019071

[480] Wu, W. et al. Activation of Hippo signaling pathway mediates mitochondria dysfunction and dilated cardiomyopathy in mice. *Theranostics* **11**, 8993–9008 (2021). 34522223 10.7150/thno.62302 PMC8419046

[481] Zhang, J., Liu, X., Nie, J. & Shi, Y. Restoration of mitophagy ameliorates cardiomyopathy in Barth syndrome. *Autophagy* **18**, 2134–2149 (2022). 34985382 10.1080/15548627.2021.2020979 PMC9466615

[482] Oeing, C. U. et al. MTORC1-regulated metabolism controlled by TSC2 limits cardiac reperfusion injury. *Circ. Res.* **128**, 639–651 (2021). 33401933 10.1161/CIRCRESAHA.120.317710 PMC8257748

[483] Haythorne, E. et al. Altered glycolysis triggers impaired mitochondrial metabolism and mTORC1 activation in diabetic β-cells. *Nat. Commun.* **13**, 6754 (2022). 36376280 10.1038/s41467-022-34095-x PMC9663558

[484] Zou, J. et al. Wnt inhibitory factor 1 ameliorated diabetic retinopathy through the AMPK/mTOR pathway-mediated mitochondrial function. *FASEB J.* **36**, e22531 (2022). 36063130 10.1096/fj.202200366RR

[485] Elia, D., Harari, S., Fan, L., Diesler, R. & Henske, E. P. Novel treatment strategies for lymphangioleiomyomatosis: A narrative review. *Eur. Respir. Rev.* **34**, 250019 (2025). 40769537 10.1183/16000617.0019-2025 PMC12340533

[486] Chung, C.-Y. et al. Constitutive activation of the PI3K-Akt-mTORC1 pathway sustains the m.3243 A > G mtDNA mutation. *Nat. Commun.* **12**, 6409 (2021). 34737295 10.1038/s41467-021-26746-2 PMC8568893

[487] Chung, C.-Y. et al. Inhibition of the PI3K-AKT-MTORC1 axis reduces the burden of the m.3243A>G mtDNA mutation by promoting mitophagy and improving mitochondrial function. *Autophagy* **21**, 881–896 (2025). 39667405 10.1080/15548627.2024.2437908 PMC11925111

[488] Bordi, M. et al. mTOR hyperactivation in Down Syndrome underlies deficits in autophagy induction, autophagosome formation, and mitophagy. *Cell Death Dis.* **10**, 563 (2019). 31332166 10.1038/s41419-019-1752-5 PMC6646359

[489] Serrano-Maciá, M. et al. Neddylation inhibition ameliorates steatosis in NAFLD by boosting hepatic fatty acid oxidation via the DEPTOR-mTOR axis. *Mol. Metab.* **53**, 101275 (2021). 34153521 10.1016/j.molmet.2021.101275 PMC8280515

[490] Cholewinski, T., Pereira, D., Moerland, M. & Jacobs, G. E. MTORC1 signaling as a biomarker in major depressive disorder and its pharmacological modulation by novel rapid-acting antidepressants. *Ther. Adv. Psychopharmacol.* **11**, 20451253211036814 (2021). 34733478 10.1177/20451253211036814 PMC8558816

[491] Benjamin, D., Colombi, M., Moroni, C. & Hall, M. N. Rapamycin passes the torch: a new generation of mTOR inhibitors. *Nat. Rev. Drug Discov.* **10**, 868–880 (2011). 22037041 10.1038/nrd3531

[492] Fan, Q. W., Nicolaides, T. P. & Weiss, W. A. Inhibiting 4EBP1 in Glioblastoma. *Clin. Cancer Res.* **24**, 14–21 (2018). 28696243 10.1158/1078-0432.CCR-17-0042 PMC5754225

[493] Hua, H. et al. Targeting mTOR for cancer therapy. *J. Hematol. Oncol.* **12**, 71 (2019). 31277692 10.1186/s13045-019-0754-1 PMC6612215

[494] Peng, Y., Wang, Y., Zhou, C., Mei, W. & Zeng, C. PI3K/Akt/mTOR pathway and its role in cancer therapeutics: are we making headway?. *Front. Oncol.* **12**, 819128 (2022). 35402264 10.3389/fonc.2022.819128 PMC8987494

[495] Popova, N. V. & Jücker, M. The role of mTOR signaling as a therapeutic target in cancer. *Int. J. Mol. Sci.* **22**, 1743 (2021). 33572326 10.3390/ijms22041743 PMC7916160

[496] Palm, W. et al. The utilization of extracellular proteins as nutrients is suppressed by mTORC1. *Cell* **162**, 259–270 (2015). 26144316 10.1016/j.cell.2015.06.017 PMC4506698

[497] Guba, M. et al. Rapamycin inhibits primary and metastatic tumor growth by antiangiogenesis: involvement of vascular endothelial growth factor. *Nat. Med.* **8**, 128–135 (2002). 11821896 10.1038/nm0202-128

[498] Feldman, M. E. et al. Active-site inhibitors of mTOR target rapamycin-resistant outputs of mTORC1 and mTORC2. *PLoS Biol.* **7**, e38 (2009). 19209957 10.1371/journal.pbio.1000038 PMC2637922

[499] Thoreen, C. C. et al. An ATP-competitive mammalian target of rapamycin inhibitor reveals rapamycin-resistant functions of mTORC1. *J. Biol. Chem.* **284**, 8023–8032 (2009). 19150980 10.1074/jbc.M900301200 PMC2658096

[500] Lapointe, S. et al. A phase I study of vistusertib (dual mTORC1/2 inhibitor) in patients with previously treated glioblastoma multiforme: a CCTG study. *Invest. N. Drugs* **38**, 1137–1144 (2020). 10.1007/s10637-019-00875-4 31707687

[501] Chresta, C. M. et al. AZD8055 is a potent, selective, and orally bioavailable ATP-competitive mammalian target of rapamycin kinase inhibitor with in vitro and in vivo antitumor activity. *Cancer Res* **70**, 288–298 (2010). 20028854 10.1158/0008-5472.CAN-09-1751

[502] Liu, Q. et al. Characterization of Torin2, an ATP-competitive inhibitor of mTOR, ATM, and ATR. *Cancer Res* **73**, 2574–2586 (2013). 23436801 10.1158/0008-5472.CAN-12-1702 PMC3760004

[503] Silvera, D. et al. mTORC1 and -2 coordinate transcriptional and translational reprogramming in resistance to DNA damage and replicative stress in breast cancer cells. *Mol. Cell. Biol.* **37**, e00577–16 (2017). 27956700 10.1128/MCB.00577-16 PMC5311240

[504] Sarbassov, D. D. et al. Prolonged rapamycin treatment inhibits mTORC2 assembly and Akt/PKB. *Mol. Cell* **22**, 159–168 (2006). 16603397 10.1016/j.molcel.2006.03.029

[505] Kleinert, M. et al. Acute mTOR inhibition induces insulin resistance and alters substrate utilization in vivo. *Mol. Metab.* **3**, 630–641 (2014). 25161886 10.1016/j.molmet.2014.06.004 PMC4142396

[506] Rodrik-Outmezguine, V. S. et al. Overcoming mTOR resistance mutations with a new-generation mTOR inhibitor. *Nature* **534**, 272–276 (2016). 27279227 10.1038/nature17963 PMC4902179

[507] Fan, Q. et al. A kinase inhibitor targeted to mTORC1 drives regression in glioblastoma. *Cancer Cell* **31**, 424–435 (2017). 28292440 10.1016/j.ccell.2017.01.014 PMC5386178

[508] Zhang, Z. et al. Brain-restricted mTOR inhibition with binary pharmacology. *Nature* **609**, 822–828 (2022). 36104566 10.1038/s41586-022-05213-y PMC9492542

[509] Yang, G. et al. Dissecting the biology of mTORC1 beyond rapamycin. *Sci. Signal.* **14**, eabe0161 (2021). 34546793 10.1126/scisignal.abe0161 PMC8580572

[510] Maira, S.-M. et al. Identification and characterization of NVP-BEZ235, a new orally available dual phosphatidylinositol 3-kinase/mammalian target of rapamycin inhibitor with potent in vivo antitumor activity. *Mol. Cancer Ther.* **7**, 1851–1863 (2008). 18606717 10.1158/1535-7163.MCT-08-0017

[511] Sutherlin, D. P. et al. Discovery of a potent, selective, and orally available class I phosphatidylinositol 3-kinase (PI3K)/mammalian target of rapamycin (mTOR) kinase inhibitor (GDC-0980) for the treatment of cancer. *J. Med. Chem.* **54**, 7579–7587 (2011). 21981714 10.1021/jm2009327

[512] Venkatesan, A. M. et al. Bis(morpholino-1,3,5-triazine) derivatives: potent adenosine 5’-triphosphate competitive phosphatidylinositol-3-kinase/mammalian target of rapamycin inhibitors: discovery of compound 26 (PKI-587), a highly efficacious dual inhibitor. *J. Med. Chem.* **53**, 2636–2645 (2010). 20166697 10.1021/jm901830p

[513] Howell, J. J. et al. Metformin inhibits hepatic mTORC1 signaling via dose-dependent mechanisms involving AMPK and the TSC complex. *Cell Metab.* **25**, 463–471 (2017). 28089566 10.1016/j.cmet.2016.12.009 PMC5299044

[514] Wang, Y. et al. Metformin improves mitochondrial respiratory activity through activation of AMPK. *Cell Rep.* **29**, 1511–1523.e5 (2019). 31693892 10.1016/j.celrep.2019.09.070 PMC6866677

[515] Zhang, Y. et al. Metformin interacts with AMPK through binding to γ subunit. *Mol. Cell. Biochem.* **368**, 69–76 (2012). 22644486 10.1007/s11010-012-1344-5

[516] Dasgupta, B. & Milbrandt, J. Resveratrol stimulates AMP kinase activity in neurons. *Proc. Natl. Acad. Sci. USA* **104**, 7217–7222 (2007). 17438283 10.1073/pnas.0610068104 PMC1855377

[517] Hinkle, J. S., Rivera, C. N. & Vaughan, R. A. AICAR stimulates mitochondrial biogenesis and BCAA catabolic enzyme expression in C2C12 myotubes. *Biochimie* **195**, 77–85 (2022). 34798200 10.1016/j.biochi.2021.11.004

[518] Vashisht Gopal, Y. N. et al. A novel mitochondrial inhibitor blocks MAPK pathway and overcomes MAPK inhibitor resistance in melanoma. *Clin. Cancer Res.* **25**, 6429–6442 (2019). 31439581 10.1158/1078-0432.CCR-19-0836 PMC6825560

[519] Romanino, K. et al. Myopathy caused by mammalian target of rapamycin complex 1 (mTORC1) inactivation is not reversed by restoring mitochondrial function. *Proc. Natl. Acad. Sci. USA* **108**, 20808–20813 (2011). 22143799 10.1073/pnas.1111448109 PMC3251091

[520] Liu, T. et al. Rapamycin reverses ferroptosis by increasing autophagy in MPTP/MPP+-induced models of Parkinson’s disease. *Neural Regen. Res.* **18**, 2514–2519 (2023). 37282484 10.4103/1673-5374.371381 PMC10360095

[521] Van Skike, C. E. et al. mTOR attenuation with rapamycin reverses neurovascular uncoupling and memory deficits in mice modeling Alzheimer’s Disease. *J. Neurosci.* **41**, 4305–4320 (2021). 33888602 10.1523/JNEUROSCI.2144-20.2021 PMC8143195

[522] Liu, Q. et al. AZD8055 is more effective than rapamycin in inhibiting proliferation and promoting mitochondrial clearance in erythroid differentiation. *Anal. Cell. Pathol. (Amst.)* **2024**, 2639464 (2024). 39411209 10.1155/2024/2639464 PMC11479778

[523] Holler, M. et al. Dual targeting of Akt and mTORC1 impairs repair of DNA double-strand breaks and increases radiation sensitivity of human tumor cells. *PLoS One* **11**, e0154745 (2016). 27137757 10.1371/journal.pone.0154745 PMC4854483

[524] Gil del Alcazar, C. R. et al. Inhibition of DNA double-strand break repair by the dual PI3K/mTOR inhibitor NVP-BEZ235 as a strategy for radiosensitization of glioblastoma. *Clin. Cancer Res.* **20**, 1235–1248 (2014). 24366691 10.1158/1078-0432.CCR-13-1607 PMC3947495

[525] Zhang, W. et al. Comparison of mTOR inhibitors combined with endocrine therapy versus that alone in breast cancer: a meta-analysis. *Future Oncol.* **21**, 1417–1427 (2025). 40152674 10.1080/14796694.2025.2485022 PMC12051556

[526] Hirayama, Y. et al. Anti-PD-L1 treatment enhances antitumor effect of everolimus in a mouse model of renal cell carcinoma. *Cancer Sci.* **107**, 1736–1744 (2016). 27712020 10.1111/cas.13099 PMC5198964

[527] Deken, M. A. et al. Targeting the MAPK and PI3K pathways in combination with PD1 blockade in melanoma. *Oncoimmunology* **5**, e1238557 (2016). 28123875 10.1080/2162402X.2016.1238557 PMC5215252

[528] Jiang, W. et al. Targeting PI3Kα increases the efficacy of anti-PD-1 antibody in cervical cancer. *Immunology* **170**, 419–438 (2023). 37469254 10.1111/imm.13682

[529] Masuyama, N. et al. Akt inhibits the orphan nuclear receptor Nur77 and T-cell apoptosis. *J. Biol. Chem.* **276**, 32799–32805 (2001). 11438550 10.1074/jbc.M105431200

[530] Meraz, I. M. et al. NPRL2 gene therapy induces effective antitumor immunity in KRAS/STK11 mutant anti-PD1 resistant metastatic non-small cell lung cancer (NSCLC) in a humanized mouse model. *eLife* **13**, RP98258 (2025). 39932765 10.7554/eLife.98258 PMC11813225

[531] Puente, C., Hendrickson, R. C. & Jiang, X. Nutrient-regulated phosphorylation of ATG13 inhibits starvation-induced autophagy. *J. Biol. Chem.* **291**, 6026–6035 (2016). 26801615 10.1074/jbc.M115.689646 PMC4786734

[532] Kim, Y.-M. et al. mTORC1 phosphorylates UVRAG to negatively regulate autophagosome and endosome maturation. *Mol. Cell* **57**, 207–218 (2015). 25533187 10.1016/j.molcel.2014.11.013 PMC4304967

[533] Yu, Y. et al. Phosphoproteomic analysis identifies Grb10 as an mTORC1 substrate that negatively regulates insulin signaling. *Science* **332**, 1322–1326 (2011). 21659605 10.1126/science.1199484 PMC3195509

[534] Battaglioni, S., Craigie, L.-M., Filippini, S., Maier, T. & Hall, M. N. mTORC1 phosphorylates and stabilizes LST2 to negatively regulate EGFR. *Proc. Natl. Acad. Sci. USA* **121**, e2405959121 (2024). 39141345 10.1073/pnas.2405959121 PMC11348030

[535] Burnett, P. E., Barrow, R. K., Cohen, N. A., Snyder, S. H. & Sabatini, D. M. RAFT1 phosphorylation of the translational regulators p70 S6 kinase and 4E-BP1. *Proc. Natl. Acad. Sci. USA* **95**, 1432–1437 (1998). 9465032 10.1073/pnas.95.4.1432 PMC19032

[536] Batool, A. et al. Eukaryotic initiation factor 4E is a novel effector of mTORC1 signaling pathway in cross talk with Mnk1. *Mol. Cell. Biochem.* **465**, 13–26 (2020). 31782083 10.1007/s11010-019-03663-z

[537] Dai, N. et al. mTOR phosphorylates IMP2 to promote IGF2 mRNA translation by internal ribosomal entry. *Genes Dev.* **25**, 1159–1172 (2011). 21576258 10.1101/gad.2042311 PMC3110954

[538] Jung, S. H. et al. mTOR kinase leads to PTEN-loss-induced cellular senescence by phosphorylating p53. *Oncogene* **38**, 1639–1650 (2019). 30337688 10.1038/s41388-018-0521-8 PMC6755978

[539] Smiles, W. J. et al. AMPK phosphosite profiling by label-free mass spectrometry reveals a multitude of mTORC1-regulated substrates. *NPJ Metab. Health Dis.* **3**, 8 (2025). 40052110 10.1038/s44324-025-00052-7 PMC11879883

[540] Li, T. et al. PKM2 coordinates glycolysis with mitochondrial fusion and oxidative phosphorylation. *Protein Cell* **10**, 583–594 (2019). 30887444 10.1007/s13238-019-0618-z PMC6626593

[541] Duan, S. et al. mTOR generates an autoamplification loop by triggering the βTrCP- and CK1α-dependent degradation of DEPTOR. *Mol. Cell* **44**, 317–324 (2011). 22017877 10.1016/j.molcel.2011.09.005 PMC3212871

[542] Baskaran, P. et al. Phosphorylation of the novel mTOR substrate Unkempt regulates cellular morphogenesis. *J. Biol. Chem.* **299**, 102788 (2023). 36509146 10.1016/j.jbc.2022.102788 PMC9852543

[543] Chen, W. et al. Regulation of a third conserved phosphorylation site in SGK1. *J. Biol. Chem.* **284**, 3453–3460 (2009). 19068477 10.1074/jbc.M807502200 PMC2635031

[544] Yin, Y. et al. mTORC2 promotes type I insulin-like growth factor receptor and insulin receptor activation through the tyrosine kinase activity of mTOR. *Cell Res* **26**, 46–65 (2016). 26584640 10.1038/cr.2015.133 PMC4816127

